# Transcriptional changes in muscle of hibernating arctic ground squirrels (*Urocitellus parryii*): implications for attenuation of disuse muscle atrophy

**DOI:** 10.1038/s41598-020-66030-9

**Published:** 2020-06-02

**Authors:** Anna V. Goropashnaya, Brian M. Barnes, Vadim B. Fedorov

**Affiliations:** Institute of Arctic Biology, University of Alaska Fairbanks, Fairbanks, AK 99775-7000 USA

**Keywords:** Functional genomics, Gene expression, Transcriptomics

## Abstract

Physical inactivity generates muscle atrophy in most mammalian species. In contrast, hibernating mammals demonstrate limited muscle loss over the prolonged intervals of immobility during winter, which suggests that they have adaptive mechanisms to reduce disuse muscle atrophy. To identify transcriptional programs that underlie molecular mechanisms attenuating muscle loss, we conducted a large-scale gene expression profiling in quadriceps muscle of arctic ground squirrels, comparing hibernating (late in a torpor and during torpor re-entry after arousal) and summer active animals using next generation sequencing of the transcriptome. Gene set enrichment analysis showed a coordinated up-regulation of genes involved in all stages of protein biosynthesis and ribosome biogenesis during both stages of hibernation that suggests induction of translation during interbout arousals. Elevated proportion of down-regulated genes involved in apoptosis, NFKB signaling as well as significant under expression of atrogenes, upstream regulators (FOXO1, FOXO3, NFKB1A), key components of the ubiquitin proteasome pathway (FBXO32, TRIM63, CBLB), and overexpression of PPARGC1B inhibiting proteolysis imply suppression of protein degradation in muscle during arousals. The induction of protein biosynthesis and decrease in protein catabolism likely contribute to the attenuation of disuse muscle atrophy through prolonged periods of immobility of hibernation.

## Introduction

Prolonged physical inactivity leads to loss of muscle mass and strength in most mammalian species. An exception are hibernators that remain largely physically inactive during 6–8 months each year yet show much less muscle atrophy than would be anticipated^1^. This suggests that diverse species of hibernating mammals have evolved adaptations that reduce muscle atrophy during prolonged inactivity and preserve locomotor ability throughout hibernation.

**Figure 1:**
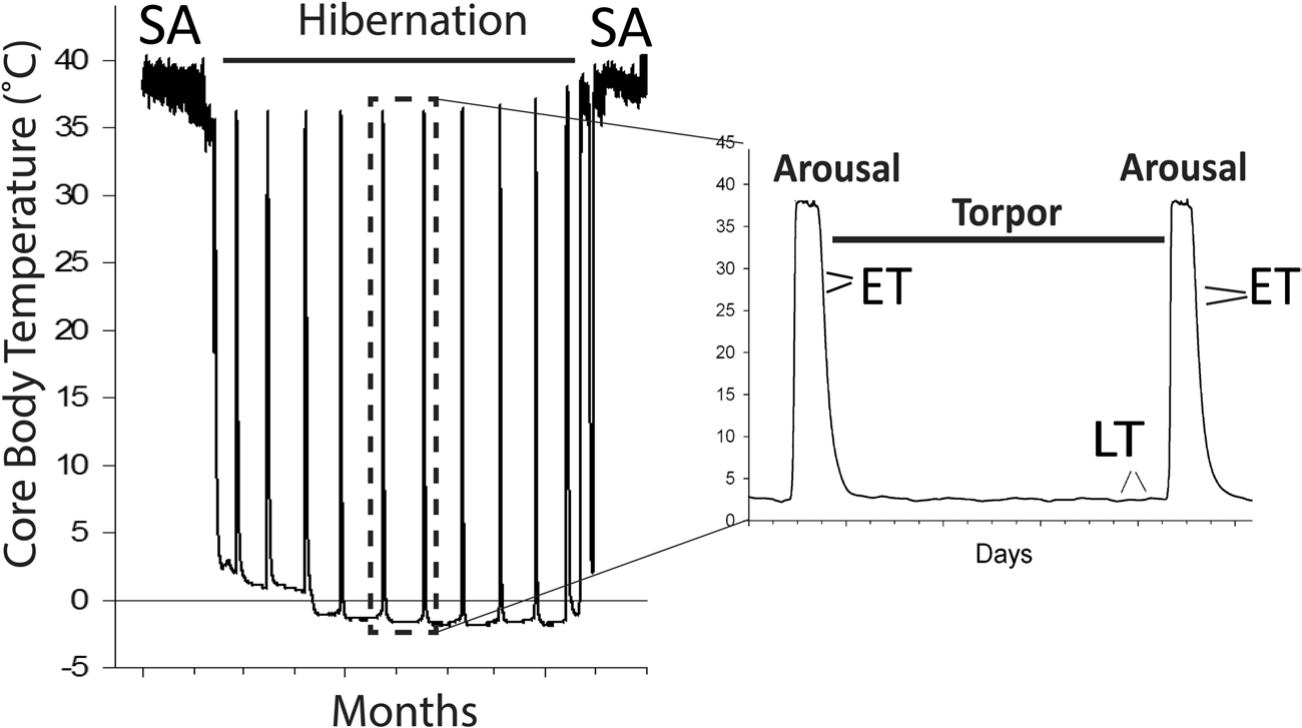
Time points of tissue sampling in arctic ground squirrels: ET – entering torpor, LT – late torpor, SA – summer active animals.

Small hibernators (<1 kg) such as ground squirrels show little or no locomotion during winter hibernation that can extend over 8 months^2^. They enter 2–3 week periods of deep torpor with body temperatures near 0 °C and a 98% reduction in whole animal metabolic rate (Fig. 1). Torpor is periodically interrupted by spontaneous arousal episodes when animals raise their metabolism and body temperature returns to euthermic levels above 36 °C for less than 24 h^3^. Several species of ground squirrels demonstrate relatively minor changes in cross sectional morphology and limited decreases in muscle mass and protein content compared to non-hibernating species when both experience prolonged immobility^1,4–8^. The molecular mechanisms underlying this protective musculoskeletal adaptation to disuse remain poorly understood.

Several studies on two other species of hibernating ground squirrels reported expression changes at the protein level of individual genes involved in signaling pathways that regulate muscle mass^5,6,9^. Detected changes in proteins quantities and phosphorylation suggest that maintaining protein synthesis through activation of the mTOR signaling pathway^5^, increase in oxidative capacity^9^ and inhibition of the calpain protein degradation^6^ in hibernating muscle likely contribute to muscle atrophy prevention.

The first comparative study of transcriptional changes on a genomic scale (9,147 genes) in skeletal muscle of hibernators revealed an elevated expression of genes involved in protein biosynthesis and ribosome biogenesis pathways, but no coordinated directional changes of genes within protein catabolic pathways in arctic ground squirrels or hibernating black bears^10^. These findings are in contrast to the reduction of protein biosynthesis and elevated expression of catabolic genes that are commonly detected in atrophying muscle of traditional mammalian models of disuse^11^.

These results led to the working hypothesis that an elevated expression of genes involved in protein biosynthesis contributes to molecular mechanisms preserving muscle mass and strength during the long intervals of physical inactivity during hibernation. However, the low genome coverage associated with custom cDNA microarray approaches used initially in these non- model species reduced the resolving power of metabolic and signaling pathway analysis. Another limitation of the previous study is that transcriptional changes were assessed in muscle sampled from arctic ground squirrels at low body temperatures during torpor when mRNA translation is inhibited^12^. Hence, quantities of transcripts measured during torpor will not reflect the amount of proteins defining biological function. We suggested that transcriptional elevation of protein biosynthesis genes detected in torpid squirrels facilitates induction of translation in muscle during the interbout arousals when hibernators briefly return to high body temperature^10^.

Support for the importance of protein biosynthesis for muscle maintenance comes from more recent genome-wide (8,278 genes) transcriptome sequencing study in muscle of hibernating 13-lined ground squirrels showing elevated expression of genes involved in the IGF1-Akt-mTOR signaling pathway that implies activation of protein biosynthesis during arousal as a factor contributing to muscle preservation^13^. In addition, transcriptional reduction of several upstream regulators of the proteasome pathway suggests that decrease in protein degradation maintains muscle mass under disuse of hibernation^13^.

In this study, to overcome limited genome coverage we conducted unbiased screen of transcriptome by using the next generation sequencing (RNA-seq) to assess expression changes in muscle of hibernating arctic ground squirrels sampled during torpor at low body temperature and at high body temperature at the end of an arousal, with comparisons to summer active animals. First, we applied discovery driven approach and generated data on expression differences for 12,000 genes (Table 1) to carry out pathway analysis identifying functional groups of co-regulated genes to infer the biological significance of transcriptional changes in skeletal muscle. Second, we implemented knowledge-based approach by selecting from our data set differentially expressed genes with known functional relation to muscle atrophy in non-hibernating mammals and statistically tested predicted downstream effects of observed transcriptional changes on muscle atrophy in hibernating arctic ground squirrels.

**Table 1:** Number of differentially expressed genes in all comparisons in muscle of arctic ground squirrels sampled at late torpor (LT), entering torpor (ET) and summer active (SA) control.

Comparison	LT vs. ET	ET vs. SA	LT vs. SA
Total number of expressed genes detected across samples	12,042	11,956	12,085
Number of differentially expressed genes (FDR < 0.05)	173	383	628
Number of up-regulated genes	114 (LT↑)	135 (ET↑)	271 (LT↑)
Number of down-regulated genes	59 (LT↓)	248 (ET↓)	357 (LT↓)
Fold change range	1.85–60.05	1.89–32.92	1.70–1034.69

## Results

Out of 40 million paired-end sequencing reads obtained for each muscle sample collected from arctic ground squirrels, 67% – 76% were mapped in pairs and 75% – 85% of mapped reads were aligned to exons of the 13-lined ground squirrel genome assembly. Thus, mapping RNA-seq reads to an annotated reference genome of a closely related species provides reasonable results for genome wide screening of transcriptional changes.

### Differentially expressed genes

Transcriptome sequencing detected the expression of 12,000 genes across all samples, about 50% of annotated genes in the reference genome (Table 1). A total of 1,184 genes were differentially expressed in at least one out of three pairwise comparisons (Table S1). Most of differentially expressed genes (64% in LT, 57% in ET) were down-regulated during hibernation compared to summer active (SA) animals. In contrast, only 35% of differentially expressed genes were down- regulated in late torpor (LT) compared to entering torpor (ET) after an arousal.

### Gene set enricment analysis

Gene set enrichment analysis (GSEA) estimates significance of elevated proportions of over or underexpressed genes among genes involved in specific biological processes or pathways as compared to random expectation^14^. GO biological process category of translation (protein biosynthesis) as well as gene sets separately involved in three stages of translation (initiation, elongation and termination) were significantly enriched by overexpressed genes in muscle of hibernating ground squirrels (Table 2). Notably, comparing between late torpor and entering torpor, before and after an arousal, showed significant decreases in the proportion of overexpressed genes involved in translation and translation initiation.

**Table 2:** Gene set enrichment for Gene Ontology category Translation (GO:0006412) and its sub-categories in the comparisons between the physiological states in the arctic ground squirrel: ET- entering torpor, LT – late torpor, SA – summer active.

Category	ET vs. SA			LT vs. SA			LT vs. ET		
	#genes	NES	FDR	#genes	NES	FDR	#genes	NES	FDR
Translation	892	3.03	<0.001	896	3.81	<0.001	893	3.08	<0.001
T. Initiation	132	3.87	<0.001	132	4.75	<0.001	132	2.99	<0.001
T. Elongation	104	2.60	0.008	104	2.56	0.004	104	−2.44	0.067
T. Termination	90	2.40	0.031	90	2.49	0.006	103	−2.39	0.069

NES – normalized enrichment score, positive values indicate elevated proportion of over-expressed genes and negative excess under-expressed genes. FDR – false discovery rate.

GSEA identified other functional gene sets significantly enriched by genes with the same direction of expressional changes (Tables 3 and S2) in muscle of hibernating squirrels.

**Table 3:** Gene set enrichment for selected Gene Ontology (GO) biological processes, Reactome (R), KEGG (K) and Hallmark (H) gene sets in muscle of arctic ground squirrels during hibernation (ET- entering torpor, LT-late torpor) as compared to summer active (SA) animals.

Comparison	ET vs. SA			LT vs. SA		
Gene set	#genes	NES	FDR	#genes	NES	FDR
Fatty acid β oxidation (GO)	45	2.27	0.05	45	2.69	0.001
Muscle atrophy (GO)	13	−1.66	0.04	13	−1.61	0.04
Translation (R)	137	4.20	<0.001	137	5.10	<0.001
Muscle contraction (R)	42	−2.94	<0.001	43	−2.69	<0.001
Ribosome (K)	81	4.09	<0.001	81	4.06	<0.001
Peroxisome (H)	80	1.91	0.034	82	2.53	<0.001
TNFA Signaling via NFKB (H)	144	−3.40	<0.001	147	−2.75	<0.001
Apoptosis (H)	127	−2.84	<0.001	129	−1.17	NS

NES – normalized enrichment score, positive values indicate elevated proportion of overexpressed genes and negative scores indicate excess of under expressed genes. FDR – false discovery rate.

During hibernation, the proportion of overexpressed genes was significantly elevated for the gene set involved in fatty acid oxdation and the related category of peroxiosome biogenesis, an important organelle for fatty acid catabolism.

Reactome gene set for translation and related set of ribosome biogenesis were encriched by up-regulated genes at both stages of hibernation. Down-regulated genes were over represented in muscle atrophy, muscle contraction GO categories and TNFA Signaling via NFKB pathway. Apoptosis gene set was significantly enriched by underexpressed genes during entering torpor.

### Differential expression of genes involved in muscle atrophy

To assess effect of differential expression of genes known to be associated with muscle atrophy in non hibernating mammals, we conducted IPA downstream effects analysis in ground squirrels sampled during torpor entrance at the end of an arousal.

**Figure 2:**
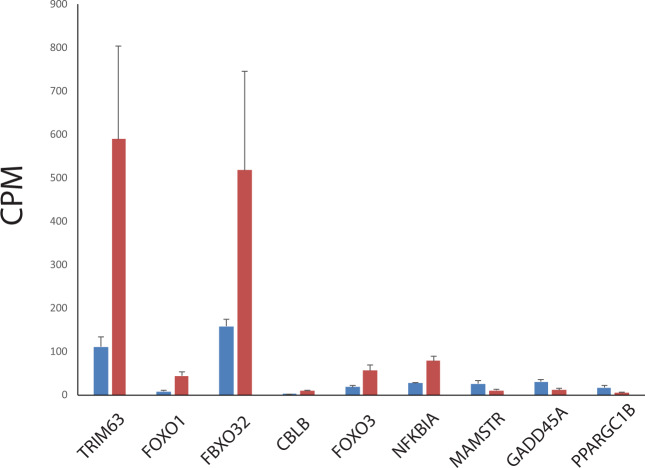
Normalized expression values of selected atrogenes (Table 4) in muscle of arctic ground squirrels during entering torpor (blue bars) and summer (red bars). CPM –the mean read count per million RNA-seq reads and its standard deviation.

Translation does occur during interbout arousals^12^ and quantities of transcripts represent proxies for protein expression that defines intensity of biological function. Genes differentially expressed (FDR < 0.05) during entering torpor comparing to summer controls (Table S1) were used as input for IPA downstream effects analysis. Muscle atrophy gene set is significantly enriched by differentially expressed genes (FDR < 0.0004) and the direction of expressional changes predicts inhibition of muscle atrophy (z score = −2.097) during torpor entry. Eight out of nine genes with reported directional effects on muscle atrophy in mammalian disuse models demonstrated expressional changes consistent with decrease in muscle atrophy in hibernating squirrels (Fig. 2, Table 4). Six genes known to induce muscle atrophy are significantly down-regulated in muscle of hibernating squirrels. These genes involved in proteasomal protein degradation include transcription factors FOXO1 and FOXO3 that activate atrophy-related ubiquitin ligases FBXO32 (also known as atrogin-1, MAFbx) and TRIM63 (also known as MuRF1) as well as ubiquitin ligase CBLB and transcription factor NFKBIA inducing muscle wasting. Two up-regulated genes, PPARGC1B and MAMSTR reduce muscle atrophy by suppressing proteolytic ubiquitin ligases and inducing myogenesis. Opposite to expression changes in other atrophy inducing genes, growth arrest and DNA damage-inducible protein (GADD45a) was up regulated in muscle during entering torpor.

**Table 4:** Results of IPA downsream effects analysis for muscle atrophy genes in arctic ground squrrels at torpor entry as compared to summer active animals.

Genes in dataset	Prediction (based on expr change direction)	Expr Fold Change (this study)	Findings (reported in references)	Reference
**PPARGC1B**	**Decreased**	2.877	Decreases	^40^
**GADD45A**	**Increased**	2.667	Increases	^41^
MET	Affected	2.582	Affects	^54^
**MAMSTR**	**Decreased**	2.380	Decreases	^55^
NFKBIA*	**Decreased**	−2.849	Increases	^39^
FOXO3*	**Decreased**	−3.022	Increases	^35^
GDF11	Affected	−3.148	Affects	^56^
**CBLB**	**Decreased**	−3.152	Increases	^38^
**FBXO32**	**Decreased**	−3.278	Increases	^34^
DHTKD1	Affected	−3.672	Affects	NCBI, CLINVAR
ATF3*	Affected	−3.988	Affects	^57^
HSPB1*	Affected	−4.284	Affects	^58^
FOXO1*	**Decreased**	−4.876	Increases	^35^
TRIM63*	**Decreased**	−5.283	Increases	^34^
CFLAR*	Affected	−5.747	Affects	^59^

Genes with known effects on muscle atrophy are in bold, asterics indicate genes with significant transcriptional changes of the same direction at late torpor.

Recent studies on other species of hibernating ground squirrels reported expression changes at the protein level of several genes involved in signaling pathways that regulate muscle mass^5,6,9^. We used our data set (Table S1) to quantify differential expression of selected genes at transcriptional level in muscle of hibernating arctic ground squirrels. Gene SGK1 shown to maintain muscle mass during hibernation was one of the most under expressed genes during both late torpor (FC = −6.88; FDR = 1.01E-10) and entering torpor (FC = −6.08; FDR = 7.46E-7). No significant transcriptional changes were detected for PPARGC1A (also known as PGC1α), a mediator in muscle remodeling, and calpastatin (CAST), negative regulator of calpain- protein degradation, during hibernation.

Notably, in our data set, several genes involved in muscle mass maintenance demonstrated significant differences in expression consistent to transcriptional changes reported for muscle of hibernating 13-lined ground squirrels^13^. All these genes were down-regulated during entering torpor (Table S1) and include two inhibitors of the mTOR signaling pathway DDIT4 (FC = −32.90; FDR = < 0.001), KLF15 (FC = −8.42; FDR = 1.8E-6) as well as two positive regulators of proteasomal protein degradation ZFAND5 (FC = −2.28; FDR = 0.009) and TNFRSF12A (FC = −4.77; FDR = 5.8E-6).

## Discussion

Our study reveals a suite of transcriptional changes in genes of arctic ground squirrels that likely contribute to attenuation of muscle atrophy during the prolonged physical inactivity of hibernation. These changes, directly related to preservation of muscle mass, include an elevated proportion of overexpressed genes involved in all stages of protein biosynthesis and ribosome biogenesis as well as transcriptional suppression of apoptotic and catabolic genes, key components of proteolytic pathways related to muscle atrophy.

Loss of muscle protein and decrease in protein synthesis are prominent features in model animals of muscle disuse atrophy^15–17^. We previously detected the coordinated transcriptional induction of multiple protein biosynthesis and ribosome biogenesis genes in muscle of black bears and arctic ground squirrels during hibernation and suggested that elevated protein biosynthesis contributes to muscle preservation^10,18^. This hypothesis has been complicated, however, by the several lines of evidence that translation of mRNA is inhibited in tissues of hibernating ground squirrels at the low body temperatures of torpor^12,19,20^. We suggested that the up-regulation of protein biosynthesis transcripts detected in muscle of torpid squirrels likely leads to their translation as body temperatures increase during the periodic, short term arousal episodes^10^.

The present study reveals the elevated expression of genes involved in all stages of protein biosynthesis during two hibernating states, late and torpor re-entry after arousal, as compared to summer active animals. A relatively high proportion (65%) of significantly up regulated genes detected by direct count of RNA-seq reads in late torpor comparing to entering torpor implies active transcription through period between re-entry and late torpor. Transcripts are protected from degradation during torpor by RNA binding proteins, and, thus, are available for translation and elevated protein synthesis during short euthermic episodes of arousals^19^. Comparison between late torpor and the re-entry into torpor after arousal showed a significant decrease in proportion of over expressed genes involved in translation initiation which was reported to cease first and uncouple from continuing elongation during entrance into torpor in the golden mantled ground squirrel^20^. This is consistent with transcript accumulation during low temperature torpor and depletion as they are translated during arousals^21^. In line with protein biosynthesis genes, transcriptional induction of genes complementing ribosome reported here implies increase in translational capacity during arousal that contributes to protective mechanisms against muscle loss^22^.

Consistent with transcriptional up-regulation reported here, large – scale proteomic study revealed significant enrichment of translation and ribosome biogenesis categories by overexpressed genes in the liver during arousal compared to torpid and summer active arctic ground squirrels^23^. Further support for induction of protein biosynthesis during interbout arousals comes from increase in incorporation rates of labeled amino acids^24^ and activation of muscle protein synthesis at this stage of hibernation detected in mid-winter for 13-lined squirrels^8^.

Several studies at protein^5^ and transcriptional^13^ levels suggest the importance of the mTOR signaling pathway for maintaining protein synthesis in muscle of hibernating 13-lined ground squirrels. Although we did not find coordinated transcriptional changes of genes involved in the mTOR signaling during the re-entry into torpor after arousal, two inhibitors of the mTOR, DDIT4 and KLF15 were significantly down-regulated, similar to findings reported for hibernating 13-lined ground squirrels^13^.

During winter while hibernating animals fast, elevated protein biosynthesis in skeletal muscle may be supplied with amino acids resulting from catabolism of non-myofibrillar protein stored in other tissues^25^. Small mammalian hibernators such as prairie dogs demonstrate a 23% reduction in protein content in liver during winter^26^. Amino acids from the breakdown of labile protein reserves likely support protein biosynthesis during arousals leading to the prevention of skeletal muscle loss.

A comprehensive set of genes involved in apoptosis is significantly enriched by down-regulated genes at torpor re-entry following arousals as compared to summer active animals. Apoptosis occurs during muscle atrophy and likely contributes to the loss of muscle mass, but the mechanisms of this process remain unknown^27^. Transcriptional suppression of apoptosis genes suggests a decrease in apoptotic activity that enhances myocytes survival and potentially reduces muscle loss^28^.

In addition to decreases in protein synthesis, induction of protein catabolism is an important mechanism leading to muscle atrophy during disuse in non-hibernating mammals^11,15,17^.

Nuclear factor kappa B signaling (NFKB) is a significant modulator of muscle atrophy in non-hibernating mammals^29^. NFKB activation by proinflammatory cytokine, tumor necrosis factor alfa (TNFA), stimulates the production of extracellular proteases and induces loss of skeletal muscle^29^. Coordinated transcriptional reduction of genes regulated by NFKB signaling in response to TNFA reported here for muscle of hibernating squirrels implies a decrease in NFKB mediated protein degradation.

In addition, it has been shown that activation NFKB signaling by another cytokine, TNF-like weak inducer of apoptosis (TWEAK) increase muscle atrophy by elevating expression of FBXO32 and TRIM63^30^, ubiquitin ligases in the proteolysis pathway, both of them down-regulated here. Significant transcriptional suppression of the TWEAK receptor TNFRSF12A during entering torpor, similar to finding in muscle of hibernating 13-lined squirrels^13^, suggests decrease in pro-atrophic effect of NFKB signaling as possible mechanism of muscle protein preservation.

The ubiquitin proteasome pathway plays a major role in muscle protein degradation during disuse^31,32^. Similar to protein biosynthesis, ubiquitin proteolysis is suppressed at the low body temperatures of torpor but activated in arousals^33^. Our study reveals significant transcriptional reduction for several atrogenes, key components in enhancing efficacy of the ubiquitin proteolysis causing muscle loss.

Muscle-specific E3 ubiquitin ligases, FBXO32 and muscle RING finger 1 protein TRIM63 are positively regulated by FOXO1 and FOXO3 transcription factors and their mRNA expression levels are increased under disuse muscle atrophy in non-hibernating mammals^34–36^. Another downstream target of FOXO transcriptional factors, ZFAND5 delivers and attaches ubiquitinated proteins to proteasomes^37^. Significant under expression of all these atrogenes suggests direct down-regulation of protein degradation in muscle of hibernating arctic ground squirrels. Ubiquitin ligase CBLB and transcription factor NFKBIA inducing muscle wasting^38,39^ are also significantly down-regulated in muscle of arctic ground squirrels during entering torpor. Consistent to suppression of the ubiquitin proteolysis, PPARGC1B inhibiting the ubiquitin proteasome and autophagic lysosome degradation pathways^40^ through transcriptional suppression of FOXO3 and NFKBIA (both down-regulated here), was overexpressed during entering torpor.

Opposite to expression pattern of other atrogenes in this study, GADD45A promoting muscle loss was up-regulated during hibernation. Although the mechanism by which GADD45A promotes muscle atrophy is unknown, expression of this gene is mediated by stress inducible pro-atrophy transcription factor ATF4^41^. However, ATF4 is not differentially expressed in muscle of hibernating ground squirrels. Thus, significance of the ATF4/GADD45A pathway for muscle atrophy in hibernating squirrels remains uncertain.

Because of direct functional relation of FBXO32, TRIM63 ubiquitin ligases and their upstream regulators, FOXO transcriptional factors to muscle atrophy in mammalian models, several studies assessed the role of their expression changes for muscle maintenance in other species hibernating ground squirrels. Although transcriptional suppression of FOXO1 and some increase in FBXO32 expression were previously reported in muscle of torpid golden-mantled ground squirrels^42^, other studies did not detect expression changes of FBXO32 and TRIM63 in muscle of hibernating13–lined and daurian ground squirrels^5,43^. Stable expression of two atrogenes was considered as a factor preserving muscle mass during inactivity of hibernation^43^. Studies at protein level showed decrease of FBXO32 and TRIM63 expression^44^ but some increase of FOXO3 expression^45^ at several time points during low temperature torpor in muscle of 13-lined squirrel. This reduction of FBXO32 and TRIM63 protein quantities is consistent with temperature dependent suppression of the ubiquitin proteolysis at torpor^33^.

The first genome-wide transcriptome sequencing in muscle of hibernating 13-lined squirrels^13^ showed significant down-regulation of several protein degradation genes including transcription factors FOXO1 and FOXO3, cytokine receptor TNFRSF12A and proteasome anchoring protein ZFAND5. Similar to 13-lined squirrels, all these genes are significantly under expressed in muscle arctic ground squirrels during entering torpor after arousal. In addition, our study detected coordinated transcriptional changes for 13 more genes (Table 4) related to pro-atrophic protein degradation and statistically tested predicted downstream effect of these changes that suggests decrease in atrophic response. In sum, transcriptional suppression of apoptosis, NFKB signaling, key genes in the ubiquitin proteasome pathway and induction of genes inhibiting proteolysis reported here imply a decrease in muscle protein degradation during arousals.

We found a coordinated induction of genes involved in fatty acid β oxidation in muscle during both stages of hibernation that implies elevated utilization of fat in hibernating muscle. The proportion of up-regulated genes related to peroxisome, a key organelle for fatty acid oxidation, is also increased during hibernation. Induction of lipid catabolism and fatty acid β oxidation has been reported at transcriptional^21,46^ and proteomic levels^23^ in the liver of hibernating arctic ground squirrels and black bears. These transcriptional changes are consistent with physiological data showing that hibernating mammals maintain whole body metabolism by using primarily energy stored in fat instead of carbohydrates^12^. In skeletal muscle of 13-lined squirrels, transcriptional induction^13^ and increased quantities of proteins^47^ involved in fatty acid oxidation also supports the importance of lipids as fuel in muscle metabolism during hibernation.

Significant transcriptional suppression of genes involved in muscle contraction was detected during both hibernation stages and reflects low muscle loading in winter. This finding does not provide any evidence for increases in muscular activity during arousal that could potentially contribute to maintenance of muscle mass^1,42^.

This study reveals a coordinated increase in transcription of anabolic genes involved in translation that implies an induction of protein biosynthesis during euthermic arousals. Elevated proportion of under expressed genes involved in apoptosis and NFKB signaling pathways as well as significant down-regulation of atrogenes, upstream regulators and key components of the ubiquitin proteasome pathway indicate suppression of protein degradation in muscle during arousals. Induction of protein biosynthesis and reduction of catabolism likely contribute to the attenuation of disuse muscle atrophy through prolonged periods of immobility and fasting that characterize hibernation. These conclusions are based on genome – wide transcriptional changes representing proxies for changes in protein expression that define intensity of biological function. Although post-transcriptional regulation decreases correlation between protein and transcript levels^48^, our inference on functional significance of transcriptional changes is supported by positive correlation (Pearson’s r = 0.62; P < 0.001) between expression changes on the transcriptional and protein levels that was reported in comparison between summer active and hibernating arctic ground squirrels^23^.

## Material and Methods

### Animals

Arctic ground squirrels were trapped in two locations in Northern part of Alaska (100 km north of Fairbanks and West of Pump Station #4, north of the Brooks Range) and transported to the University of Alaska Fairbanks. Squirrels were individually housed initially at 20 ± 2 °C with a 16 h: 8 h light: dark photoperiod and provided with Mazuri Rodent Chow and water *ad libitum* with supplements of sunflower seeds, carrots and apples. In late September animals with abdominally implanted with temperature-sensitive radio transmitters and transferred into an environmental chamber with +2 °C temperature and 4 h: 20 h light: dark. Squirrels were provided with ample cotton for constructing nests and rodent chow, water and carrots until they first entered torpor, then all food was removed. Core body temperature (Tb) was monitored to detect stages of torpor and arousal by an automated telemetry system that measured and recorded core Tb every 10 min^49^. All animals sampled during hibernation had completed at least three full-length torpor bouts. Four animals were sampled during re-entry into torpor (Tb = 27 ± 1 °C, Fig. 1) following an interbout arousal and four animals during late in a torpor bout (Tb = 2.2 ± 0.3 °C, after 80–90% of the duration of a bout, 8–12 days). Four summer active squirrels were sampled in July after completion of reproductive regression as assessed by external inspection of gonads and genitalia. The mean body weight was 613.75 ± 48.69 g during hibernation and 719.00 ± 144.83 g for summer active squirrels but the difference is nonsignificant (P = 0.22). To decrease biological variation, animals included in the study were all adult males with the exception of one female in the entering torpor hibernation group. Torpid animals were euthanized by decapitation without anesthesia, summer active animals were deeply anesthetized with isoflurane vapors, entering torpor animals were anesthetized with sodium pentobartitol before decapitation. Quadriceps skeleton muscle tissue was rapidly dissected and frozen in liquid nitrogen within 9 min of death and stored at −80 °C until RNA extraction. All experiments were carried out in accordance with animal protocols approved by the University of Alaska Fairbanks, Institutional Animal Care and Use Committee (IACUC number 569666).

### RNA isolation and sequencing

Frozen muscle samples (approximately 250 mg) were homogenized directly in 2 ml Lysing Matrix D tubes with specialized beads and RTL buffer using a Mini-Beadbeater-1 (BioSpec Products, Inc., Bartlesville, OK, USA) for 1 min at 4800 oscillations/minute. Total RNA was isolated from the tissue using RNeasy mini kit (Qiagen Inc., Valencia, CA, USA). All RNA samples received a DNase I (Qiagen) treatment to remove DNA contamination. The RNA quality and concentration were obtained with an Agilent 2100 Bioanalyzer and a Nanodrop ND-1000. Then, the total RNA samples were used for cDNA library construction and sequencing 40 million of 100 nucleotide paired-end reads for each sample on Illumina HiSeq. 4000 system at BGI Americas Corporation (Cambridge, MA).

### Data analysis

RNA-seq reads mapping, reads counting and differential gene expression analysis was conducted using CLC Genomics Workbench (v10, https://www.qiagenbioinformatics.com). Paired-end sequencing reads were mapped to the reference genome of *Ictidomis tridecemlineatus* (13-lined ground squirrel, NCBI assembly SpeTri2.0; AGTP00000000.1). After preliminary tests the following parameters were used for the alignment: mismatch cost: 2, insertion cost: 3, deletion cost: 3, similarity fraction: 0.7, length fraction: 0.7, max number of hits for a read: 10. Total of 38, 592 proteins and 25,998 genes are annotated in the reference genome, so for mapping sequence reads we used the option “Genome annotated with genes and transcripts”. Total counts of reads mapped in pairs to the exons were used as an expression values and normalized for library size with the TMM method^50^. The dispersion parameter of normalized read counts for each gene was estimated using negative binomial Generalized Linear Model as implemented in the multi-factorial EdgeR methods^51^ and Wald test was applied for comparisons of all group (LT, ET, and SA) pairs. Only genes with at least 2 paired reads across all samples in a pairwise comparison were included in the analysis. The false discovery rate (FDR) for each gene was estimated using the procedure described by Benjamini and Hochberg^52^. Genes were considered differentially expressed if FDR was 0.05 or less.

We estimated enrichment in gene sets corresponding to biological function or metabolic, signaling pathways using Gene Set Enrichment Analysis (http://software.broadinstitute.org/gsea). GSEA estimates overrepresentation of up or down-regulated genes by taking into consideration all of the genes with expression detected in an experiment^14^ and both over expressed and under expressed genes are analyzed in the same run to obtain integrative estimate of enrichment. Genes were pre-ranked according to their fold change between their expression values in pairwise comparisons between the phenotype classes (late torpor, entering torpor and summer active phenotypes). An enrichment score (ES) that reflects the degree to which genes involved in a gene set are overrepresented at the extremes (up-regulated genes at the top and down-regulated genes at the bottom) of the entire ranked list of genes was calculated. Although all genes in experiments are analyzed to estimate general trend of enrichment, differentially expressed genes form the enrichment core. The ES was normalized to account for the size of the gene sets presented in the experiment, yielding a normalized enrichment score (NES). The positive values of the NES indicate enrichment by overexpressed genes and the negative values suggest elevated proportion of under expressed genes. The statistical significance of the NES was estimated by calculating the false discovery rate using gene set-based permutation test. Gene sets corresponding to biological functions and processes were obtained from Molecular Signatures Database (www.broadinstitute.org/gsea/msigdb/index.jsp) and included the following collections: Gene Ontology Biological Processes, Biocarta, Hallmark, KEGG and Reactome (Table S2).

To predict downstream effects of observed transcriptional changes on muscle atrophy we used the causal network approach as implemented in downstream effects analysis of Ingenuity Pathway Analysis (IPA, http://www.ingenuity.com). Unlike GSEA, only differentially expressed genes (FDR < 0.05) detected in pairwise comparisons (Table S1) were used as input for IPA. Enrichment by differentially expressed genes was estimated by Fisher’s exact test and a Z-score assessing the match of observed direction of expression changes and predicted up/down regulation patterns was calculated^53^. Set of genes shown to be related to muscle atrophy in non-hibernating mammals and prediction of downstream effect were informed by the IPA Knowledge Base.

## Supplementary information

Supplementary Information.

## Data Availability

Transcriptome sequencing data were archived on the NCBI Short Read Archive (NCBI: PRJNA481834).
