# Supplementary material for: Transcriptional changes in muscle of hibernating arctic ground squirrels (Urocitellus parryii): implications for attenuation of disuse muscle atrophy
Source: Sci Rep. 2020 Jun 2;10:9010. doi: 10.1038/s41598-020-66030-9 (PMC7265340; doi:10.1038/s41598-020-66030-9)
Supplement: Supplementary file 1 — Supplementary Information. [file 41598_2020_66030_MOESM1_ESM.docx]

**Transcriptional changes in muscle of hibernating arctic ground squirrels (*Urocitellus parryii***)**: implications for attenuation of disuse muscle atrophy.**

**Anna V. Goropashnaya, Brian M. Barnes, Vadim B. Fedorov**

Institute of Arctic Biology, University of Alaska Fairbanks, Fairbanks, AK 99775-7000, USA

Contact Information: Vadim Fedorov, tel. +1 (907) 474 1533, e-mail: vfedorov@alaska.edu

**Table 1S** Differentially expressed genes in muscle of arctic ground squirrels. LT-late torpor, ET- entering torpor, SA – summer active control

| Gene ID | Name | Log₂ fold change | Fold change | P-value | FDR p-value |
| --- | --- | --- | --- | --- | --- |
| **LT vs. ET** |  |  |  |  |  |
| [101970831](https://www.ncbi.nlm.nih.gov/entrez/query.fcgi?db=gene&cmd=Retrieve&dopt=full_report&list_uids=101970831) | LOC101970831 | 5.908028 | 60.04734 | 2.83E-09 | 2.79E-06 |
| [101970551](https://www.ncbi.nlm.nih.gov/entrez/query.fcgi?db=gene&cmd=Retrieve&dopt=full_report&list_uids=101970551) | Tf | 5.607198 | 48.74554 | 2.09E-09 | 2.38E-06 |
| [101962170](https://www.ncbi.nlm.nih.gov/entrez/query.fcgi?db=gene&cmd=Retrieve&dopt=full_report&list_uids=101962170) | Hpx | 5.10801 | 34.4877 | 3E-08 | 2.01E-05 |
| [101975693](https://www.ncbi.nlm.nih.gov/entrez/query.fcgi?db=gene&cmd=Retrieve&dopt=full_report&list_uids=101975693) | Aldob | 4.754789 | 26.99815 | 2.03E-07 | 9.77E-05 |
| [101954486](https://www.ncbi.nlm.nih.gov/entrez/query.fcgi?db=gene&cmd=Retrieve&dopt=full_report&list_uids=101954486) | Itih4 | 4.486946 | 22.42361 | 4.71E-10 | 1.56E-06 |
| [101954502](https://www.ncbi.nlm.nih.gov/entrez/query.fcgi?db=gene&cmd=Retrieve&dopt=full_report&list_uids=101954502) | AKR1C3 | 4.479069 | 22.30151 | 1.72E-09 | 2.38E-06 |
| [101967403](https://www.ncbi.nlm.nih.gov/entrez/query.fcgi?db=gene&cmd=Retrieve&dopt=full_report&list_uids=101967403) | F12 | 4.395234 | 21.0425 | 8.76E-08 | 4.58E-05 |
| [101976500](https://www.ncbi.nlm.nih.gov/entrez/query.fcgi?db=gene&cmd=Retrieve&dopt=full_report&list_uids=101976500) | Hp | 4.369172 | 20.66579 | 1.33E-08 | 1E-05 |
| [101966057](https://www.ncbi.nlm.nih.gov/entrez/query.fcgi?db=gene&cmd=Retrieve&dopt=full_report&list_uids=101966057) | Rbp4 | 4.227535 | 18.73332 | 3.01E-09 | 2.79E-06 |
| [101959820](https://www.ncbi.nlm.nih.gov/entrez/query.fcgi?db=gene&cmd=Retrieve&dopt=full_report&list_uids=101959820) | Vtn | 3.815034 | 14.07472 | 4.23E-09 | 3.64E-06 |
| [101974890](https://www.ncbi.nlm.nih.gov/entrez/query.fcgi?db=gene&cmd=Retrieve&dopt=full_report&list_uids=101974890) | LOC101974890 | 3.775979 | 13.69881 | 4.18E-11 | 2.52E-07 |
| [101975231](https://www.ncbi.nlm.nih.gov/entrez/query.fcgi?db=gene&cmd=Retrieve&dopt=full_report&list_uids=101975231) | Serpinf2 | 3.629957 | 12.38015 | 2.5E-06 | 0.000669 |
| [101965332](https://www.ncbi.nlm.nih.gov/entrez/query.fcgi?db=gene&cmd=Retrieve&dopt=full_report&list_uids=101965332) | LOC101965332 | 3.623323 | 12.32335 | 3.53E-08 | 2.24E-05 |
| [101973490](https://www.ncbi.nlm.nih.gov/entrez/query.fcgi?db=gene&cmd=Retrieve&dopt=full_report&list_uids=101973490) | Abcb4 | 3.599352 | 12.12029 | 8.18E-08 | 4.48E-05 |
| [101961664](https://www.ncbi.nlm.nih.gov/entrez/query.fcgi?db=gene&cmd=Retrieve&dopt=full_report&list_uids=101961664) | Apoe | 3.525678 | 11.51688 | 6.26E-10 | 1.56E-06 |
| [101977232](https://www.ncbi.nlm.nih.gov/entrez/query.fcgi?db=gene&cmd=Retrieve&dopt=full_report&list_uids=101977232) | Cfb | 3.457403 | 10.98455 | 3.75E-06 | 0.000923 |
| [101963163](https://www.ncbi.nlm.nih.gov/entrez/query.fcgi?db=gene&cmd=Retrieve&dopt=full_report&list_uids=101963163) | Slc25a47 | 3.438253 | 10.8397 | 7.43E-06 | 0.001598 |
| [101965713](https://www.ncbi.nlm.nih.gov/entrez/query.fcgi?db=gene&cmd=Retrieve&dopt=full_report&list_uids=101965713) | Slc10a1 | 3.260261 | 9.581561 | 6.86E-07 | 0.000247 |
| [101967402](https://www.ncbi.nlm.nih.gov/entrez/query.fcgi?db=gene&cmd=Retrieve&dopt=full_report&list_uids=101967402) | LOC101967402 | 3.186133 | 9.101681 | 7.91E-08 | 4.48E-05 |
| [101973237](https://www.ncbi.nlm.nih.gov/entrez/query.fcgi?db=gene&cmd=Retrieve&dopt=full_report&list_uids=101973237) | Krt18 | 3.153335 | 8.8971 | 2.95E-07 | 0.000131 |
| [101975827](https://www.ncbi.nlm.nih.gov/entrez/query.fcgi?db=gene&cmd=Retrieve&dopt=full_report&list_uids=101975827) | C4a | 3.117905 | 8.681262 | 6.76E-07 | 0.000247 |
| [101967654](https://www.ncbi.nlm.nih.gov/entrez/query.fcgi?db=gene&cmd=Retrieve&dopt=full_report&list_uids=101967654) | Thrsp | 3.073655 | 8.419036 | 7.78E-10 | 1.56E-06 |
| [101968287](https://www.ncbi.nlm.nih.gov/entrez/query.fcgi?db=gene&cmd=Retrieve&dopt=full_report&list_uids=101968287) | Pck2 | 3.021804 | 8.121827 | 8.94E-07 | 0.000308 |
| [101964363](https://www.ncbi.nlm.nih.gov/entrez/query.fcgi?db=gene&cmd=Retrieve&dopt=full_report&list_uids=101964363) | Acat2 | 2.953941 | 7.748626 | 2.37E-07 | 0.00011 |
| [101959378](https://www.ncbi.nlm.nih.gov/entrez/query.fcgi?db=gene&cmd=Retrieve&dopt=full_report&list_uids=101959378) | LOC101959378 | 2.921393 | 7.575772 | 1.43E-05 | 0.002827 |
| [101973713](https://www.ncbi.nlm.nih.gov/entrez/query.fcgi?db=gene&cmd=Retrieve&dopt=full_report&list_uids=101973713) | LOC101973713 | 2.894371 | 7.435199 | 1.79E-05 | 0.003222 |
| [101962133](https://www.ncbi.nlm.nih.gov/entrez/query.fcgi?db=gene&cmd=Retrieve&dopt=full_report&list_uids=101962133) | Gls2 | 2.847102 | 7.195534 | 9.58E-10 | 1.65E-06 |
| [101955669](https://www.ncbi.nlm.nih.gov/entrez/query.fcgi?db=gene&cmd=Retrieve&dopt=full_report&list_uids=101955669) | LOC101955669 | 2.727034 | 6.620932 | 9.73E-06 | 0.002021 |
| [101971845](https://www.ncbi.nlm.nih.gov/entrez/query.fcgi?db=gene&cmd=Retrieve&dopt=full_report&list_uids=101971845) | Shroom1 | 2.725341 | 6.613166 | 2.16E-06 | 0.000605 |
| [101964310](https://www.ncbi.nlm.nih.gov/entrez/query.fcgi?db=gene&cmd=Retrieve&dopt=full_report&list_uids=101964310) | LOC101964310 | 2.720351 | 6.59033 | 9.71E-05 | 0.011928 |
| [101976320](https://www.ncbi.nlm.nih.gov/entrez/query.fcgi?db=gene&cmd=Retrieve&dopt=full_report&list_uids=101976320) | Kcnk3 | 2.684943 | 6.430553 | 5.31E-12 | 6.4E-08 |
| [101957914](https://www.ncbi.nlm.nih.gov/entrez/query.fcgi?db=gene&cmd=Retrieve&dopt=full_report&list_uids=101957914) | Iqgap2 | 2.674925 | 6.386054 | 1.04E-08 | 8.32E-06 |
| [101967464](https://www.ncbi.nlm.nih.gov/entrez/query.fcgi?db=gene&cmd=Retrieve&dopt=full_report&list_uids=101967464) | LOC101967464 | 2.627491 | 6.179505 | 1.42E-06 | 0.000451 |
| [101955757](https://www.ncbi.nlm.nih.gov/entrez/query.fcgi?db=gene&cmd=Retrieve&dopt=full_report&list_uids=101955757) | Ass1 | 2.625567 | 6.171268 | 2.18E-09 | 2.38E-06 |
| [101975126](https://www.ncbi.nlm.nih.gov/entrez/query.fcgi?db=gene&cmd=Retrieve&dopt=full_report&list_uids=101975126) | LOC101975126 | 2.619337 | 6.144677 | 2.48E-05 | 0.004153 |
| [101977160](https://www.ncbi.nlm.nih.gov/entrez/query.fcgi?db=gene&cmd=Retrieve&dopt=full_report&list_uids=101977160) | LOC101977160 | 2.587101 | 6.008902 | 0.000439 | 0.034979 |
| [101972974](https://www.ncbi.nlm.nih.gov/entrez/query.fcgi?db=gene&cmd=Retrieve&dopt=full_report&list_uids=101972974) | Mgst1 | 2.583088 | 5.99221 | 6.87E-10 | 1.56E-06 |
| [101959731](https://www.ncbi.nlm.nih.gov/entrez/query.fcgi?db=gene&cmd=Retrieve&dopt=full_report&list_uids=101959731) | C3 | 2.580832 | 5.982845 | 6.18E-06 | 0.001405 |
| [101971335](https://www.ncbi.nlm.nih.gov/entrez/query.fcgi?db=gene&cmd=Retrieve&dopt=full_report&list_uids=101971335) | Gpr37l1 | 2.577019 | 5.967053 | 5.65E-05 | 0.007917 |
| [101967759](https://www.ncbi.nlm.nih.gov/entrez/query.fcgi?db=gene&cmd=Retrieve&dopt=full_report&list_uids=101967759) | Galm | 2.576606 | 5.965346 | 4.51E-07 | 0.000181 |
| [101963153](https://www.ncbi.nlm.nih.gov/entrez/query.fcgi?db=gene&cmd=Retrieve&dopt=full_report&list_uids=101963153) | Cgn | 2.529217 | 5.772584 | 0.000168 | 0.018024 |
| [101966045](https://www.ncbi.nlm.nih.gov/entrez/query.fcgi?db=gene&cmd=Retrieve&dopt=full_report&list_uids=101966045) | LOC101966045 | 2.518375 | 5.729364 | 4.83E-05 | 0.007092 |
| [101973974](https://www.ncbi.nlm.nih.gov/entrez/query.fcgi?db=gene&cmd=Retrieve&dopt=full_report&list_uids=101973974) | F5 | 2.517527 | 5.725998 | 4.76E-05 | 0.00707 |
| [101955377](https://www.ncbi.nlm.nih.gov/entrez/query.fcgi?db=gene&cmd=Retrieve&dopt=full_report&list_uids=101955377) | Rgn | 2.501124 | 5.661261 | 6.01E-05 | 0.008221 |
| [101956370](https://www.ncbi.nlm.nih.gov/entrez/query.fcgi?db=gene&cmd=Retrieve&dopt=full_report&list_uids=101956370) | Pcsk9 | 2.449157 | 5.460968 | 0.000285 | 0.026019 |
| [101969148](https://www.ncbi.nlm.nih.gov/entrez/query.fcgi?db=gene&cmd=Retrieve&dopt=full_report&list_uids=101969148) | LOC101969148 | 2.428258 | 5.38243 | 2.16E-06 | 0.000605 |
| [101971739](https://www.ncbi.nlm.nih.gov/entrez/query.fcgi?db=gene&cmd=Retrieve&dopt=full_report&list_uids=101971739) | LOC101971739 | 2.380576 | 5.207446 | 2.02E-05 | 0.003481 |
| [101954307](https://www.ncbi.nlm.nih.gov/entrez/query.fcgi?db=gene&cmd=Retrieve&dopt=full_report&list_uids=101954307) | Gzmk | 2.37115 | 5.173534 | 0.000535 | 0.039984 |
| [101969717](https://www.ncbi.nlm.nih.gov/entrez/query.fcgi?db=gene&cmd=Retrieve&dopt=full_report&list_uids=101969717) | Mettl21c | 2.367488 | 5.160419 | 0.000638 | 0.044799 |
| [101971234](https://www.ncbi.nlm.nih.gov/entrez/query.fcgi?db=gene&cmd=Retrieve&dopt=full_report&list_uids=101971234) | Hsd11b1 | 2.289995 | 4.890546 | 1.52E-06 | 0.000469 |
| [101972052](https://www.ncbi.nlm.nih.gov/entrez/query.fcgi?db=gene&cmd=Retrieve&dopt=full_report&list_uids=101972052) | Vwa3b | 2.273742 | 4.835757 | 3.03E-06 | 0.000761 |
| [101974275](https://www.ncbi.nlm.nih.gov/entrez/query.fcgi?db=gene&cmd=Retrieve&dopt=full_report&list_uids=101974275) | Maob | 2.266278 | 4.810804 | 1.92E-09 | 2.38E-06 |
| [101957679](https://www.ncbi.nlm.nih.gov/entrez/query.fcgi?db=gene&cmd=Retrieve&dopt=full_report&list_uids=101957679) | Bok | 2.228328 | 4.685905 | 0.000418 | 0.034025 |
| [101970238](https://www.ncbi.nlm.nih.gov/entrez/query.fcgi?db=gene&cmd=Retrieve&dopt=full_report&list_uids=101970238) | Pygl | 2.223572 | 4.670485 | 1.04E-07 | 5.2E-05 |
| [101972738](https://www.ncbi.nlm.nih.gov/entrez/query.fcgi?db=gene&cmd=Retrieve&dopt=full_report&list_uids=101972738) | Anpep | 2.222342 | 4.666503 | 1.73E-05 | 0.003222 |
| [101960513](https://www.ncbi.nlm.nih.gov/entrez/query.fcgi?db=gene&cmd=Retrieve&dopt=full_report&list_uids=101960513) | Dpys | 2.18809 | 4.557018 | 1.91E-06 | 0.00056 |
| [101970926](https://www.ncbi.nlm.nih.gov/entrez/query.fcgi?db=gene&cmd=Retrieve&dopt=full_report&list_uids=101970926) | LOC101970926 | 2.129169 | 4.374656 | 4.96E-05 | 0.007197 |
| [101969252](https://www.ncbi.nlm.nih.gov/entrez/query.fcgi?db=gene&cmd=Retrieve&dopt=full_report&list_uids=101969252) | Fam107a | 2.048578 | 4.136979 | 2.76E-06 | 0.000724 |
| [101978344](https://www.ncbi.nlm.nih.gov/entrez/query.fcgi?db=gene&cmd=Retrieve&dopt=full_report&list_uids=101978344) | C2 | 2.039246 | 4.110305 | 1.71E-06 | 0.000514 |
| [101963701](https://www.ncbi.nlm.nih.gov/entrez/query.fcgi?db=gene&cmd=Retrieve&dopt=full_report&list_uids=101963701) | Prlr | 2.030009 | 4.084074 | 0.000109 | 0.013108 |
| [101965656](https://www.ncbi.nlm.nih.gov/entrez/query.fcgi?db=gene&cmd=Retrieve&dopt=full_report&list_uids=101965656) | Slc16a11 | 2.026074 | 4.072949 | 2.98E-06 | 0.000761 |
| [101976773](https://www.ncbi.nlm.nih.gov/entrez/query.fcgi?db=gene&cmd=Retrieve&dopt=full_report&list_uids=101976773) | Znf662 | 2.015635 | 4.043585 | 0.000597 | 0.043183 |
| [101957238](https://www.ncbi.nlm.nih.gov/entrez/query.fcgi?db=gene&cmd=Retrieve&dopt=full_report&list_uids=101957238) | Hoxd8 | 1.999233 | 3.997873 | 0.000257 | 0.024376 |
| [101975447](https://www.ncbi.nlm.nih.gov/entrez/query.fcgi?db=gene&cmd=Retrieve&dopt=full_report&list_uids=101975447) | C1r | 1.979914 | 3.944695 | 6.42E-06 | 0.001432 |
| [101961005](https://www.ncbi.nlm.nih.gov/entrez/query.fcgi?db=gene&cmd=Retrieve&dopt=full_report&list_uids=101961005) | Pros1 | 1.933534 | 3.819898 | 7.37E-05 | 0.009338 |
| [101967355](https://www.ncbi.nlm.nih.gov/entrez/query.fcgi?db=gene&cmd=Retrieve&dopt=full_report&list_uids=101967355) | Dhtkd1 | 1.908925 | 3.755293 | 2.55E-05 | 0.0042 |
| [101975661](https://www.ncbi.nlm.nih.gov/entrez/query.fcgi?db=gene&cmd=Retrieve&dopt=full_report&list_uids=101975661) | Nfil3 | 1.905825 | 3.747231 | 3.54E-07 | 0.000152 |
| [101967240](https://www.ncbi.nlm.nih.gov/entrez/query.fcgi?db=gene&cmd=Retrieve&dopt=full_report&list_uids=101967240) | Fah | 1.890774 | 3.708341 | 0.000305 | 0.026638 |
| [101969420](https://www.ncbi.nlm.nih.gov/entrez/query.fcgi?db=gene&cmd=Retrieve&dopt=full_report&list_uids=101969420) | Lpin2 | 1.879629 | 3.679803 | 0.000248 | 0.02405 |
| [101957178](https://www.ncbi.nlm.nih.gov/entrez/query.fcgi?db=gene&cmd=Retrieve&dopt=full_report&list_uids=101957178) | Man1a1 | 1.868831 | 3.652366 | 0.000286 | 0.026019 |
| [101967779](https://www.ncbi.nlm.nih.gov/entrez/query.fcgi?db=gene&cmd=Retrieve&dopt=full_report&list_uids=101967779) | Cebpa | 1.86018 | 3.630531 | 2E-05 | 0.003481 |
| [101975731](https://www.ncbi.nlm.nih.gov/entrez/query.fcgi?db=gene&cmd=Retrieve&dopt=full_report&list_uids=101975731) | C1s | 1.790702 | 3.459832 | 1.88E-05 | 0.003327 |
| [101973558](https://www.ncbi.nlm.nih.gov/entrez/query.fcgi?db=gene&cmd=Retrieve&dopt=full_report&list_uids=101973558) | Sfxn1 | 1.789786 | 3.457636 | 1.06E-05 | 0.002161 |
| [101975243](https://www.ncbi.nlm.nih.gov/entrez/query.fcgi?db=gene&cmd=Retrieve&dopt=full_report&list_uids=101975243) | Rab37 | 1.789421 | 3.456761 | 0.000542 | 0.040287 |
| [101969786](https://www.ncbi.nlm.nih.gov/entrez/query.fcgi?db=gene&cmd=Retrieve&dopt=full_report&list_uids=101969786) | Serping1 | 1.786449 | 3.449647 | 6.51E-05 | 0.00861 |
| [101957080](https://www.ncbi.nlm.nih.gov/entrez/query.fcgi?db=gene&cmd=Retrieve&dopt=full_report&list_uids=101957080) | Mettl7b | 1.769559 | 3.409497 | 2.13E-05 | 0.003611 |
| [101977214](https://www.ncbi.nlm.nih.gov/entrez/query.fcgi?db=gene&cmd=Retrieve&dopt=full_report&list_uids=101977214) | Pln | 1.75793 | 3.382125 | 0.000299 | 0.026479 |
| [101966179](https://www.ncbi.nlm.nih.gov/entrez/query.fcgi?db=gene&cmd=Retrieve&dopt=full_report&list_uids=101966179) | Sord | 1.743752 | 3.349051 | 0.000257 | 0.024376 |
| [101970643](https://www.ncbi.nlm.nih.gov/entrez/query.fcgi?db=gene&cmd=Retrieve&dopt=full_report&list_uids=101970643) | Otud1 | 1.735323 | 3.329539 | 4.13E-07 | 0.000171 |
| [101955844](https://www.ncbi.nlm.nih.gov/entrez/query.fcgi?db=gene&cmd=Retrieve&dopt=full_report&list_uids=101955844) | Pcsk6 | 1.69079 | 3.228334 | 0.00055 | 0.040654 |
| [101958148](https://www.ncbi.nlm.nih.gov/entrez/query.fcgi?db=gene&cmd=Retrieve&dopt=full_report&list_uids=101958148) | Ccbl2 | 1.68486 | 3.215092 | 2.96E-05 | 0.00476 |
| [101967031](https://www.ncbi.nlm.nih.gov/entrez/query.fcgi?db=gene&cmd=Retrieve&dopt=full_report&list_uids=101967031) | Ankrd2 | 1.653803 | 3.14662 | 0.000191 | 0.019518 |
| [101975580](https://www.ncbi.nlm.nih.gov/entrez/query.fcgi?db=gene&cmd=Retrieve&dopt=full_report&list_uids=101975580) | Igfbp4 | 1.600907 | 3.03334 | 6.48E-05 | 0.00861 |
| [101963574](https://www.ncbi.nlm.nih.gov/entrez/query.fcgi?db=gene&cmd=Retrieve&dopt=full_report&list_uids=101963574) | Apom | 1.591453 | 3.013526 | 0.000454 | 0.035766 |
| [101973579](https://www.ncbi.nlm.nih.gov/entrez/query.fcgi?db=gene&cmd=Retrieve&dopt=full_report&list_uids=101973579) | LOC101973579 | 1.591207 | 3.013014 | 0.000348 | 0.02931 |
| [101970563](https://www.ncbi.nlm.nih.gov/entrez/query.fcgi?db=gene&cmd=Retrieve&dopt=full_report&list_uids=101970563) | St6gal1 | 1.569587 | 2.968197 | 0.000169 | 0.01803 |
| [101972342](https://www.ncbi.nlm.nih.gov/entrez/query.fcgi?db=gene&cmd=Retrieve&dopt=full_report&list_uids=101972342) | Ier5 | 1.561959 | 2.952544 | 6.98E-07 | 0.000247 |
| [101955573](https://www.ncbi.nlm.nih.gov/entrez/query.fcgi?db=gene&cmd=Retrieve&dopt=full_report&list_uids=101955573) | Slco2b1 | 1.560616 | 2.949797 | 5.82E-05 | 0.008062 |
| [101963455](https://www.ncbi.nlm.nih.gov/entrez/query.fcgi?db=gene&cmd=Retrieve&dopt=full_report&list_uids=101963455) | Crtac1 | 1.556774 | 2.941952 | 0.000422 | 0.034037 |
| [101957018](https://www.ncbi.nlm.nih.gov/entrez/query.fcgi?db=gene&cmd=Retrieve&dopt=full_report&list_uids=101957018) | LOC101957018 | 1.510674 | 2.84943 | 0.000108 | 0.013108 |
| [101967724](https://www.ncbi.nlm.nih.gov/entrez/query.fcgi?db=gene&cmd=Retrieve&dopt=full_report&list_uids=101967724) | Lamc2 | 1.508124 | 2.8444 | 4.31E-05 | 0.006488 |
| [101966256](https://www.ncbi.nlm.nih.gov/entrez/query.fcgi?db=gene&cmd=Retrieve&dopt=full_report&list_uids=101966256) | Nipsnap1 | 1.505957 | 2.84013 | 2.66E-05 | 0.004322 |
| [101962454](https://www.ncbi.nlm.nih.gov/entrez/query.fcgi?db=gene&cmd=Retrieve&dopt=full_report&list_uids=101962454) | Fam129a | 1.476643 | 2.783003 | 4.25E-05 | 0.006472 |
| [101963150](https://www.ncbi.nlm.nih.gov/entrez/query.fcgi?db=gene&cmd=Retrieve&dopt=full_report&list_uids=101963150) | LOC101963150 | 1.473362 | 2.776682 | 0.000164 | 0.017841 |
| [101976389](https://www.ncbi.nlm.nih.gov/entrez/query.fcgi?db=gene&cmd=Retrieve&dopt=full_report&list_uids=101976389) | Lipe | 1.424837 | 2.684841 | 0.000226 | 0.022573 |
| [101971345](https://www.ncbi.nlm.nih.gov/entrez/query.fcgi?db=gene&cmd=Retrieve&dopt=full_report&list_uids=101971345) | Jchain | 1.414359 | 2.665414 | 0.000562 | 0.041261 |
| [101956908](https://www.ncbi.nlm.nih.gov/entrez/query.fcgi?db=gene&cmd=Retrieve&dopt=full_report&list_uids=101956908) | Vwa5a | 1.407275 | 2.652357 | 0.000233 | 0.022993 |
| [101960145](https://www.ncbi.nlm.nih.gov/entrez/query.fcgi?db=gene&cmd=Retrieve&dopt=full_report&list_uids=101960145) | LOC101960145 | 1.349211 | 2.547727 | 0.000257 | 0.024376 |
| [101968512](https://www.ncbi.nlm.nih.gov/entrez/query.fcgi?db=gene&cmd=Retrieve&dopt=full_report&list_uids=101968512) | Mfap4 | 1.34743 | 2.544584 | 0.000489 | 0.037762 |
| [101960438](https://www.ncbi.nlm.nih.gov/entrez/query.fcgi?db=gene&cmd=Retrieve&dopt=full_report&list_uids=101960438) | Psmb9 | 1.328683 | 2.511733 | 0.000462 | 0.036101 |
| [101960577](https://www.ncbi.nlm.nih.gov/entrez/query.fcgi?db=gene&cmd=Retrieve&dopt=full_report&list_uids=101960577) | Ephx1 | 1.297752 | 2.458455 | 0.000454 | 0.035766 |
| [101974299](https://www.ncbi.nlm.nih.gov/entrez/query.fcgi?db=gene&cmd=Retrieve&dopt=full_report&list_uids=101974299) | Hpn | 1.270286 | 2.412094 | 0.000364 | 0.03019 |
| [101975355](https://www.ncbi.nlm.nih.gov/entrez/query.fcgi?db=gene&cmd=Retrieve&dopt=full_report&list_uids=101975355) | Crim1 | 1.263307 | 2.400454 | 0.000131 | 0.01515 |
| [101956844](https://www.ncbi.nlm.nih.gov/entrez/query.fcgi?db=gene&cmd=Retrieve&dopt=full_report&list_uids=101956844) | LOC101956844 | 1.240734 | 2.363188 | 0.000292 | 0.026019 |
| [101977803](https://www.ncbi.nlm.nih.gov/entrez/query.fcgi?db=gene&cmd=Retrieve&dopt=full_report&list_uids=101977803) | Ablim1 | 1.21809 | 2.326385 | 0.000504 | 0.038187 |
| [101955831](https://www.ncbi.nlm.nih.gov/entrez/query.fcgi?db=gene&cmd=Retrieve&dopt=full_report&list_uids=101955831) | Hspa1b | 1.183688 | 2.271567 | 0.00014 | 0.015993 |
| [101958861](https://www.ncbi.nlm.nih.gov/entrez/query.fcgi?db=gene&cmd=Retrieve&dopt=full_report&list_uids=101958861) | Plxdc2 | 1.165067 | 2.242437 | 0.000128 | 0.014989 |
| [101965063](https://www.ncbi.nlm.nih.gov/entrez/query.fcgi?db=gene&cmd=Retrieve&dopt=full_report&list_uids=101965063) | Aldh7a1 | 1.161018 | 2.236152 | 6.8E-05 | 0.008799 |
| [101964485](https://www.ncbi.nlm.nih.gov/entrez/query.fcgi?db=gene&cmd=Retrieve&dopt=full_report&list_uids=101964485) | LOC101964485 | 1.100473 | 2.14425 | 0.000113 | 0.013429 |
| [101963185](https://www.ncbi.nlm.nih.gov/entrez/query.fcgi?db=gene&cmd=Retrieve&dopt=full_report&list_uids=101963185) | Ankrd10 | 1.098328 | 2.141064 | 0.00029 | 0.026019 |
| [101963422](https://www.ncbi.nlm.nih.gov/entrez/query.fcgi?db=gene&cmd=Retrieve&dopt=full_report&list_uids=101963422) | Cpe | 1.072989 | 2.103788 | 0.000322 | 0.027721 |
| [101970827](https://www.ncbi.nlm.nih.gov/entrez/query.fcgi?db=gene&cmd=Retrieve&dopt=full_report&list_uids=101970827) | Mss51 | 1.051946 | 2.073324 | 0.000277 | 0.026017 |
| [101971235](https://www.ncbi.nlm.nih.gov/entrez/query.fcgi?db=gene&cmd=Retrieve&dopt=full_report&list_uids=101971235) | Ccdc92 | 1.045726 | 2.064405 | 7.28E-05 | 0.009332 |
| [101955225](https://www.ncbi.nlm.nih.gov/entrez/query.fcgi?db=gene&cmd=Retrieve&dopt=full_report&list_uids=101955225) | Suox | 1.044437 | 2.062561 | 0.000531 | 0.039933 |
| [101966036](https://www.ncbi.nlm.nih.gov/entrez/query.fcgi?db=gene&cmd=Retrieve&dopt=full_report&list_uids=101966036) | Smim13 | 0.959265 | 1.94432 | 0.000117 | 0.013863 |
| [101956336](https://www.ncbi.nlm.nih.gov/entrez/query.fcgi?db=gene&cmd=Retrieve&dopt=full_report&list_uids=101956336) | Slc20a2 | -0.88769 | -1.85022 | 0.000494 | 0.03787 |
| [101966902](https://www.ncbi.nlm.nih.gov/entrez/query.fcgi?db=gene&cmd=Retrieve&dopt=full_report&list_uids=101966902) | Rnf144a | -0.94025 | -1.91886 | 0.00064 | 0.044799 |
| [101972961](https://www.ncbi.nlm.nih.gov/entrez/query.fcgi?db=gene&cmd=Retrieve&dopt=full_report&list_uids=101972961) | Phkg1 | -0.98379 | -1.97766 | 0.000288 | 0.026019 |
| [101965344](https://www.ncbi.nlm.nih.gov/entrez/query.fcgi?db=gene&cmd=Retrieve&dopt=full_report&list_uids=101965344) | Per2 | -1.08868 | -2.12679 | 0.000386 | 0.031874 |
| [101976136](https://www.ncbi.nlm.nih.gov/entrez/query.fcgi?db=gene&cmd=Retrieve&dopt=full_report&list_uids=101976136) | Esr1 | -1.08892 | -2.12715 | 0.000248 | 0.02405 |
| [101976003](https://www.ncbi.nlm.nih.gov/entrez/query.fcgi?db=gene&cmd=Retrieve&dopt=full_report&list_uids=101976003) | Mlec | -1.09192 | -2.13158 | 0.000424 | 0.034037 |
| [101957943](https://www.ncbi.nlm.nih.gov/entrez/query.fcgi?db=gene&cmd=Retrieve&dopt=full_report&list_uids=101957943) | Trib1 | -1.12284 | -2.17776 | 0.000188 | 0.019394 |
| [101978630](https://www.ncbi.nlm.nih.gov/entrez/query.fcgi?db=gene&cmd=Retrieve&dopt=full_report&list_uids=101978630) | Cep85 | -1.12615 | -2.18275 | 5.25E-05 | 0.007527 |
| [101955564](https://www.ncbi.nlm.nih.gov/entrez/query.fcgi?db=gene&cmd=Retrieve&dopt=full_report&list_uids=101955564) | Osbpl7 | -1.13538 | -2.19676 | 0.000504 | 0.038187 |
| [101973609](https://www.ncbi.nlm.nih.gov/entrez/query.fcgi?db=gene&cmd=Retrieve&dopt=full_report&list_uids=101973609) | Fam71e1 | -1.18191 | -2.26877 | 0.000617 | 0.043957 |
| [101958434](https://www.ncbi.nlm.nih.gov/entrez/query.fcgi?db=gene&cmd=Retrieve&dopt=full_report&list_uids=101958434) | Gadl1 | -1.19453 | -2.28871 | 0.000602 | 0.043183 |
| [101963833](https://www.ncbi.nlm.nih.gov/entrez/query.fcgi?db=gene&cmd=Retrieve&dopt=full_report&list_uids=101963833) | Rab3a | -1.20529 | -2.30583 | 0.000149 | 0.016775 |
| [101961480](https://www.ncbi.nlm.nih.gov/entrez/query.fcgi?db=gene&cmd=Retrieve&dopt=full_report&list_uids=101961480) | Sh2d7 | -1.23031 | -2.34618 | 3.69E-05 | 0.005764 |
| [101968875](https://www.ncbi.nlm.nih.gov/entrez/query.fcgi?db=gene&cmd=Retrieve&dopt=full_report&list_uids=101968875) | Phka1 | -1.24603 | -2.37188 | 0.000209 | 0.021184 |
| [101969741](https://www.ncbi.nlm.nih.gov/entrez/query.fcgi?db=gene&cmd=Retrieve&dopt=full_report&list_uids=101969741) | Mmd | -1.24699 | -2.37347 | 0.000333 | 0.028473 |
| [101959021](https://www.ncbi.nlm.nih.gov/entrez/query.fcgi?db=gene&cmd=Retrieve&dopt=full_report&list_uids=101959021) | Ttc9 | -1.25042 | -2.37911 | 0.000305 | 0.026638 |
| [101977713](https://www.ncbi.nlm.nih.gov/entrez/query.fcgi?db=gene&cmd=Retrieve&dopt=full_report&list_uids=101977713) | Mybpc2 | -1.2963 | -2.45598 | 0.0007 | 0.048741 |
| [101959076](https://www.ncbi.nlm.nih.gov/entrez/query.fcgi?db=gene&cmd=Retrieve&dopt=full_report&list_uids=101959076) | Tnik | -1.29642 | -2.45618 | 0.000279 | 0.026017 |
| [101969965](https://www.ncbi.nlm.nih.gov/entrez/query.fcgi?db=gene&cmd=Retrieve&dopt=full_report&list_uids=101969965) | Col6a3 | -1.30156 | -2.46496 | 0.000635 | 0.044799 |
| [101964840](https://www.ncbi.nlm.nih.gov/entrez/query.fcgi?db=gene&cmd=Retrieve&dopt=full_report&list_uids=101964840) | Tmem2 | -1.30705 | -2.47435 | 6.5E-05 | 0.00861 |
| [101965957](https://www.ncbi.nlm.nih.gov/entrez/query.fcgi?db=gene&cmd=Retrieve&dopt=full_report&list_uids=101965957) | LOC101965957 | -1.35641 | -2.56047 | 3.86E-05 | 0.005964 |
| [101958711](https://www.ncbi.nlm.nih.gov/entrez/query.fcgi?db=gene&cmd=Retrieve&dopt=full_report&list_uids=101958711) | LOC101958711 | -1.36348 | -2.57305 | 6.73E-05 | 0.008799 |
| [101971147](https://www.ncbi.nlm.nih.gov/entrez/query.fcgi?db=gene&cmd=Retrieve&dopt=full_report&list_uids=101971147) | Kcnj2 | -1.37217 | -2.58859 | 5.42E-06 | 0.001255 |
| [101957471](https://www.ncbi.nlm.nih.gov/entrez/query.fcgi?db=gene&cmd=Retrieve&dopt=full_report&list_uids=101957471) | Nedd1 | -1.37456 | -2.59288 | 4.31E-06 | 0.001019 |
| [101972762](https://www.ncbi.nlm.nih.gov/entrez/query.fcgi?db=gene&cmd=Retrieve&dopt=full_report&list_uids=101972762) | Fam57b | -1.37489 | -2.59347 | 0.000164 | 0.017841 |
| [101960294](https://www.ncbi.nlm.nih.gov/entrez/query.fcgi?db=gene&cmd=Retrieve&dopt=full_report&list_uids=101960294) | Greb1l | -1.37616 | -2.59576 | 0.00015 | 0.016775 |
| [101955894](https://www.ncbi.nlm.nih.gov/entrez/query.fcgi?db=gene&cmd=Retrieve&dopt=full_report&list_uids=101955894) | Mafb | -1.38511 | -2.61192 | 1.74E-05 | 0.003222 |
| [101972179](https://www.ncbi.nlm.nih.gov/entrez/query.fcgi?db=gene&cmd=Retrieve&dopt=full_report&list_uids=101972179) | Pgpep1l | -1.39066 | -2.62198 | 0.000322 | 0.027721 |
| [101957868](https://www.ncbi.nlm.nih.gov/entrez/query.fcgi?db=gene&cmd=Retrieve&dopt=full_report&list_uids=101957868) | Tet2 | -1.40189 | -2.64247 | 0.000227 | 0.022573 |
| [101969535](https://www.ncbi.nlm.nih.gov/entrez/query.fcgi?db=gene&cmd=Retrieve&dopt=full_report&list_uids=101969535) | Kcnq4 | -1.4238 | -2.6829 | 7.88E-05 | 0.00989 |
| [101956119](https://www.ncbi.nlm.nih.gov/entrez/query.fcgi?db=gene&cmd=Retrieve&dopt=full_report&list_uids=101956119) | Atxn7l2 | -1.43178 | -2.69779 | 0.000345 | 0.029228 |
| [101959178](https://www.ncbi.nlm.nih.gov/entrez/query.fcgi?db=gene&cmd=Retrieve&dopt=full_report&list_uids=101959178) | Dyrk1b | -1.43748 | -2.70848 | 1.02E-06 | 0.000342 |
| [101958931](https://www.ncbi.nlm.nih.gov/entrez/query.fcgi?db=gene&cmd=Retrieve&dopt=full_report&list_uids=101958931) | Dnmt3a | -1.44151 | -2.71604 | 8.85E-05 | 0.010985 |
| [101960116](https://www.ncbi.nlm.nih.gov/entrez/query.fcgi?db=gene&cmd=Retrieve&dopt=full_report&list_uids=101960116) | Igfbp5 | -1.54112 | -2.91019 | 1.57E-05 | 0.003053 |
| [101962730](https://www.ncbi.nlm.nih.gov/entrez/query.fcgi?db=gene&cmd=Retrieve&dopt=full_report&list_uids=101962730) | LOC101962730 | -1.5801 | -2.9899 | 0.000577 | 0.042086 |
| [101973643](https://www.ncbi.nlm.nih.gov/entrez/query.fcgi?db=gene&cmd=Retrieve&dopt=full_report&list_uids=101973643) | Hr | -1.6295 | -3.09406 | 0.00039 | 0.031928 |
| [101956607](https://www.ncbi.nlm.nih.gov/entrez/query.fcgi?db=gene&cmd=Retrieve&dopt=full_report&list_uids=101956607) | LOC101956607 | -1.65152 | -3.14164 | 0.000183 | 0.019019 |
| [101971458](https://www.ncbi.nlm.nih.gov/entrez/query.fcgi?db=gene&cmd=Retrieve&dopt=full_report&list_uids=101971458) | Zap70 | -1.69494 | -3.23763 | 0.000466 | 0.036203 |
| [101956240](https://www.ncbi.nlm.nih.gov/entrez/query.fcgi?db=gene&cmd=Retrieve&dopt=full_report&list_uids=101956240) | Cecr2 | -1.7008 | -3.25082 | 1.72E-05 | 0.003222 |
| [101977451](https://www.ncbi.nlm.nih.gov/entrez/query.fcgi?db=gene&cmd=Retrieve&dopt=full_report&list_uids=101977451) | Cradd | -1.71826 | -3.29039 | 3.91E-06 | 0.000942 |
| [101966425](https://www.ncbi.nlm.nih.gov/entrez/query.fcgi?db=gene&cmd=Retrieve&dopt=full_report&list_uids=101966425) | Myod1 | -1.75244 | -3.36928 | 5.38E-05 | 0.007624 |
| [101972189](https://www.ncbi.nlm.nih.gov/entrez/query.fcgi?db=gene&cmd=Retrieve&dopt=full_report&list_uids=101972189) | LOC101972189 | -1.7851 | -3.44643 | 0.000601 | 0.043183 |
| [101956216](https://www.ncbi.nlm.nih.gov/entrez/query.fcgi?db=gene&cmd=Retrieve&dopt=full_report&list_uids=101956216) | Nrg4 | -1.79227 | -3.4636 | 0.000171 | 0.018031 |
| [101964776](https://www.ncbi.nlm.nih.gov/entrez/query.fcgi?db=gene&cmd=Retrieve&dopt=full_report&list_uids=101964776) | Cdh15 | -1.89158 | -3.71042 | 6.56E-08 | 3.95E-05 |
| [101966164](https://www.ncbi.nlm.nih.gov/entrez/query.fcgi?db=gene&cmd=Retrieve&dopt=full_report&list_uids=101966164) | Rgs2 | -1.9639 | -3.90114 | 8E-06 | 0.00169 |
| [101959177](https://www.ncbi.nlm.nih.gov/entrez/query.fcgi?db=gene&cmd=Retrieve&dopt=full_report&list_uids=101959177) | LOC101959177 | -1.96418 | -3.9019 | 0.000352 | 0.029411 |
| [101969435](https://www.ncbi.nlm.nih.gov/entrez/query.fcgi?db=gene&cmd=Retrieve&dopt=full_report&list_uids=101969435) | Apln | -1.97027 | -3.9184 | 1.79E-05 | 0.003222 |
| [101976785](https://www.ncbi.nlm.nih.gov/entrez/query.fcgi?db=gene&cmd=Retrieve&dopt=full_report&list_uids=101976785) | LOC101976785 | -1.98323 | -3.95378 | 5.08E-07 | 0.000197 |
| [101962985](https://www.ncbi.nlm.nih.gov/entrez/query.fcgi?db=gene&cmd=Retrieve&dopt=full_report&list_uids=101962985) | Adamts9 | -1.997 | -3.99169 | 0.000291 | 0.026019 |
| [101973719](https://www.ncbi.nlm.nih.gov/entrez/query.fcgi?db=gene&cmd=Retrieve&dopt=full_report&list_uids=101973719) | Gad1 | -2.04535 | -4.12773 | 0.000182 | 0.019007 |
| [101954822](https://www.ncbi.nlm.nih.gov/entrez/query.fcgi?db=gene&cmd=Retrieve&dopt=full_report&list_uids=101954822) | Col4a4 | -2.12933 | -4.37514 | 0.000157 | 0.017342 |
| [101967668](https://www.ncbi.nlm.nih.gov/entrez/query.fcgi?db=gene&cmd=Retrieve&dopt=full_report&list_uids=101967668) | Cntfr | -2.17699 | -4.52208 | 2.39E-06 | 0.000654 |
| [101955667](https://www.ncbi.nlm.nih.gov/entrez/query.fcgi?db=gene&cmd=Retrieve&dopt=full_report&list_uids=101955667) | Capn6 | -2.25318 | -4.76732 | 7.12E-06 | 0.00156 |
| [101970565](https://www.ncbi.nlm.nih.gov/entrez/query.fcgi?db=gene&cmd=Retrieve&dopt=full_report&list_uids=101970565) | Nmrk2 | -2.25598 | -4.77659 | 0.000141 | 0.015993 |
| [101969612](https://www.ncbi.nlm.nih.gov/entrez/query.fcgi?db=gene&cmd=Retrieve&dopt=full_report&list_uids=101969612) | Rnf207 | -2.32473 | -5.00971 | 3.49E-05 | 0.005529 |
| [101956757](https://www.ncbi.nlm.nih.gov/entrez/query.fcgi?db=gene&cmd=Retrieve&dopt=full_report&list_uids=101956757) | Nrep | -2.32625 | -5.01499 | 2.7E-08 | 1.91E-05 |
| [101967109](https://www.ncbi.nlm.nih.gov/entrez/query.fcgi?db=gene&cmd=Retrieve&dopt=full_report&list_uids=101967109) | Traf5 | -2.36017 | -5.1343 | 1.36E-05 | 0.002732 |
| [101967703](https://www.ncbi.nlm.nih.gov/entrez/query.fcgi?db=gene&cmd=Retrieve&dopt=full_report&list_uids=101967703) | LOC101967703 | -2.56588 | -5.92117 | 1.26E-06 | 0.000411 |
| **ET vs. SA** |  |  |  |  |  |
| [101965561](https://www.ncbi.nlm.nih.gov/entrez/query.fcgi?db=gene&cmd=Retrieve&dopt=full_report&list_uids=101965561) | Plk5 | 4.351349 | 20.41205 | 1.09E-12 | 8.16E-10 |
| [101975693](https://www.ncbi.nlm.nih.gov/entrez/query.fcgi?db=gene&cmd=Retrieve&dopt=full_report&list_uids=101975693) | Aldob | 3.353615 | 10.22207 | 5.16E-05 | 0.003542 |
| [101963917](https://www.ncbi.nlm.nih.gov/entrez/query.fcgi?db=gene&cmd=Retrieve&dopt=full_report&list_uids=101963917) | Paqr9 | 3.286187 | 9.755303 | 5.88E-15 | 6.4E-12 |
| [101963149](https://www.ncbi.nlm.nih.gov/entrez/query.fcgi?db=gene&cmd=Retrieve&dopt=full_report&list_uids=101963149) | Igfn1 | 3.122082 | 8.706433 | 1.65E-06 | 0.000232 |
| [101974020](https://www.ncbi.nlm.nih.gov/entrez/query.fcgi?db=gene&cmd=Retrieve&dopt=full_report&list_uids=101974020) | Myh3 | 2.814455 | 7.034533 | 0.000321 | 0.014744 |
| [101966490](https://www.ncbi.nlm.nih.gov/entrez/query.fcgi?db=gene&cmd=Retrieve&dopt=full_report&list_uids=101966490) | Myo15a | 2.781076 | 6.873647 | 1.15E-05 | 0.001103 |
| [101954822](https://www.ncbi.nlm.nih.gov/entrez/query.fcgi?db=gene&cmd=Retrieve&dopt=full_report&list_uids=101954822) | Col4a4 | 2.546745 | 5.843144 | 4.23E-05 | 0.003089 |
| [101974285](https://www.ncbi.nlm.nih.gov/entrez/query.fcgi?db=gene&cmd=Retrieve&dopt=full_report&list_uids=101974285) | Ky | 2.423122 | 5.363304 | 7.69E-12 | 4.84E-09 |
| [101972730](https://www.ncbi.nlm.nih.gov/entrez/query.fcgi?db=gene&cmd=Retrieve&dopt=full_report&list_uids=101972730) | Ncapg | 2.380197 | 5.20608 | 2.39E-05 | 0.00196 |
| [101972152](https://www.ncbi.nlm.nih.gov/entrez/query.fcgi?db=gene&cmd=Retrieve&dopt=full_report&list_uids=101972152) | LOC101972152 | 2.372608 | 5.178764 | 6.77E-05 | 0.004422 |
| [101969233](https://www.ncbi.nlm.nih.gov/entrez/query.fcgi?db=gene&cmd=Retrieve&dopt=full_report&list_uids=101969233) | Fbp2 | 2.329797 | 5.027347 | 1.24E-06 | 0.000186 |
| [101973305](https://www.ncbi.nlm.nih.gov/entrez/query.fcgi?db=gene&cmd=Retrieve&dopt=full_report&list_uids=101973305) | Fam184b | 2.298628 | 4.919896 | 1.17E-07 | 2.25E-05 |
| [101962234](https://www.ncbi.nlm.nih.gov/entrez/query.fcgi?db=gene&cmd=Retrieve&dopt=full_report&list_uids=101962234) | Colq | 2.283165 | 4.867448 | 2.31E-06 | 0.000301 |
| [101969793](https://www.ncbi.nlm.nih.gov/entrez/query.fcgi?db=gene&cmd=Retrieve&dopt=full_report&list_uids=101969793) | Rhbdf2 | 2.20435 | 4.608669 | 4.94E-06 | 0.000574 |
| [101956607](https://www.ncbi.nlm.nih.gov/entrez/query.fcgi?db=gene&cmd=Retrieve&dopt=full_report&list_uids=101956607) | LOC101956607 | 2.147522 | 4.43066 | 1.99E-06 | 0.000272 |
| [101968473](https://www.ncbi.nlm.nih.gov/entrez/query.fcgi?db=gene&cmd=Retrieve&dopt=full_report&list_uids=101968473) | B3galt1 | 2.088001 | 4.251585 | 0.000224 | 0.011789 |
| [101975199](https://www.ncbi.nlm.nih.gov/entrez/query.fcgi?db=gene&cmd=Retrieve&dopt=full_report&list_uids=101975199) | Fam179a | 2.062716 | 4.177721 | 0.000312 | 0.014677 |
| [101961105](https://www.ncbi.nlm.nih.gov/entrez/query.fcgi?db=gene&cmd=Retrieve&dopt=full_report&list_uids=101961105) | Ncaph | 2.05592 | 4.158088 | 2.05E-05 | 0.001722 |
| [101971785](https://www.ncbi.nlm.nih.gov/entrez/query.fcgi?db=gene&cmd=Retrieve&dopt=full_report&list_uids=101971785) | Relt | 2.047585 | 4.134134 | 4.37E-06 | 0.000516 |
| [101969535](https://www.ncbi.nlm.nih.gov/entrez/query.fcgi?db=gene&cmd=Retrieve&dopt=full_report&list_uids=101969535) | Kcnq4 | 2.012277 | 4.034185 | 2.18E-06 | 0.000287 |
| [101974231](https://www.ncbi.nlm.nih.gov/entrez/query.fcgi?db=gene&cmd=Retrieve&dopt=full_report&list_uids=101974231) | Rdh5 | 2.007744 | 4.021528 | 0.000992 | 0.035065 |
| [106145258](https://www.ncbi.nlm.nih.gov/entrez/query.fcgi?db=gene&cmd=Retrieve&dopt=full_report&list_uids=106145258) | LOC106145258 | 1.997713 | 3.993663 | 0.001409 | 0.045598 |
| [101960769](https://www.ncbi.nlm.nih.gov/entrez/query.fcgi?db=gene&cmd=Retrieve&dopt=full_report&list_uids=101960769) | Tiam1 | 1.989049 | 3.969752 | 0.000107 | 0.006411 |
| [101955894](https://www.ncbi.nlm.nih.gov/entrez/query.fcgi?db=gene&cmd=Retrieve&dopt=full_report&list_uids=101955894) | Mafb | 1.968881 | 3.914642 | 7.44E-09 | 2.12E-06 |
| [101976096](https://www.ncbi.nlm.nih.gov/entrez/query.fcgi?db=gene&cmd=Retrieve&dopt=full_report&list_uids=101976096) | Lrrc26 | 1.961644 | 3.895055 | 0.000935 | 0.033387 |
| [101961125](https://www.ncbi.nlm.nih.gov/entrez/query.fcgi?db=gene&cmd=Retrieve&dopt=full_report&list_uids=101961125) | Klhl33 | 1.949314 | 3.861908 | 9.62E-08 | 1.92E-05 |
| [101955436](https://www.ncbi.nlm.nih.gov/entrez/query.fcgi?db=gene&cmd=Retrieve&dopt=full_report&list_uids=101955436) | Pla2g4b | 1.9317 | 3.815044 | 0.000161 | 0.008969 |
| [101974217](https://www.ncbi.nlm.nih.gov/entrez/query.fcgi?db=gene&cmd=Retrieve&dopt=full_report&list_uids=101974217) | Zfr2 | 1.918649 | 3.780687 | 0.000274 | 0.013284 |
| [101967535](https://www.ncbi.nlm.nih.gov/entrez/query.fcgi?db=gene&cmd=Retrieve&dopt=full_report&list_uids=101967535) | Emb | 1.913357 | 3.766846 | 1E-04 | 0.006038 |
| [101956037](https://www.ncbi.nlm.nih.gov/entrez/query.fcgi?db=gene&cmd=Retrieve&dopt=full_report&list_uids=101956037) | Myl5 | 1.911946 | 3.763163 | 3.63E-05 | 0.002762 |
| [101960116](https://www.ncbi.nlm.nih.gov/entrez/query.fcgi?db=gene&cmd=Retrieve&dopt=full_report&list_uids=101960116) | Igfbp5 | 1.910119 | 3.7584 | 1.73E-08 | 4.8E-06 |
| [101967531](https://www.ncbi.nlm.nih.gov/entrez/query.fcgi?db=gene&cmd=Retrieve&dopt=full_report&list_uids=101967531) | Tp73 | 1.895252 | 3.71987 | 0.000649 | 0.025422 |
| [101969707](https://www.ncbi.nlm.nih.gov/entrez/query.fcgi?db=gene&cmd=Retrieve&dopt=full_report&list_uids=101969707) | Rassf4 | 1.89107 | 3.709102 | 3.92E-09 | 1.28E-06 |
| [101973719](https://www.ncbi.nlm.nih.gov/entrez/query.fcgi?db=gene&cmd=Retrieve&dopt=full_report&list_uids=101973719) | Gad1 | 1.886677 | 3.697824 | 0.000266 | 0.013095 |
| [101954306](https://www.ncbi.nlm.nih.gov/entrez/query.fcgi?db=gene&cmd=Retrieve&dopt=full_report&list_uids=101954306) | Plk2 | 1.876056 | 3.670703 | 1.79E-08 | 4.87E-06 |
| [101977189](https://www.ncbi.nlm.nih.gov/entrez/query.fcgi?db=gene&cmd=Retrieve&dopt=full_report&list_uids=101977189) | Ca2 | 1.868663 | 3.651939 | 7.63E-08 | 1.6E-05 |
| [101954279](https://www.ncbi.nlm.nih.gov/entrez/query.fcgi?db=gene&cmd=Retrieve&dopt=full_report&list_uids=101954279) | Dact3 | 1.864138 | 3.640503 | 1.59E-05 | 0.001409 |
| [101977181](https://www.ncbi.nlm.nih.gov/entrez/query.fcgi?db=gene&cmd=Retrieve&dopt=full_report&list_uids=101977181) | Cyfip2 | 1.850424 | 3.606063 | 0.000252 | 0.012587 |
| [101968435](https://www.ncbi.nlm.nih.gov/entrez/query.fcgi?db=gene&cmd=Retrieve&dopt=full_report&list_uids=101968435) | Abcg4 | 1.843879 | 3.58974 | 0.001083 | 0.037213 |
| [101954640](https://www.ncbi.nlm.nih.gov/entrez/query.fcgi?db=gene&cmd=Retrieve&dopt=full_report&list_uids=101954640) | Chrnd | 1.82587 | 3.545207 | 4.46E-05 | 0.003173 |
| [101961960](https://www.ncbi.nlm.nih.gov/entrez/query.fcgi?db=gene&cmd=Retrieve&dopt=full_report&list_uids=101961960) | Esm1 | 1.820602 | 3.532286 | 0.000371 | 0.016425 |
| [101954817](https://www.ncbi.nlm.nih.gov/entrez/query.fcgi?db=gene&cmd=Retrieve&dopt=full_report&list_uids=101954817) | LOC101954817 | 1.81847 | 3.52707 | 0.000607 | 0.023915 |
| [101973086](https://www.ncbi.nlm.nih.gov/entrez/query.fcgi?db=gene&cmd=Retrieve&dopt=full_report&list_uids=101973086) | Taf1a | 1.787785 | 3.452844 | 0.000158 | 0.008902 |
| [101963835](https://www.ncbi.nlm.nih.gov/entrez/query.fcgi?db=gene&cmd=Retrieve&dopt=full_report&list_uids=101963835) | Cpt1a | 1.782791 | 3.440912 | 2.55E-06 | 0.000321 |
| [101959551](https://www.ncbi.nlm.nih.gov/entrez/query.fcgi?db=gene&cmd=Retrieve&dopt=full_report&list_uids=101959551) | Ncam1 | 1.779213 | 3.432389 | 2E-06 | 0.000272 |
| [101963241](https://www.ncbi.nlm.nih.gov/entrez/query.fcgi?db=gene&cmd=Retrieve&dopt=full_report&list_uids=101963241) | Meox1 | 1.771916 | 3.415073 | 0.000455 | 0.01911 |
| [101957992](https://www.ncbi.nlm.nih.gov/entrez/query.fcgi?db=gene&cmd=Retrieve&dopt=full_report&list_uids=101957992) | Plekhh1 | 1.764816 | 3.398307 | 0.000263 | 0.013028 |
| [101969691](https://www.ncbi.nlm.nih.gov/entrez/query.fcgi?db=gene&cmd=Retrieve&dopt=full_report&list_uids=101969691) | Tnfaip3 | 1.759515 | 3.385843 | 0.000134 | 0.007743 |
| [101972268](https://www.ncbi.nlm.nih.gov/entrez/query.fcgi?db=gene&cmd=Retrieve&dopt=full_report&list_uids=101972268) | Mettl11b | 1.748655 | 3.36045 | 5.9E-05 | 0.003919 |
| [101975511](https://www.ncbi.nlm.nih.gov/entrez/query.fcgi?db=gene&cmd=Retrieve&dopt=full_report&list_uids=101975511) | Bhlhe41 | 1.716934 | 3.28737 | 2.06E-06 | 0.000276 |
| [101976442](https://www.ncbi.nlm.nih.gov/entrez/query.fcgi?db=gene&cmd=Retrieve&dopt=full_report&list_uids=101976442) | Frzb | 1.715108 | 3.283213 | 8.94E-05 | 0.005629 |
| [101969741](https://www.ncbi.nlm.nih.gov/entrez/query.fcgi?db=gene&cmd=Retrieve&dopt=full_report&list_uids=101969741) | Mmd | 1.698551 | 3.245748 | 2.08E-06 | 0.000276 |
| [101978550](https://www.ncbi.nlm.nih.gov/entrez/query.fcgi?db=gene&cmd=Retrieve&dopt=full_report&list_uids=101978550) | Cpeb2 | 1.691127 | 3.229088 | 8.78E-08 | 1.78E-05 |
| [101958666](https://www.ncbi.nlm.nih.gov/entrez/query.fcgi?db=gene&cmd=Retrieve&dopt=full_report&list_uids=101958666) | Pcdh12 | 1.667154 | 3.175874 | 4.3E-06 | 0.000514 |
| [101963276](https://www.ncbi.nlm.nih.gov/entrez/query.fcgi?db=gene&cmd=Retrieve&dopt=full_report&list_uids=101963276) | Rhov | 1.659747 | 3.159612 | 0.001319 | 0.043099 |
| [101970969](https://www.ncbi.nlm.nih.gov/entrez/query.fcgi?db=gene&cmd=Retrieve&dopt=full_report&list_uids=101970969) | Fras1 | 1.648508 | 3.135093 | 0.000437 | 0.018519 |
| [101956444](https://www.ncbi.nlm.nih.gov/entrez/query.fcgi?db=gene&cmd=Retrieve&dopt=full_report&list_uids=101956444) | Hlcs | 1.641213 | 3.11928 | 2.49E-05 | 0.002008 |
| [101977465](https://www.ncbi.nlm.nih.gov/entrez/query.fcgi?db=gene&cmd=Retrieve&dopt=full_report&list_uids=101977465) | Hdac9 | 1.6385 | 3.113419 | 1.84E-05 | 0.001559 |
| [101967584](https://www.ncbi.nlm.nih.gov/entrez/query.fcgi?db=gene&cmd=Retrieve&dopt=full_report&list_uids=101967584) | LOC101967584 | 1.624168 | 3.082644 | 2.51E-06 | 0.000319 |
| [101975773](https://www.ncbi.nlm.nih.gov/entrez/query.fcgi?db=gene&cmd=Retrieve&dopt=full_report&list_uids=101975773) | Dach1 | 1.617088 | 3.067552 | 0.000608 | 0.023915 |
| [101956675](https://www.ncbi.nlm.nih.gov/entrez/query.fcgi?db=gene&cmd=Retrieve&dopt=full_report&list_uids=101956675) | Fam124b | 1.61475 | 3.062584 | 0.001074 | 0.037011 |
| [101973846](https://www.ncbi.nlm.nih.gov/entrez/query.fcgi?db=gene&cmd=Retrieve&dopt=full_report&list_uids=101973846) | Abcd2 | 1.603588 | 3.038981 | 0.000388 | 0.017014 |
| [101972436](https://www.ncbi.nlm.nih.gov/entrez/query.fcgi?db=gene&cmd=Retrieve&dopt=full_report&list_uids=101972436) | Calml6 | 1.601921 | 3.035472 | 0.000161 | 0.008969 |
| [101956119](https://www.ncbi.nlm.nih.gov/entrez/query.fcgi?db=gene&cmd=Retrieve&dopt=full_report&list_uids=101956119) | Atxn7l2 | 1.600178 | 3.031807 | 8.7E-05 | 0.0055 |
| [101971706](https://www.ncbi.nlm.nih.gov/entrez/query.fcgi?db=gene&cmd=Retrieve&dopt=full_report&list_uids=101971706) | LOC101971706 | 1.546508 | 2.921093 | 1.49E-05 | 0.001359 |
| [101976494](https://www.ncbi.nlm.nih.gov/entrez/query.fcgi?db=gene&cmd=Retrieve&dopt=full_report&list_uids=101976494) | Fam198a | 1.528258 | 2.884374 | 0.000587 | 0.023312 |
| [101966315](https://www.ncbi.nlm.nih.gov/entrez/query.fcgi?db=gene&cmd=Retrieve&dopt=full_report&list_uids=101966315) | Ppargc1b | 1.524757 | 2.877383 | 9.61E-05 | 0.00589 |
| [101978363](https://www.ncbi.nlm.nih.gov/entrez/query.fcgi?db=gene&cmd=Retrieve&dopt=full_report&list_uids=101978363) | Angptl2 | 1.524133 | 2.876138 | 5.97E-06 | 0.00068 |
| [101976178](https://www.ncbi.nlm.nih.gov/entrez/query.fcgi?db=gene&cmd=Retrieve&dopt=full_report&list_uids=101976178) | Fsd1l | 1.506285 | 2.840775 | 6.23E-06 | 0.000703 |
| [101976136](https://www.ncbi.nlm.nih.gov/entrez/query.fcgi?db=gene&cmd=Retrieve&dopt=full_report&list_uids=101976136) | Esr1 | 1.501291 | 2.830959 | 0.000284 | 0.013527 |
| [101960695](https://www.ncbi.nlm.nih.gov/entrez/query.fcgi?db=gene&cmd=Retrieve&dopt=full_report&list_uids=101960695) | LOC101960695 | 1.498895 | 2.826262 | 0.000238 | 0.012145 |
| [101963429](https://www.ncbi.nlm.nih.gov/entrez/query.fcgi?db=gene&cmd=Retrieve&dopt=full_report&list_uids=101963429) | Prkag3 | 1.476164 | 2.78208 | 0.000432 | 0.018433 |
| [101975796](https://www.ncbi.nlm.nih.gov/entrez/query.fcgi?db=gene&cmd=Retrieve&dopt=full_report&list_uids=101975796) | Slc43a3 | 1.473569 | 2.777081 | 4.17E-08 | 9.96E-06 |
| [101957767](https://www.ncbi.nlm.nih.gov/entrez/query.fcgi?db=gene&cmd=Retrieve&dopt=full_report&list_uids=101957767) | Gpc4 | 1.471684 | 2.773455 | 1.38E-05 | 0.001271 |
| [101968312](https://www.ncbi.nlm.nih.gov/entrez/query.fcgi?db=gene&cmd=Retrieve&dopt=full_report&list_uids=101968312) | Ttc32 | 1.455535 | 2.742583 | 0.000326 | 0.014943 |
| [101959942](https://www.ncbi.nlm.nih.gov/entrez/query.fcgi?db=gene&cmd=Retrieve&dopt=full_report&list_uids=101959942) | Pla2r1 | 1.437466 | 2.708447 | 0.000159 | 0.008902 |
| [101971461](https://www.ncbi.nlm.nih.gov/entrez/query.fcgi?db=gene&cmd=Retrieve&dopt=full_report&list_uids=101971461) | Prrx1 | 1.433864 | 2.701693 | 3.96E-05 | 0.002962 |
| [101976003](https://www.ncbi.nlm.nih.gov/entrez/query.fcgi?db=gene&cmd=Retrieve&dopt=full_report&list_uids=101976003) | Mlec | 1.432026 | 2.698253 | 2.56E-07 | 4.51E-05 |
| [101956179](https://www.ncbi.nlm.nih.gov/entrez/query.fcgi?db=gene&cmd=Retrieve&dopt=full_report&list_uids=101956179) | Cacng1 | 1.42186 | 2.679308 | 4.1E-06 | 0.000495 |
| [101975521](https://www.ncbi.nlm.nih.gov/entrez/query.fcgi?db=gene&cmd=Retrieve&dopt=full_report&list_uids=101975521) | Gadd45a | 1.415358 | 2.667259 | 0.000365 | 0.016304 |
| [101975445](https://www.ncbi.nlm.nih.gov/entrez/query.fcgi?db=gene&cmd=Retrieve&dopt=full_report&list_uids=101975445) | LOC101975445 | 1.414378 | 2.665448 | 5.68E-05 | 0.003816 |
| [101959700](https://www.ncbi.nlm.nih.gov/entrez/query.fcgi?db=gene&cmd=Retrieve&dopt=full_report&list_uids=101959700) | LOC101959700 | 1.413278 | 2.663416 | 0.000418 | 0.017993 |
| [101972179](https://www.ncbi.nlm.nih.gov/entrez/query.fcgi?db=gene&cmd=Retrieve&dopt=full_report&list_uids=101972179) | Pgpep1l | 1.399262 | 2.637667 | 0.001233 | 0.040707 |
| [101954641](https://www.ncbi.nlm.nih.gov/entrez/query.fcgi?db=gene&cmd=Retrieve&dopt=full_report&list_uids=101954641) | Pfkfb4 | 1.395677 | 2.63112 | 0.000276 | 0.013284 |
| [101975344](https://www.ncbi.nlm.nih.gov/entrez/query.fcgi?db=gene&cmd=Retrieve&dopt=full_report&list_uids=101975344) | Plce1 | 1.392043 | 2.624501 | 0.000726 | 0.027459 |
| [101975432](https://www.ncbi.nlm.nih.gov/entrez/query.fcgi?db=gene&cmd=Retrieve&dopt=full_report&list_uids=101975432) | Ampd1 | 1.389721 | 2.62028 | 0.000237 | 0.012145 |
| [101968177](https://www.ncbi.nlm.nih.gov/entrez/query.fcgi?db=gene&cmd=Retrieve&dopt=full_report&list_uids=101968177) | Amotl1 | 1.385652 | 2.6129 | 0.000601 | 0.023785 |
| [101971593](https://www.ncbi.nlm.nih.gov/entrez/query.fcgi?db=gene&cmd=Retrieve&dopt=full_report&list_uids=101971593) | Usp35 | 1.38194 | 2.606187 | 0.000543 | 0.022118 |
| [101962349](https://www.ncbi.nlm.nih.gov/entrez/query.fcgi?db=gene&cmd=Retrieve&dopt=full_report&list_uids=101962349) | Ahdc1 | 1.377134 | 2.597519 | 0.000258 | 0.012874 |
| [101957239](https://www.ncbi.nlm.nih.gov/entrez/query.fcgi?db=gene&cmd=Retrieve&dopt=full_report&list_uids=101957239) | Dok5 | 1.376521 | 2.596415 | 0.001598 | 0.049876 |
| [101973838](https://www.ncbi.nlm.nih.gov/entrez/query.fcgi?db=gene&cmd=Retrieve&dopt=full_report&list_uids=101973838) | Met | 1.368285 | 2.581634 | 7E-05 | 0.004551 |
| [101958402](https://www.ncbi.nlm.nih.gov/entrez/query.fcgi?db=gene&cmd=Retrieve&dopt=full_report&list_uids=101958402) | Tmem120b | 1.340729 | 2.532793 | 8.68E-06 | 0.00091 |
| [101958941](https://www.ncbi.nlm.nih.gov/entrez/query.fcgi?db=gene&cmd=Retrieve&dopt=full_report&list_uids=101958941) | Acsl4 | 1.338207 | 2.528369 | 1.6E-05 | 0.001409 |
| [101959472](https://www.ncbi.nlm.nih.gov/entrez/query.fcgi?db=gene&cmd=Retrieve&dopt=full_report&list_uids=101959472) | Rap2c | 1.311192 | 2.481465 | 4.24E-05 | 0.003089 |
| [101961155](https://www.ncbi.nlm.nih.gov/entrez/query.fcgi?db=gene&cmd=Retrieve&dopt=full_report&list_uids=101961155) | Zcwpw1 | 1.303915 | 2.46898 | 0.000997 | 0.035065 |
| [101960294](https://www.ncbi.nlm.nih.gov/entrez/query.fcgi?db=gene&cmd=Retrieve&dopt=full_report&list_uids=101960294) | Greb1l | 1.299391 | 2.461249 | 0.000399 | 0.017331 |
| [101965940](https://www.ncbi.nlm.nih.gov/entrez/query.fcgi?db=gene&cmd=Retrieve&dopt=full_report&list_uids=101965940) | Lpgat1 | 1.291086 | 2.447122 | 0.000985 | 0.034962 |
| [101955095](https://www.ncbi.nlm.nih.gov/entrez/query.fcgi?db=gene&cmd=Retrieve&dopt=full_report&list_uids=101955095) | Dyrk2 | 1.29016 | 2.445551 | 0.000229 | 0.011922 |
| [101970747](https://www.ncbi.nlm.nih.gov/entrez/query.fcgi?db=gene&cmd=Retrieve&dopt=full_report&list_uids=101970747) | Cxxc5 | 1.286223 | 2.438887 | 5.43E-06 | 0.000624 |
| [101974355](https://www.ncbi.nlm.nih.gov/entrez/query.fcgi?db=gene&cmd=Retrieve&dopt=full_report&list_uids=101974355) | Peg3 | 1.285547 | 2.437744 | 0.001141 | 0.03886 |
| [101958228](https://www.ncbi.nlm.nih.gov/entrez/query.fcgi?db=gene&cmd=Retrieve&dopt=full_report&list_uids=101958228) | Fbxl4 | 1.284798 | 2.43648 | 3.53E-05 | 0.002724 |
| [101960521](https://www.ncbi.nlm.nih.gov/entrez/query.fcgi?db=gene&cmd=Retrieve&dopt=full_report&list_uids=101960521) | Hes1 | 1.272512 | 2.415819 | 0.001102 | 0.037746 |
| [101954561](https://www.ncbi.nlm.nih.gov/entrez/query.fcgi?db=gene&cmd=Retrieve&dopt=full_report&list_uids=101954561) | Pdzrn3 | 1.268159 | 2.40854 | 2.06E-05 | 0.001722 |
| [101964725](https://www.ncbi.nlm.nih.gov/entrez/query.fcgi?db=gene&cmd=Retrieve&dopt=full_report&list_uids=101964725) | LOC101964725 | 1.267842 | 2.408012 | 0.000136 | 0.007816 |
| [101964202](https://www.ncbi.nlm.nih.gov/entrez/query.fcgi?db=gene&cmd=Retrieve&dopt=full_report&list_uids=101964202) | Gramd3 | 1.257437 | 2.390706 | 0.000504 | 0.020636 |
| [101974879](https://www.ncbi.nlm.nih.gov/entrez/query.fcgi?db=gene&cmd=Retrieve&dopt=full_report&list_uids=101974879) | Mamstr | 1.250805 | 2.379741 | 0.000996 | 0.035065 |
| [101971949](https://www.ncbi.nlm.nih.gov/entrez/query.fcgi?db=gene&cmd=Retrieve&dopt=full_report&list_uids=101971949) | Tmtc4 | 1.241131 | 2.363837 | 6.4E-05 | 0.004227 |
| [101966572](https://www.ncbi.nlm.nih.gov/entrez/query.fcgi?db=gene&cmd=Retrieve&dopt=full_report&list_uids=101966572) | Rnf122 | 1.235485 | 2.354605 | 8.43E-05 | 0.005361 |
| [101973349](https://www.ncbi.nlm.nih.gov/entrez/query.fcgi?db=gene&cmd=Retrieve&dopt=full_report&list_uids=101973349) | Sema3f | 1.227182 | 2.341093 | 0.000659 | 0.025572 |
| [101970608](https://www.ncbi.nlm.nih.gov/entrez/query.fcgi?db=gene&cmd=Retrieve&dopt=full_report&list_uids=101970608) | Commd8 | 1.218063 | 2.326342 | 0.001199 | 0.040263 |
| [101963671](https://www.ncbi.nlm.nih.gov/entrez/query.fcgi?db=gene&cmd=Retrieve&dopt=full_report&list_uids=101963671) | Uchl1 | 1.21162 | 2.315975 | 0.001177 | 0.039847 |
| [101958259](https://www.ncbi.nlm.nih.gov/entrez/query.fcgi?db=gene&cmd=Retrieve&dopt=full_report&list_uids=101958259) | Tarbp1 | 1.201308 | 2.29948 | 0.000557 | 0.022594 |
| [101977126](https://www.ncbi.nlm.nih.gov/entrez/query.fcgi?db=gene&cmd=Retrieve&dopt=full_report&list_uids=101977126) | Acsl6 | 1.195506 | 2.290251 | 6.53E-05 | 0.004292 |
| [101976297](https://www.ncbi.nlm.nih.gov/entrez/query.fcgi?db=gene&cmd=Retrieve&dopt=full_report&list_uids=101976297) | Myf6 | 1.192742 | 2.285868 | 0.000234 | 0.01209 |
| [101963168](https://www.ncbi.nlm.nih.gov/entrez/query.fcgi?db=gene&cmd=Retrieve&dopt=full_report&list_uids=101963168) | Impa2 | 1.188944 | 2.279859 | 9.59E-05 | 0.00589 |
| [101961575](https://www.ncbi.nlm.nih.gov/entrez/query.fcgi?db=gene&cmd=Retrieve&dopt=full_report&list_uids=101961575) | Eya1 | 1.185458 | 2.274357 | 0.000376 | 0.016566 |
| [101969032](https://www.ncbi.nlm.nih.gov/entrez/query.fcgi?db=gene&cmd=Retrieve&dopt=full_report&list_uids=101969032) | Sema6c | 1.177049 | 2.261138 | 0.000329 | 0.015031 |
| [101956072](https://www.ncbi.nlm.nih.gov/entrez/query.fcgi?db=gene&cmd=Retrieve&dopt=full_report&list_uids=101956072) | Fgf1 | 1.168102 | 2.247159 | 0.00044 | 0.018609 |
| [101972312](https://www.ncbi.nlm.nih.gov/entrez/query.fcgi?db=gene&cmd=Retrieve&dopt=full_report&list_uids=101972312) | Dagla | 1.1672 | 2.245754 | 0.000459 | 0.019173 |
| [101967129](https://www.ncbi.nlm.nih.gov/entrez/query.fcgi?db=gene&cmd=Retrieve&dopt=full_report&list_uids=101967129) | Hs3st5 | 1.164336 | 2.2413 | 0.000694 | 0.026586 |
| [101975276](https://www.ncbi.nlm.nih.gov/entrez/query.fcgi?db=gene&cmd=Retrieve&dopt=full_report&list_uids=101975276) | LOC101975276 | 1.156301 | 2.228852 | 0.000223 | 0.011789 |
| [101976422](https://www.ncbi.nlm.nih.gov/entrez/query.fcgi?db=gene&cmd=Retrieve&dopt=full_report&list_uids=101976422) | Hfe2 | 1.124058 | 2.179592 | 0.001229 | 0.040707 |
| [101978492](https://www.ncbi.nlm.nih.gov/entrez/query.fcgi?db=gene&cmd=Retrieve&dopt=full_report&list_uids=101978492) | B4galt5 | 1.116677 | 2.16847 | 0.000738 | 0.027824 |
| [101955620](https://www.ncbi.nlm.nih.gov/entrez/query.fcgi?db=gene&cmd=Retrieve&dopt=full_report&list_uids=101955620) | Trpm4 | 1.115195 | 2.166242 | 0.00042 | 0.018003 |
| [101960736](https://www.ncbi.nlm.nih.gov/entrez/query.fcgi?db=gene&cmd=Retrieve&dopt=full_report&list_uids=101960736) | Dhrs7b | 1.093223 | 2.133501 | 0.000667 | 0.025737 |
| [101958557](https://www.ncbi.nlm.nih.gov/entrez/query.fcgi?db=gene&cmd=Retrieve&dopt=full_report&list_uids=101958557) | Acyp1 | 1.087834 | 2.125546 | 0.001451 | 0.046499 |
| [101970501](https://www.ncbi.nlm.nih.gov/entrez/query.fcgi?db=gene&cmd=Retrieve&dopt=full_report&list_uids=101970501) | Ppm1l | 1.084965 | 2.121324 | 0.000878 | 0.031988 |
| [101955173](https://www.ncbi.nlm.nih.gov/entrez/query.fcgi?db=gene&cmd=Retrieve&dopt=full_report&list_uids=101955173) | Cnksr3 | 1.070714 | 2.100473 | 0.001411 | 0.045598 |
| [101978537](https://www.ncbi.nlm.nih.gov/entrez/query.fcgi?db=gene&cmd=Retrieve&dopt=full_report&list_uids=101978537) | P2rx5 | 1.069962 | 2.099379 | 0.001591 | 0.049808 |
| [101955019](https://www.ncbi.nlm.nih.gov/entrez/query.fcgi?db=gene&cmd=Retrieve&dopt=full_report&list_uids=101955019) | Slc25a42 | 1.060536 | 2.085707 | 0.000243 | 0.01228 |
| [101965785](https://www.ncbi.nlm.nih.gov/entrez/query.fcgi?db=gene&cmd=Retrieve&dopt=full_report&list_uids=101965785) | Rbm3 | 1.044105 | 2.062086 | 0.001521 | 0.047997 |
| [101959178](https://www.ncbi.nlm.nih.gov/entrez/query.fcgi?db=gene&cmd=Retrieve&dopt=full_report&list_uids=101959178) | Dyrk1b | 1.029227 | 2.040931 | 0.000789 | 0.029486 |
| [101961354](https://www.ncbi.nlm.nih.gov/entrez/query.fcgi?db=gene&cmd=Retrieve&dopt=full_report&list_uids=101961354) | Taf9b | 1.02884 | 2.040382 | 0.001171 | 0.039775 |
| [101967861](https://www.ncbi.nlm.nih.gov/entrez/query.fcgi?db=gene&cmd=Retrieve&dopt=full_report&list_uids=101967861) | Tmem64 | 1.027992 | 2.039184 | 0.000979 | 0.034838 |
| [101965762](https://www.ncbi.nlm.nih.gov/entrez/query.fcgi?db=gene&cmd=Retrieve&dopt=full_report&list_uids=101965762) | Stx10 | 1.000993 | 2.001377 | 0.001489 | 0.047214 |
| [101973922](https://www.ncbi.nlm.nih.gov/entrez/query.fcgi?db=gene&cmd=Retrieve&dopt=full_report&list_uids=101973922) | Smarcd2 | -0.92049 | -1.89276 | 0.001386 | 0.045031 |
| [101975752](https://www.ncbi.nlm.nih.gov/entrez/query.fcgi?db=gene&cmd=Retrieve&dopt=full_report&list_uids=101975752) | Mpst | -0.95921 | -1.94425 | 0.001131 | 0.038623 |
| [101977957](https://www.ncbi.nlm.nih.gov/entrez/query.fcgi?db=gene&cmd=Retrieve&dopt=full_report&list_uids=101977957) | Dynll1 | -0.96712 | -1.95493 | 0.000916 | 0.033016 |
| [101974141](https://www.ncbi.nlm.nih.gov/entrez/query.fcgi?db=gene&cmd=Retrieve&dopt=full_report&list_uids=101974141) | Tmem57 | -0.98365 | -1.97746 | 0.000456 | 0.01911 |
| [101975385](https://www.ncbi.nlm.nih.gov/entrez/query.fcgi?db=gene&cmd=Retrieve&dopt=full_report&list_uids=101975385) | Tob1 | -0.99207 | -1.98904 | 0.000344 | 0.015499 |
| [101974997](https://www.ncbi.nlm.nih.gov/entrez/query.fcgi?db=gene&cmd=Retrieve&dopt=full_report&list_uids=101974997) | Tsc22d1 | -0.99267 | -1.98986 | 0.00118 | 0.039847 |
| [101958121](https://www.ncbi.nlm.nih.gov/entrez/query.fcgi?db=gene&cmd=Retrieve&dopt=full_report&list_uids=101958121) | Mknk2 | -0.99883 | -1.99838 | 0.001482 | 0.047126 |
| [101968489](https://www.ncbi.nlm.nih.gov/entrez/query.fcgi?db=gene&cmd=Retrieve&dopt=full_report&list_uids=101968489) | Tagln2 | -1.00435 | -2.00603 | 0.000467 | 0.019448 |
| [101966665](https://www.ncbi.nlm.nih.gov/entrez/query.fcgi?db=gene&cmd=Retrieve&dopt=full_report&list_uids=101966665) | Rnf103 | -1.01234 | -2.01718 | 0.000836 | 0.030836 |
| [101971500](https://www.ncbi.nlm.nih.gov/entrez/query.fcgi?db=gene&cmd=Retrieve&dopt=full_report&list_uids=101971500) | Znf746 | -1.0125 | -2.0174 | 0.001443 | 0.046377 |
| [101961929](https://www.ncbi.nlm.nih.gov/entrez/query.fcgi?db=gene&cmd=Retrieve&dopt=full_report&list_uids=101961929) | Ell | -1.01929 | -2.02692 | 0.000567 | 0.022787 |
| [101958243](https://www.ncbi.nlm.nih.gov/entrez/query.fcgi?db=gene&cmd=Retrieve&dopt=full_report&list_uids=101958243) | Abhd4 | -1.02647 | -2.03704 | 0.000416 | 0.017936 |
| [101963604](https://www.ncbi.nlm.nih.gov/entrez/query.fcgi?db=gene&cmd=Retrieve&dopt=full_report&list_uids=101963604) | Art5 | -1.03357 | -2.04709 | 0.001358 | 0.044225 |
| [101954731](https://www.ncbi.nlm.nih.gov/entrez/query.fcgi?db=gene&cmd=Retrieve&dopt=full_report&list_uids=101954731) | Jun | -1.03571 | -2.05012 | 0.00082 | 0.030432 |
| [101968061](https://www.ncbi.nlm.nih.gov/entrez/query.fcgi?db=gene&cmd=Retrieve&dopt=full_report&list_uids=101968061) | Ndel1 | -1.04095 | -2.05758 | 0.001027 | 0.035907 |
| [101956139](https://www.ncbi.nlm.nih.gov/entrez/query.fcgi?db=gene&cmd=Retrieve&dopt=full_report&list_uids=101956139) | Klf6 | -1.0479 | -2.06752 | 0.000847 | 0.031163 |
| [101975186](https://www.ncbi.nlm.nih.gov/entrez/query.fcgi?db=gene&cmd=Retrieve&dopt=full_report&list_uids=101975186) | Auh | -1.05162 | -2.07285 | 0.000212 | 0.01131 |
| [101978066](https://www.ncbi.nlm.nih.gov/entrez/query.fcgi?db=gene&cmd=Retrieve&dopt=full_report&list_uids=101978066) | Rell1 | -1.05235 | -2.07391 | 0.001536 | 0.04833 |
| [101961037](https://www.ncbi.nlm.nih.gov/entrez/query.fcgi?db=gene&cmd=Retrieve&dopt=full_report&list_uids=101961037) | Phrf1 | -1.05557 | -2.07854 | 0.000168 | 0.009231 |
| [101970688](https://www.ncbi.nlm.nih.gov/entrez/query.fcgi?db=gene&cmd=Retrieve&dopt=full_report&list_uids=101970688) | Cdc42ep3 | -1.05628 | -2.07956 | 0.000315 | 0.014677 |
| [101977091](https://www.ncbi.nlm.nih.gov/entrez/query.fcgi?db=gene&cmd=Retrieve&dopt=full_report&list_uids=101977091) | Jak2 | -1.06464 | -2.09165 | 0.001494 | 0.047252 |
| [101967616](https://www.ncbi.nlm.nih.gov/entrez/query.fcgi?db=gene&cmd=Retrieve&dopt=full_report&list_uids=101967616) | Phc2 | -1.06671 | -2.09465 | 5.56E-05 | 0.003775 |
| [101977806](https://www.ncbi.nlm.nih.gov/entrez/query.fcgi?db=gene&cmd=Retrieve&dopt=full_report&list_uids=101977806) | Rnf115 | -1.08692 | -2.1242 | 0.001204 | 0.040328 |
| [101976351](https://www.ncbi.nlm.nih.gov/entrez/query.fcgi?db=gene&cmd=Retrieve&dopt=full_report&list_uids=101976351) | Tp53inp2 | -1.09293 | -2.13307 | 0.000661 | 0.025572 |
| [101960011](https://www.ncbi.nlm.nih.gov/entrez/query.fcgi?db=gene&cmd=Retrieve&dopt=full_report&list_uids=101960011) | LOC101960011 | -1.09814 | -2.14078 | 0.000917 | 0.033016 |
| [101957501](https://www.ncbi.nlm.nih.gov/entrez/query.fcgi?db=gene&cmd=Retrieve&dopt=full_report&list_uids=101957501) | Gpcpd1 | -1.11087 | -2.15975 | 9.39E-05 | 0.00582 |
| [101977068](https://www.ncbi.nlm.nih.gov/entrez/query.fcgi?db=gene&cmd=Retrieve&dopt=full_report&list_uids=101977068) | Net1 | -1.11213 | -2.16164 | 0.000218 | 0.011597 |
| [101968792](https://www.ncbi.nlm.nih.gov/entrez/query.fcgi?db=gene&cmd=Retrieve&dopt=full_report&list_uids=101968792) | Kiaa0226 | -1.11408 | -2.16457 | 0.000929 | 0.033257 |
| [101969139](https://www.ncbi.nlm.nih.gov/entrez/query.fcgi?db=gene&cmd=Retrieve&dopt=full_report&list_uids=101969139) | Cdc42ep4 | -1.1184 | -2.17106 | 0.001291 | 0.042297 |
| [101965956](https://www.ncbi.nlm.nih.gov/entrez/query.fcgi?db=gene&cmd=Retrieve&dopt=full_report&list_uids=101965956) | Pi4k2a | -1.12182 | -2.17621 | 0.000712 | 0.027199 |
| [101978641](https://www.ncbi.nlm.nih.gov/entrez/query.fcgi?db=gene&cmd=Retrieve&dopt=full_report&list_uids=101978641) | Tbc1d1 | -1.12315 | -2.17822 | 0.000367 | 0.016322 |
| [101977782](https://www.ncbi.nlm.nih.gov/entrez/query.fcgi?db=gene&cmd=Retrieve&dopt=full_report&list_uids=101977782) | Tinagl1 | -1.12512 | -2.18119 | 0.000119 | 0.006925 |
| [101976403](https://www.ncbi.nlm.nih.gov/entrez/query.fcgi?db=gene&cmd=Retrieve&dopt=full_report&list_uids=101976403) | Mpp6 | -1.12926 | -2.18746 | 0.000669 | 0.025737 |
| [101971271](https://www.ncbi.nlm.nih.gov/entrez/query.fcgi?db=gene&cmd=Retrieve&dopt=full_report&list_uids=101971271) | Mark4 | -1.13479 | -2.19587 | 0.00092 | 0.033016 |
| [101968946](https://www.ncbi.nlm.nih.gov/entrez/query.fcgi?db=gene&cmd=Retrieve&dopt=full_report&list_uids=101968946) | Nup50 | -1.14751 | -2.21532 | 0.001415 | 0.045599 |
| [101961552](https://www.ncbi.nlm.nih.gov/entrez/query.fcgi?db=gene&cmd=Retrieve&dopt=full_report&list_uids=101961552) | Selenbp1 | -1.1529 | -2.2236 | 4.4E-05 | 0.003148 |
| [101965112](https://www.ncbi.nlm.nih.gov/entrez/query.fcgi?db=gene&cmd=Retrieve&dopt=full_report&list_uids=101965112) | Klhl25 | -1.15491 | -2.2267 | 0.00011 | 0.006506 |
| [101976480](https://www.ncbi.nlm.nih.gov/entrez/query.fcgi?db=gene&cmd=Retrieve&dopt=full_report&list_uids=101976480) | Osgin1 | -1.16989 | -2.24995 | 0.000353 | 0.015879 |
| [101960347](https://www.ncbi.nlm.nih.gov/entrez/query.fcgi?db=gene&cmd=Retrieve&dopt=full_report&list_uids=101960347) | Btnl9 | -1.17007 | -2.25023 | 0.000722 | 0.027423 |
| [101966934](https://www.ncbi.nlm.nih.gov/entrez/query.fcgi?db=gene&cmd=Retrieve&dopt=full_report&list_uids=101966934) | Ppp2ca | -1.17827 | -2.26305 | 0.001053 | 0.036605 |
| [101977987](https://www.ncbi.nlm.nih.gov/entrez/query.fcgi?db=gene&cmd=Retrieve&dopt=full_report&list_uids=101977987) | Aldh6a1 | -1.18239 | -2.26953 | 5.44E-05 | 0.003718 |
| [101971000](https://www.ncbi.nlm.nih.gov/entrez/query.fcgi?db=gene&cmd=Retrieve&dopt=full_report&list_uids=101971000) | Ido1 | -1.1858 | -2.2749 | 0.00148 | 0.047126 |
| [101958403](https://www.ncbi.nlm.nih.gov/entrez/query.fcgi?db=gene&cmd=Retrieve&dopt=full_report&list_uids=101958403) | Ctgf | -1.18689 | -2.27661 | 0.00091 | 0.032974 |
| [101966521](https://www.ncbi.nlm.nih.gov/entrez/query.fcgi?db=gene&cmd=Retrieve&dopt=full_report&list_uids=101966521) | Zfand5 | -1.18743 | -2.27746 | 0.000166 | 0.009171 |
| [101975209](https://www.ncbi.nlm.nih.gov/entrez/query.fcgi?db=gene&cmd=Retrieve&dopt=full_report&list_uids=101975209) | Ets2 | -1.18893 | -2.27984 | 2.98E-05 | 0.002375 |
| [101955324](https://www.ncbi.nlm.nih.gov/entrez/query.fcgi?db=gene&cmd=Retrieve&dopt=full_report&list_uids=101955324) | Fkrp | -1.19897 | -2.29576 | 0.000484 | 0.01995 |
| [101965126](https://www.ncbi.nlm.nih.gov/entrez/query.fcgi?db=gene&cmd=Retrieve&dopt=full_report&list_uids=101965126) | Sun2 | -1.1996 | -2.29676 | 0.000264 | 0.013044 |
| [101967660](https://www.ncbi.nlm.nih.gov/entrez/query.fcgi?db=gene&cmd=Retrieve&dopt=full_report&list_uids=101967660) | Nupr1 | -1.20385 | -2.30353 | 0.000243 | 0.01228 |
| [101960834](https://www.ncbi.nlm.nih.gov/entrez/query.fcgi?db=gene&cmd=Retrieve&dopt=full_report&list_uids=101960834) | Homer2 | -1.20548 | -2.30614 | 9.23E-05 | 0.005748 |
| [101971550](https://www.ncbi.nlm.nih.gov/entrez/query.fcgi?db=gene&cmd=Retrieve&dopt=full_report&list_uids=101971550) | Plcd1 | -1.20628 | -2.30742 | 0.000563 | 0.022738 |
| [101976415](https://www.ncbi.nlm.nih.gov/entrez/query.fcgi?db=gene&cmd=Retrieve&dopt=full_report&list_uids=101976415) | Srxn1 | -1.21024 | -2.31377 | 0.001267 | 0.041619 |
| [101977802](https://www.ncbi.nlm.nih.gov/entrez/query.fcgi?db=gene&cmd=Retrieve&dopt=full_report&list_uids=101977802) | Bag3 | -1.21379 | -2.31947 | 0.000237 | 0.012145 |
| [101977600](https://www.ncbi.nlm.nih.gov/entrez/query.fcgi?db=gene&cmd=Retrieve&dopt=full_report&list_uids=101977600) | Ivd | -1.2146 | -2.32076 | 0.001057 | 0.036619 |
| [101955208](https://www.ncbi.nlm.nih.gov/entrez/query.fcgi?db=gene&cmd=Retrieve&dopt=full_report&list_uids=101955208) | Pm20d1 | -1.21515 | -2.32165 | 0.001467 | 0.046889 |
| [101961237](https://www.ncbi.nlm.nih.gov/entrez/query.fcgi?db=gene&cmd=Retrieve&dopt=full_report&list_uids=101961237) | Diaph1 | -1.21944 | -2.32856 | 0.000749 | 0.028153 |
| [101968165](https://www.ncbi.nlm.nih.gov/entrez/query.fcgi?db=gene&cmd=Retrieve&dopt=full_report&list_uids=101968165) | Bicc1 | -1.22054 | -2.33035 | 0.000654 | 0.025565 |
| [101977147](https://www.ncbi.nlm.nih.gov/entrez/query.fcgi?db=gene&cmd=Retrieve&dopt=full_report&list_uids=101977147) | Pde4d | -1.22631 | -2.33967 | 0.000339 | 0.015359 |
| [101957365](https://www.ncbi.nlm.nih.gov/entrez/query.fcgi?db=gene&cmd=Retrieve&dopt=full_report&list_uids=101957365) | Apold1 | -1.23454 | -2.35306 | 0.000105 | 0.006336 |
| [101960771](https://www.ncbi.nlm.nih.gov/entrez/query.fcgi?db=gene&cmd=Retrieve&dopt=full_report&list_uids=101960771) | Hsd3b7 | -1.2348 | -2.35349 | 0.00123 | 0.040707 |
| [106145291](https://www.ncbi.nlm.nih.gov/entrez/query.fcgi?db=gene&cmd=Retrieve&dopt=full_report&list_uids=106145291) | LOC106145291 | -1.24064 | -2.36304 | 4.62E-05 | 0.00321 |
| [101965076](https://www.ncbi.nlm.nih.gov/entrez/query.fcgi?db=gene&cmd=Retrieve&dopt=full_report&list_uids=101965076) | Arrdc3 | -1.24922 | -2.37714 | 2.33E-05 | 0.001921 |
| [101976065](https://www.ncbi.nlm.nih.gov/entrez/query.fcgi?db=gene&cmd=Retrieve&dopt=full_report&list_uids=101976065) | Nr1h3 | -1.25392 | -2.38488 | 0.000377 | 0.016566 |
| [101971367](https://www.ncbi.nlm.nih.gov/entrez/query.fcgi?db=gene&cmd=Retrieve&dopt=full_report&list_uids=101971367) | Limd1 | -1.25938 | -2.39392 | 0.000269 | 0.013203 |
| [101975915](https://www.ncbi.nlm.nih.gov/entrez/query.fcgi?db=gene&cmd=Retrieve&dopt=full_report&list_uids=101975915) | Bcat2 | -1.26501 | -2.40328 | 7.11E-05 | 0.004593 |
| [101956216](https://www.ncbi.nlm.nih.gov/entrez/query.fcgi?db=gene&cmd=Retrieve&dopt=full_report&list_uids=101956216) | Nrg4 | -1.26675 | -2.40619 | 0.00013 | 0.007544 |
| [101974568](https://www.ncbi.nlm.nih.gov/entrez/query.fcgi?db=gene&cmd=Retrieve&dopt=full_report&list_uids=101974568) | Csrp2 | -1.27589 | -2.42149 | 0.000781 | 0.02927 |
| [101962368](https://www.ncbi.nlm.nih.gov/entrez/query.fcgi?db=gene&cmd=Retrieve&dopt=full_report&list_uids=101962368) | Slc25a25 | -1.28138 | -2.43071 | 0.000226 | 0.011814 |
| [101974952](https://www.ncbi.nlm.nih.gov/entrez/query.fcgi?db=gene&cmd=Retrieve&dopt=full_report&list_uids=101974952) | Mcl1 | -1.29048 | -2.4461 | 0.00019 | 0.010262 |
| [101967070](https://www.ncbi.nlm.nih.gov/entrez/query.fcgi?db=gene&cmd=Retrieve&dopt=full_report&list_uids=101967070) | Bdh1 | -1.29548 | -2.45458 | 0.001069 | 0.036937 |
| [101956221](https://www.ncbi.nlm.nih.gov/entrez/query.fcgi?db=gene&cmd=Retrieve&dopt=full_report&list_uids=101956221) | Hp1bp3 | -1.29785 | -2.45862 | 4.51E-05 | 0.003181 |
| [101965681](https://www.ncbi.nlm.nih.gov/entrez/query.fcgi?db=gene&cmd=Retrieve&dopt=full_report&list_uids=101965681) | Pla2g15 | -1.29829 | -2.45936 | 3.48E-05 | 0.0027 |
| [101957970](https://www.ncbi.nlm.nih.gov/entrez/query.fcgi?db=gene&cmd=Retrieve&dopt=full_report&list_uids=101957970) | Idi1 | -1.31053 | -2.48033 | 9.21E-05 | 0.005748 |
| [101961235](https://www.ncbi.nlm.nih.gov/entrez/query.fcgi?db=gene&cmd=Retrieve&dopt=full_report&list_uids=101961235) | LOC101961235 | -1.31126 | -2.48158 | 3.27E-05 | 0.002556 |
| [101972342](https://www.ncbi.nlm.nih.gov/entrez/query.fcgi?db=gene&cmd=Retrieve&dopt=full_report&list_uids=101972342) | Ier5 | -1.32418 | -2.5039 | 4.36E-05 | 0.003142 |
| [101970591](https://www.ncbi.nlm.nih.gov/entrez/query.fcgi?db=gene&cmd=Retrieve&dopt=full_report&list_uids=101970591) | Tmem150a | -1.32759 | -2.50983 | 5.63E-05 | 0.003803 |
| [101978217](https://www.ncbi.nlm.nih.gov/entrez/query.fcgi?db=gene&cmd=Retrieve&dopt=full_report&list_uids=101978217) | Dnajb4 | -1.339 | -2.52976 | 8.09E-06 | 0.000856 |
| [101966350](https://www.ncbi.nlm.nih.gov/entrez/query.fcgi?db=gene&cmd=Retrieve&dopt=full_report&list_uids=101966350) | Sik2 | -1.34144 | -2.53405 | 0.00036 | 0.016132 |
| [101954413](https://www.ncbi.nlm.nih.gov/entrez/query.fcgi?db=gene&cmd=Retrieve&dopt=full_report&list_uids=101954413) | Snx10 | -1.34362 | -2.53787 | 0.000586 | 0.023312 |
| [101976149](https://www.ncbi.nlm.nih.gov/entrez/query.fcgi?db=gene&cmd=Retrieve&dopt=full_report&list_uids=101976149) | Hspa2 | -1.34613 | -2.54229 | 0.000435 | 0.018514 |
| [101963449](https://www.ncbi.nlm.nih.gov/entrez/query.fcgi?db=gene&cmd=Retrieve&dopt=full_report&list_uids=101963449) | Ddt | -1.34827 | -2.54607 | 0.000158 | 0.008902 |
| [101966249](https://www.ncbi.nlm.nih.gov/entrez/query.fcgi?db=gene&cmd=Retrieve&dopt=full_report&list_uids=101966249) | Lrrc2 | -1.34839 | -2.54627 | 3.14E-05 | 0.002469 |
| [101956759](https://www.ncbi.nlm.nih.gov/entrez/query.fcgi?db=gene&cmd=Retrieve&dopt=full_report&list_uids=101956759) | Slc43a2 | -1.35143 | -2.55164 | 0.000319 | 0.014744 |
| [101975333](https://www.ncbi.nlm.nih.gov/entrez/query.fcgi?db=gene&cmd=Retrieve&dopt=full_report&list_uids=101975333) | Slc4a3 | -1.36425 | -2.57443 | 0.000657 | 0.025572 |
| [101959712](https://www.ncbi.nlm.nih.gov/entrez/query.fcgi?db=gene&cmd=Retrieve&dopt=full_report&list_uids=101959712) | Shb | -1.36609 | -2.5777 | 0.000825 | 0.030532 |
| [101978075](https://www.ncbi.nlm.nih.gov/entrez/query.fcgi?db=gene&cmd=Retrieve&dopt=full_report&list_uids=101978075) | Fam160b1 | -1.36713 | -2.57956 | 0.000162 | 0.008995 |
| [101958010](https://www.ncbi.nlm.nih.gov/entrez/query.fcgi?db=gene&cmd=Retrieve&dopt=full_report&list_uids=101958010) | Map3k14 | -1.37958 | -2.60193 | 0.000315 | 0.014677 |
| [101963881](https://www.ncbi.nlm.nih.gov/entrez/query.fcgi?db=gene&cmd=Retrieve&dopt=full_report&list_uids=101963881) | Dusp8 | -1.38454 | -2.61089 | 5.08E-05 | 0.003513 |
| [101954990](https://www.ncbi.nlm.nih.gov/entrez/query.fcgi?db=gene&cmd=Retrieve&dopt=full_report&list_uids=101954990) | LOC101954990 | -1.38955 | -2.61996 | 0.000571 | 0.022844 |
| [101966808](https://www.ncbi.nlm.nih.gov/entrez/query.fcgi?db=gene&cmd=Retrieve&dopt=full_report&list_uids=101966808) | Aldh1a1 | -1.39158 | -2.62366 | 1.33E-05 | 0.001237 |
| [101972491](https://www.ncbi.nlm.nih.gov/entrez/query.fcgi?db=gene&cmd=Retrieve&dopt=full_report&list_uids=101972491) | Tmem140 | -1.39268 | -2.62565 | 0.000336 | 0.015293 |
| [101978461](https://www.ncbi.nlm.nih.gov/entrez/query.fcgi?db=gene&cmd=Retrieve&dopt=full_report&list_uids=101978461) | Ankrd9 | -1.39652 | -2.63266 | 0.000154 | 0.008786 |
| [101976524](https://www.ncbi.nlm.nih.gov/entrez/query.fcgi?db=gene&cmd=Retrieve&dopt=full_report&list_uids=101976524) | Gabarapl1 | -1.4013 | -2.6414 | 0.000568 | 0.022787 |
| [101964036](https://www.ncbi.nlm.nih.gov/entrez/query.fcgi?db=gene&cmd=Retrieve&dopt=full_report&list_uids=101964036) | Arid5b | -1.40167 | -2.64207 | 0.000116 | 0.006816 |
| [101967772](https://www.ncbi.nlm.nih.gov/entrez/query.fcgi?db=gene&cmd=Retrieve&dopt=full_report&list_uids=101967772) | Pde8b | -1.40272 | -2.64399 | 0.001192 | 0.040145 |
| [101970189](https://www.ncbi.nlm.nih.gov/entrez/query.fcgi?db=gene&cmd=Retrieve&dopt=full_report&list_uids=101970189) | Acot7 | -1.40298 | -2.64448 | 2.14E-05 | 0.001779 |
| [101967414](https://www.ncbi.nlm.nih.gov/entrez/query.fcgi?db=gene&cmd=Retrieve&dopt=full_report&list_uids=101967414) | Hpgds | -1.41612 | -2.66866 | 7.16E-05 | 0.004599 |
| [101972020](https://www.ncbi.nlm.nih.gov/entrez/query.fcgi?db=gene&cmd=Retrieve&dopt=full_report&list_uids=101972020) | Crem | -1.42873 | -2.69209 | 6.91E-06 | 0.000758 |
| [101978638](https://www.ncbi.nlm.nih.gov/entrez/query.fcgi?db=gene&cmd=Retrieve&dopt=full_report&list_uids=101978638) | Dusp28 | -1.43324 | -2.70052 | 0.000112 | 0.006616 |
| [101956002](https://www.ncbi.nlm.nih.gov/entrez/query.fcgi?db=gene&cmd=Retrieve&dopt=full_report&list_uids=101956002) | Fbxo30 | -1.44305 | -2.71894 | 1.6E-05 | 0.001409 |
| [101955242](https://www.ncbi.nlm.nih.gov/entrez/query.fcgi?db=gene&cmd=Retrieve&dopt=full_report&list_uids=101955242) | LOC101955242 | -1.44493 | -2.7225 | 1.59E-05 | 0.001409 |
| [101961816](https://www.ncbi.nlm.nih.gov/entrez/query.fcgi?db=gene&cmd=Retrieve&dopt=full_report&list_uids=101961816) | Ube2j1 | -1.45588 | -2.74323 | 1.14E-05 | 0.001101 |
| [101956290](https://www.ncbi.nlm.nih.gov/entrez/query.fcgi?db=gene&cmd=Retrieve&dopt=full_report&list_uids=101956290) | Ppp1r3c | -1.45628 | -2.74401 | 0.000226 | 0.011814 |
| [101957276](https://www.ncbi.nlm.nih.gov/entrez/query.fcgi?db=gene&cmd=Retrieve&dopt=full_report&list_uids=101957276) | Zfp36 | -1.47405 | -2.778 | 3.7E-07 | 6.34E-05 |
| [101978543](https://www.ncbi.nlm.nih.gov/entrez/query.fcgi?db=gene&cmd=Retrieve&dopt=full_report&list_uids=101978543) | Cetp | -1.48502 | -2.79922 | 2.5E-06 | 0.000319 |
| [101971235](https://www.ncbi.nlm.nih.gov/entrez/query.fcgi?db=gene&cmd=Retrieve&dopt=full_report&list_uids=101971235) | Ccdc92 | -1.49504 | -2.81873 | 3.72E-05 | 0.002814 |
| [101973720](https://www.ncbi.nlm.nih.gov/entrez/query.fcgi?db=gene&cmd=Retrieve&dopt=full_report&list_uids=101973720) | Rapgef3 | -1.4973 | -2.82313 | 8.92E-06 | 0.000917 |
| [101977803](https://www.ncbi.nlm.nih.gov/entrez/query.fcgi?db=gene&cmd=Retrieve&dopt=full_report&list_uids=101977803) | Ablim1 | -1.49778 | -2.82408 | 0.000204 | 0.010937 |
| [101970431](https://www.ncbi.nlm.nih.gov/entrez/query.fcgi?db=gene&cmd=Retrieve&dopt=full_report&list_uids=101970431) | LOC101970431 | -1.50834 | -2.84483 | 6.36E-06 | 0.00071 |
| [101966799](https://www.ncbi.nlm.nih.gov/entrez/query.fcgi?db=gene&cmd=Retrieve&dopt=full_report&list_uids=101966799) | Nfkbia | -1.51021 | -2.84852 | 6.82E-08 | 1.48E-05 |
| [101956930](https://www.ncbi.nlm.nih.gov/entrez/query.fcgi?db=gene&cmd=Retrieve&dopt=full_report&list_uids=101956930) | Slc22a3 | -1.51717 | -2.86228 | 2.48E-05 | 0.002008 |
| [101976503](https://www.ncbi.nlm.nih.gov/entrez/query.fcgi?db=gene&cmd=Retrieve&dopt=full_report&list_uids=101976503) | Prom1 | -1.51977 | -2.86746 | 9.05E-06 | 0.000917 |
| [101963338](https://www.ncbi.nlm.nih.gov/entrez/query.fcgi?db=gene&cmd=Retrieve&dopt=full_report&list_uids=101963338) | Npc1 | -1.52484 | -2.87755 | 0.000288 | 0.013652 |
| [101961916](https://www.ncbi.nlm.nih.gov/entrez/query.fcgi?db=gene&cmd=Retrieve&dopt=full_report&list_uids=101961916) | Abca5 | -1.52982 | -2.8875 | 6.71E-06 | 0.000743 |
| [101962239](https://www.ncbi.nlm.nih.gov/entrez/query.fcgi?db=gene&cmd=Retrieve&dopt=full_report&list_uids=101962239) | Ptpru | -1.53598 | -2.89985 | 0.000241 | 0.012262 |
| [101975322](https://www.ncbi.nlm.nih.gov/entrez/query.fcgi?db=gene&cmd=Retrieve&dopt=full_report&list_uids=101975322) | Tfdp2 | -1.54277 | -2.91353 | 7.38E-06 | 0.000795 |
| [101969120](https://www.ncbi.nlm.nih.gov/entrez/query.fcgi?db=gene&cmd=Retrieve&dopt=full_report&list_uids=101969120) | Ldhb | -1.55078 | -2.92975 | 0.00104 | 0.036243 |
| [101969672](https://www.ncbi.nlm.nih.gov/entrez/query.fcgi?db=gene&cmd=Retrieve&dopt=full_report&list_uids=101969672) | Mid1ip1 | -1.5583 | -2.94507 | 1.04E-05 | 0.001018 |
| [101970566](https://www.ncbi.nlm.nih.gov/entrez/query.fcgi?db=gene&cmd=Retrieve&dopt=full_report&list_uids=101970566) | St6galnac2 | -1.5603 | -2.94916 | 0.000817 | 0.030432 |
| [101964315](https://www.ncbi.nlm.nih.gov/entrez/query.fcgi?db=gene&cmd=Retrieve&dopt=full_report&list_uids=101964315) | LOC101964315 | -1.56378 | -2.95628 | 7.24E-06 | 0.000786 |
| [101958679](https://www.ncbi.nlm.nih.gov/entrez/query.fcgi?db=gene&cmd=Retrieve&dopt=full_report&list_uids=101958679) | Gna13 | -1.56583 | -2.96047 | 9.2E-07 | 0.000145 |
| [101970847](https://www.ncbi.nlm.nih.gov/entrez/query.fcgi?db=gene&cmd=Retrieve&dopt=full_report&list_uids=101970847) | Slc2a4 | -1.56646 | -2.96176 | 1.31E-06 | 0.000191 |
| [101968280](https://www.ncbi.nlm.nih.gov/entrez/query.fcgi?db=gene&cmd=Retrieve&dopt=full_report&list_uids=101968280) | Gpd1l | -1.5792 | -2.98804 | 1.83E-05 | 0.001559 |
| [101957921](https://www.ncbi.nlm.nih.gov/entrez/query.fcgi?db=gene&cmd=Retrieve&dopt=full_report&list_uids=101957921) | Foxo3 | -1.59565 | -3.0223 | 1.03E-05 | 0.001016 |
| [101970527](https://www.ncbi.nlm.nih.gov/entrez/query.fcgi?db=gene&cmd=Retrieve&dopt=full_report&list_uids=101970527) | Bcl9 | -1.61112 | -3.0549 | 0.000391 | 0.017073 |
| [101959540](https://www.ncbi.nlm.nih.gov/entrez/query.fcgi?db=gene&cmd=Retrieve&dopt=full_report&list_uids=101959540) | Slc3a2 | -1.61433 | -3.06169 | 5.89E-05 | 0.003919 |
| [101964136](https://www.ncbi.nlm.nih.gov/entrez/query.fcgi?db=gene&cmd=Retrieve&dopt=full_report&list_uids=101964136) | Sec24a | -1.623 | -3.08014 | 9.99E-05 | 0.006038 |
| [101976393](https://www.ncbi.nlm.nih.gov/entrez/query.fcgi?db=gene&cmd=Retrieve&dopt=full_report&list_uids=101976393) | Mob3c | -1.63164 | -3.09866 | 0.001555 | 0.048784 |
| [101956105](https://www.ncbi.nlm.nih.gov/entrez/query.fcgi?db=gene&cmd=Retrieve&dopt=full_report&list_uids=101956105) | Slc29a2 | -1.63701 | -3.11021 | 0.000481 | 0.01995 |
| [101957628](https://www.ncbi.nlm.nih.gov/entrez/query.fcgi?db=gene&cmd=Retrieve&dopt=full_report&list_uids=101957628) | Slc26a7 | -1.64135 | -3.11957 | 0.001229 | 0.040707 |
| [101971609](https://www.ncbi.nlm.nih.gov/entrez/query.fcgi?db=gene&cmd=Retrieve&dopt=full_report&list_uids=101971609) | Mpped2 | -1.64367 | -3.12459 | 0.000866 | 0.031663 |
| [101957375](https://www.ncbi.nlm.nih.gov/entrez/query.fcgi?db=gene&cmd=Retrieve&dopt=full_report&list_uids=101957375) | Gdf11 | -1.65446 | -3.14806 | 0.000314 | 0.014677 |
| [101966661](https://www.ncbi.nlm.nih.gov/entrez/query.fcgi?db=gene&cmd=Retrieve&dopt=full_report&list_uids=101966661) | Cblb | -1.65618 | -3.15182 | 7.28E-05 | 0.004652 |
| [101956544](https://www.ncbi.nlm.nih.gov/entrez/query.fcgi?db=gene&cmd=Retrieve&dopt=full_report&list_uids=101956544) | Hmox1 | -1.66532 | -3.17184 | 1.21E-05 | 0.001152 |
| [101970676](https://www.ncbi.nlm.nih.gov/entrez/query.fcgi?db=gene&cmd=Retrieve&dopt=full_report&list_uids=101970676) | LOC101970676 | -1.6702 | -3.1826 | 4.4E-06 | 0.000516 |
| [101967243](https://www.ncbi.nlm.nih.gov/entrez/query.fcgi?db=gene&cmd=Retrieve&dopt=full_report&list_uids=101967243) | Galnt15 | -1.69671 | -3.2416 | 8.04E-06 | 0.000856 |
| [101964605](https://www.ncbi.nlm.nih.gov/entrez/query.fcgi?db=gene&cmd=Retrieve&dopt=full_report&list_uids=101964605) | Traf3ip2 | -1.70799 | -3.26705 | 0.000244 | 0.01228 |
| [101962792](https://www.ncbi.nlm.nih.gov/entrez/query.fcgi?db=gene&cmd=Retrieve&dopt=full_report&list_uids=101962792) | Fbxo32 | -1.71291 | -3.27821 | 0.000279 | 0.013366 |
| [101969304](https://www.ncbi.nlm.nih.gov/entrez/query.fcgi?db=gene&cmd=Retrieve&dopt=full_report&list_uids=101969304) | Lpl | -1.71872 | -3.29143 | 1.25E-05 | 0.001176 |
| [101968849](https://www.ncbi.nlm.nih.gov/entrez/query.fcgi?db=gene&cmd=Retrieve&dopt=full_report&list_uids=101968849) | Rgcc | -1.72911 | -3.31523 | 4.62E-05 | 0.00321 |
| [101973939](https://www.ncbi.nlm.nih.gov/entrez/query.fcgi?db=gene&cmd=Retrieve&dopt=full_report&list_uids=101973939) | Aass | -1.72931 | -3.31568 | 0.000484 | 0.01995 |
| [101954349](https://www.ncbi.nlm.nih.gov/entrez/query.fcgi?db=gene&cmd=Retrieve&dopt=full_report&list_uids=101954349) | Kcnma1 | -1.73269 | -3.32348 | 0.000108 | 0.006411 |
| [101956737](https://www.ncbi.nlm.nih.gov/entrez/query.fcgi?db=gene&cmd=Retrieve&dopt=full_report&list_uids=101956737) | Slc39a14 | -1.73456 | -3.32778 | 0.001262 | 0.041558 |
| [101959639](https://www.ncbi.nlm.nih.gov/entrez/query.fcgi?db=gene&cmd=Retrieve&dopt=full_report&list_uids=101959639) | Il6r | -1.736 | -3.3311 | 3.87E-05 | 0.002912 |
| [101957463](https://www.ncbi.nlm.nih.gov/entrez/query.fcgi?db=gene&cmd=Retrieve&dopt=full_report&list_uids=101957463) | Pnpla8 | -1.74166 | -3.3442 | 0.000177 | 0.009625 |
| [101961877](https://www.ncbi.nlm.nih.gov/entrez/query.fcgi?db=gene&cmd=Retrieve&dopt=full_report&list_uids=101961877) | Cdkn1a | -1.74782 | -3.3585 | 0.000173 | 0.009434 |
| [101960006](https://www.ncbi.nlm.nih.gov/entrez/query.fcgi?db=gene&cmd=Retrieve&dopt=full_report&list_uids=101960006) | Enah | -1.75155 | -3.36719 | 1.51E-06 | 0.000215 |
| [101959140](https://www.ncbi.nlm.nih.gov/entrez/query.fcgi?db=gene&cmd=Retrieve&dopt=full_report&list_uids=101959140) | Dgat2 | -1.7544 | -3.37385 | 1E-05 | 0.001008 |
| [101974771](https://www.ncbi.nlm.nih.gov/entrez/query.fcgi?db=gene&cmd=Retrieve&dopt=full_report&list_uids=101974771) | Phlda3 | -1.75497 | -3.3752 | 2.77E-06 | 0.000345 |
| [101955906](https://www.ncbi.nlm.nih.gov/entrez/query.fcgi?db=gene&cmd=Retrieve&dopt=full_report&list_uids=101955906) | Fzd1 | -1.76507 | -3.3989 | 6.09E-08 | 1.38E-05 |
| [101957267](https://www.ncbi.nlm.nih.gov/entrez/query.fcgi?db=gene&cmd=Retrieve&dopt=full_report&list_uids=101957267) | Zbtb16 | -1.77075 | -3.41232 | 4.52E-07 | 7.51E-05 |
| [101968459](https://www.ncbi.nlm.nih.gov/entrez/query.fcgi?db=gene&cmd=Retrieve&dopt=full_report&list_uids=101968459) | Wbp1l | -1.77898 | -3.43183 | 8.17E-08 | 1.68E-05 |
| [101959549](https://www.ncbi.nlm.nih.gov/entrez/query.fcgi?db=gene&cmd=Retrieve&dopt=full_report&list_uids=101959549) | LOC101959549 | -1.78016 | -3.43464 | 0.000277 | 0.013284 |
| [101958963](https://www.ncbi.nlm.nih.gov/entrez/query.fcgi?db=gene&cmd=Retrieve&dopt=full_report&list_uids=101958963) | LOC101958963 | -1.79099 | -3.46052 | 7.91E-07 | 0.000128 |
| [101965763](https://www.ncbi.nlm.nih.gov/entrez/query.fcgi?db=gene&cmd=Retrieve&dopt=full_report&list_uids=101965763) | Ulk1 | -1.79366 | -3.46693 | 3.74E-06 | 0.000461 |
| [101962360](https://www.ncbi.nlm.nih.gov/entrez/query.fcgi?db=gene&cmd=Retrieve&dopt=full_report&list_uids=101962360) | Pik3r1 | -1.81308 | -3.51393 | 1.24E-06 | 0.000186 |
| [101958858](https://www.ncbi.nlm.nih.gov/entrez/query.fcgi?db=gene&cmd=Retrieve&dopt=full_report&list_uids=101958858) | Mettl7a | -1.81566 | -3.52021 | 6.42E-08 | 1.42E-05 |
| [101965571](https://www.ncbi.nlm.nih.gov/entrez/query.fcgi?db=gene&cmd=Retrieve&dopt=full_report&list_uids=101965571) | Ret | -1.81567 | -3.52023 | 9.74E-05 | 0.00594 |
| [101965950](https://www.ncbi.nlm.nih.gov/entrez/query.fcgi?db=gene&cmd=Retrieve&dopt=full_report&list_uids=101965950) | LOC101965950 | -1.81688 | -3.52318 | 0.000491 | 0.020175 |
| [101956053](https://www.ncbi.nlm.nih.gov/entrez/query.fcgi?db=gene&cmd=Retrieve&dopt=full_report&list_uids=101956053) | Myoz2 | -1.82463 | -3.54216 | 0.000858 | 0.031485 |
| [101974277](https://www.ncbi.nlm.nih.gov/entrez/query.fcgi?db=gene&cmd=Retrieve&dopt=full_report&list_uids=101974277) | Hecw2 | -1.82567 | -3.54472 | 3.14E-05 | 0.002469 |
| [101977521](https://www.ncbi.nlm.nih.gov/entrez/query.fcgi?db=gene&cmd=Retrieve&dopt=full_report&list_uids=101977521) | Sema4b | -1.82949 | -3.55412 | 4.15E-08 | 9.96E-06 |
| [101962491](https://www.ncbi.nlm.nih.gov/entrez/query.fcgi?db=gene&cmd=Retrieve&dopt=full_report&list_uids=101962491) | Actg2 | -1.83914 | -3.57796 | 0.000313 | 0.014677 |
| [101960238](https://www.ncbi.nlm.nih.gov/entrez/query.fcgi?db=gene&cmd=Retrieve&dopt=full_report&list_uids=101960238) | Exosc6 | -1.85868 | -3.62675 | 0.000141 | 0.008089 |
| [101954518](https://www.ncbi.nlm.nih.gov/entrez/query.fcgi?db=gene&cmd=Retrieve&dopt=full_report&list_uids=101954518) | Tob2 | -1.87081 | -3.65738 | 1.95E-10 | 8.62E-08 |
| [101967355](https://www.ncbi.nlm.nih.gov/entrez/query.fcgi?db=gene&cmd=Retrieve&dopt=full_report&list_uids=101967355) | Dhtkd1 | -1.87672 | -3.67239 | 4.21E-05 | 0.003089 |
| [101974624](https://www.ncbi.nlm.nih.gov/entrez/query.fcgi?db=gene&cmd=Retrieve&dopt=full_report&list_uids=101974624) | Gadd45g | -1.88503 | -3.6936 | 1.67E-07 | 3.13E-05 |
| [101962514](https://www.ncbi.nlm.nih.gov/entrez/query.fcgi?db=gene&cmd=Retrieve&dopt=full_report&list_uids=101962514) | Klhl38 | -1.90558 | -3.74659 | 4.06E-08 | 9.96E-06 |
| [101965304](https://www.ncbi.nlm.nih.gov/entrez/query.fcgi?db=gene&cmd=Retrieve&dopt=full_report&list_uids=101965304) | Dgkh | -1.90685 | -3.7499 | 4.52E-05 | 0.003181 |
| [101958764](https://www.ncbi.nlm.nih.gov/entrez/query.fcgi?db=gene&cmd=Retrieve&dopt=full_report&list_uids=101958764) | Slc38a2 | -1.92232 | -3.79033 | 1.16E-06 | 0.000178 |
| [101974959](https://www.ncbi.nlm.nih.gov/entrez/query.fcgi?db=gene&cmd=Retrieve&dopt=full_report&list_uids=101974959) | Rhod | -1.93718 | -3.82956 | 0.000272 | 0.013284 |
| [101974015](https://www.ncbi.nlm.nih.gov/entrez/query.fcgi?db=gene&cmd=Retrieve&dopt=full_report&list_uids=101974015) | St6galnac3 | -1.94012 | -3.83738 | 0.000409 | 0.017698 |
| [101977394](https://www.ncbi.nlm.nih.gov/entrez/query.fcgi?db=gene&cmd=Retrieve&dopt=full_report&list_uids=101977394) | Spsb1 | -1.9564 | -3.88092 | 0.001232 | 0.040707 |
| [101954857](https://www.ncbi.nlm.nih.gov/entrez/query.fcgi?db=gene&cmd=Retrieve&dopt=full_report&list_uids=101954857) | LOC101954857 | -1.96685 | -3.90913 | 1.3E-06 | 0.000191 |
| [101971795](https://www.ncbi.nlm.nih.gov/entrez/query.fcgi?db=gene&cmd=Retrieve&dopt=full_report&list_uids=101971795) | Znf385b | -1.98372 | -3.95512 | 0.001002 | 0.035147 |
| [101977496](https://www.ncbi.nlm.nih.gov/entrez/query.fcgi?db=gene&cmd=Retrieve&dopt=full_report&list_uids=101977496) | Acss3 | -1.98396 | -3.95577 | 8.79E-06 | 0.000914 |
| [101962590](https://www.ncbi.nlm.nih.gov/entrez/query.fcgi?db=gene&cmd=Retrieve&dopt=full_report&list_uids=101962590) | Atf3 | -1.99563 | -3.9879 | 1.99E-06 | 0.000272 |
| [101963637](https://www.ncbi.nlm.nih.gov/entrez/query.fcgi?db=gene&cmd=Retrieve&dopt=full_report&list_uids=101963637) | Per1 | -2.03748 | -4.10529 | 1.76E-07 | 3.23E-05 |
| [101965061](https://www.ncbi.nlm.nih.gov/entrez/query.fcgi?db=gene&cmd=Retrieve&dopt=full_report&list_uids=101965061) | LOC101965061 | -2.04395 | -4.12374 | 0.000321 | 0.014744 |
| [101970643](https://www.ncbi.nlm.nih.gov/entrez/query.fcgi?db=gene&cmd=Retrieve&dopt=full_report&list_uids=101970643) | Otud1 | -2.05437 | -4.15363 | 4.92E-08 | 1.15E-05 |
| [101977815](https://www.ncbi.nlm.nih.gov/entrez/query.fcgi?db=gene&cmd=Retrieve&dopt=full_report&list_uids=101977815) | Max | -2.07117 | -4.20226 | 8.79E-12 | 5.25E-09 |
| [101956443](https://www.ncbi.nlm.nih.gov/entrez/query.fcgi?db=gene&cmd=Retrieve&dopt=full_report&list_uids=101956443) | Rcan1 | -2.07521 | -4.21405 | 3.85E-06 | 0.000469 |
| [101965952](https://www.ncbi.nlm.nih.gov/entrez/query.fcgi?db=gene&cmd=Retrieve&dopt=full_report&list_uids=101965952) | Ccdc6 | -2.08034 | -4.22907 | 3.59E-05 | 0.002755 |
| [101955757](https://www.ncbi.nlm.nih.gov/entrez/query.fcgi?db=gene&cmd=Retrieve&dopt=full_report&list_uids=101955757) | Ass1 | -2.09835 | -4.2822 | 1.08E-05 | 0.001053 |
| [101964669](https://www.ncbi.nlm.nih.gov/entrez/query.fcgi?db=gene&cmd=Retrieve&dopt=full_report&list_uids=101964669) | Hspb1 | -2.09901 | -4.28414 | 4.62E-09 | 1.42E-06 |
| [101973082](https://www.ncbi.nlm.nih.gov/entrez/query.fcgi?db=gene&cmd=Retrieve&dopt=full_report&list_uids=101973082) | Klhl21 | -2.10421 | -4.29961 | 7.36E-10 | 2.93E-07 |
| [101959724](https://www.ncbi.nlm.nih.gov/entrez/query.fcgi?db=gene&cmd=Retrieve&dopt=full_report&list_uids=101959724) | Sesn1 | -2.10531 | -4.3029 | 2.03E-09 | 7.46E-07 |
| [101976734](https://www.ncbi.nlm.nih.gov/entrez/query.fcgi?db=gene&cmd=Retrieve&dopt=full_report&list_uids=101976734) | Ca3 | -2.14305 | -4.41696 | 4.1E-05 | 0.003046 |
| [101966771](https://www.ncbi.nlm.nih.gov/entrez/query.fcgi?db=gene&cmd=Retrieve&dopt=full_report&list_uids=101966771) | LOC101966771 | -2.16156 | -4.47397 | 0.000718 | 0.027326 |
| [101973557](https://www.ncbi.nlm.nih.gov/entrez/query.fcgi?db=gene&cmd=Retrieve&dopt=full_report&list_uids=101973557) | Slc2a13 | -2.16309 | -4.47874 | 0.000889 | 0.032297 |
| [101960341](https://www.ncbi.nlm.nih.gov/entrez/query.fcgi?db=gene&cmd=Retrieve&dopt=full_report&list_uids=101960341) | Pi16 | -2.1714 | -4.50461 | 1.02E-05 | 0.001014 |
| [101955139](https://www.ncbi.nlm.nih.gov/entrez/query.fcgi?db=gene&cmd=Retrieve&dopt=full_report&list_uids=101955139) | Ppm1k | -2.17923 | -4.5291 | 1.83E-05 | 0.001559 |
| [101958148](https://www.ncbi.nlm.nih.gov/entrez/query.fcgi?db=gene&cmd=Retrieve&dopt=full_report&list_uids=101958148) | Ccbl2 | -2.18814 | -4.55716 | 3.71E-07 | 6.34E-05 |
| [101976847](https://www.ncbi.nlm.nih.gov/entrez/query.fcgi?db=gene&cmd=Retrieve&dopt=full_report&list_uids=101976847) | Gpr157 | -2.19901 | -4.59164 | 1.29E-07 | 2.45E-05 |
| [101978068](https://www.ncbi.nlm.nih.gov/entrez/query.fcgi?db=gene&cmd=Retrieve&dopt=full_report&list_uids=101978068) | Rasl11b | -2.22962 | -4.6901 | 2.7E-05 | 0.002167 |
| [101957188](https://www.ncbi.nlm.nih.gov/entrez/query.fcgi?db=gene&cmd=Retrieve&dopt=full_report&list_uids=101957188) | Tnfrsf12a | -2.25283 | -4.76616 | 2.24E-08 | 5.78E-06 |
| [101957372](https://www.ncbi.nlm.nih.gov/entrez/query.fcgi?db=gene&cmd=Retrieve&dopt=full_report&list_uids=101957372) | Fam46c | -2.25726 | -4.78082 | 0.000202 | 0.010858 |
| [101967490](https://www.ncbi.nlm.nih.gov/entrez/query.fcgi?db=gene&cmd=Retrieve&dopt=full_report&list_uids=101967490) | Foxo1 | -2.2857 | -4.87599 | 2.45E-11 | 1.33E-08 |
| [101976372](https://www.ncbi.nlm.nih.gov/entrez/query.fcgi?db=gene&cmd=Retrieve&dopt=full_report&list_uids=101976372) | Col9a1 | -2.29549 | -4.90922 | 1.83E-05 | 0.001559 |
| [101974987](https://www.ncbi.nlm.nih.gov/entrez/query.fcgi?db=gene&cmd=Retrieve&dopt=full_report&list_uids=101974987) | Ip6k3 | -2.3038 | -4.93756 | 2.36E-09 | 8.31E-07 |
| [101958044](https://www.ncbi.nlm.nih.gov/entrez/query.fcgi?db=gene&cmd=Retrieve&dopt=full_report&list_uids=101958044) | Abra | -2.32059 | -4.99536 | 2.55E-10 | 1.09E-07 |
| [101978320](https://www.ncbi.nlm.nih.gov/entrez/query.fcgi?db=gene&cmd=Retrieve&dopt=full_report&list_uids=101978320) | Acss1 | -2.33052 | -5.02986 | 1.45E-11 | 8.27E-09 |
| [101967903](https://www.ncbi.nlm.nih.gov/entrez/query.fcgi?db=gene&cmd=Retrieve&dopt=full_report&list_uids=101967903) | Ak4 | -2.39266 | -5.25124 | 2.17E-07 | 3.88E-05 |
| [101977049](https://www.ncbi.nlm.nih.gov/entrez/query.fcgi?db=gene&cmd=Retrieve&dopt=full_report&list_uids=101977049) | Trim63 | -2.40136 | -5.28301 | 6.14E-08 | 1.38E-05 |
| [101974891](https://www.ncbi.nlm.nih.gov/entrez/query.fcgi?db=gene&cmd=Retrieve&dopt=full_report&list_uids=101974891) | Slc7a8 | -2.41618 | -5.33756 | 3.25E-09 | 1.11E-06 |
| [101978376](https://www.ncbi.nlm.nih.gov/entrez/query.fcgi?db=gene&cmd=Retrieve&dopt=full_report&list_uids=101978376) | Fntb | -2.45083 | -5.46732 | 1.14E-10 | 5.23E-08 |
| [101977672](https://www.ncbi.nlm.nih.gov/entrez/query.fcgi?db=gene&cmd=Retrieve&dopt=full_report&list_uids=101977672) | Slc25a33 | -2.45913 | -5.49885 | 3.08E-11 | 1.6E-08 |
| [106144852](https://www.ncbi.nlm.nih.gov/entrez/query.fcgi?db=gene&cmd=Retrieve&dopt=full_report&list_uids=106144852) | LOC106144852 | -2.48776 | -5.60908 | 3.96E-09 | 1.28E-06 |
| [101967031](https://www.ncbi.nlm.nih.gov/entrez/query.fcgi?db=gene&cmd=Retrieve&dopt=full_report&list_uids=101967031) | Ankrd2 | -2.50365 | -5.67116 | 2.27E-08 | 5.78E-06 |
| [101958805](https://www.ncbi.nlm.nih.gov/entrez/query.fcgi?db=gene&cmd=Retrieve&dopt=full_report&list_uids=101958805) | Csrp3 | -2.50947 | -5.69411 | 1.57E-05 | 0.001409 |
| [101957577](https://www.ncbi.nlm.nih.gov/entrez/query.fcgi?db=gene&cmd=Retrieve&dopt=full_report&list_uids=101957577) | Cflar | -2.5228 | -5.74697 | 6.66E-16 | 1.14E-12 |
| [101963811](https://www.ncbi.nlm.nih.gov/entrez/query.fcgi?db=gene&cmd=Retrieve&dopt=full_report&list_uids=101963811) | Pnrc1 | -2.54134 | -5.82128 | 5.06E-09 | 1.51E-06 |
| [101956173](https://www.ncbi.nlm.nih.gov/entrez/query.fcgi?db=gene&cmd=Retrieve&dopt=full_report&list_uids=101956173) | Ampd3 | -2.54976 | -5.85537 | 1.97E-07 | 3.57E-05 |
| [101975062](https://www.ncbi.nlm.nih.gov/entrez/query.fcgi?db=gene&cmd=Retrieve&dopt=full_report&list_uids=101975062) | Errfi1 | -2.57335 | -5.9519 | 7.48E-11 | 3.58E-08 |
| [101973761](https://www.ncbi.nlm.nih.gov/entrez/query.fcgi?db=gene&cmd=Retrieve&dopt=full_report&list_uids=101973761) | Postn | -2.57735 | -5.96842 | 4.26E-05 | 0.003089 |
| [101959575](https://www.ncbi.nlm.nih.gov/entrez/query.fcgi?db=gene&cmd=Retrieve&dopt=full_report&list_uids=101959575) | Fgd2 | -2.59785 | -6.05382 | 5.37E-07 | 8.8E-05 |
| [101975528](https://www.ncbi.nlm.nih.gov/entrez/query.fcgi?db=gene&cmd=Retrieve&dopt=full_report&list_uids=101975528) | Pitx3 | -2.59803 | -6.05457 | 1.99E-08 | 5.29E-06 |
| [101963246](https://www.ncbi.nlm.nih.gov/entrez/query.fcgi?db=gene&cmd=Retrieve&dopt=full_report&list_uids=101963246) | Sgk1 | -2.60609 | -6.0885 | 2.06E-09 | 7.46E-07 |
| [101965820](https://www.ncbi.nlm.nih.gov/entrez/query.fcgi?db=gene&cmd=Retrieve&dopt=full_report&list_uids=101965820) | Sik1 | -2.60763 | -6.09504 | 1.32E-09 | 5.09E-07 |
| [101961849](https://www.ncbi.nlm.nih.gov/entrez/query.fcgi?db=gene&cmd=Retrieve&dopt=full_report&list_uids=101961849) | Itprip | -2.62159 | -6.15428 | 1E-06 | 0.000156 |
| [101970884](https://www.ncbi.nlm.nih.gov/entrez/query.fcgi?db=gene&cmd=Retrieve&dopt=full_report&list_uids=101970884) | Glul | -2.64081 | -6.23682 | 2.72E-12 | 1.91E-09 |
| [101976907](https://www.ncbi.nlm.nih.gov/entrez/query.fcgi?db=gene&cmd=Retrieve&dopt=full_report&list_uids=101976907) | Mical2 | -2.64127 | -6.23879 | 3.17E-12 | 2.11E-09 |
| [101977121](https://www.ncbi.nlm.nih.gov/entrez/query.fcgi?db=gene&cmd=Retrieve&dopt=full_report&list_uids=101977121) | Mcph1 | -2.68965 | -6.45156 | 1.28E-05 | 0.001195 |
| [101956852](https://www.ncbi.nlm.nih.gov/entrez/query.fcgi?db=gene&cmd=Retrieve&dopt=full_report&list_uids=101956852) | Oxct1 | -2.71699 | -6.57501 | 3.15E-10 | 1.3E-07 |
| [101969382](https://www.ncbi.nlm.nih.gov/entrez/query.fcgi?db=gene&cmd=Retrieve&dopt=full_report&list_uids=101969382) | LOC101969382 | -2.77803 | -6.85917 | 4.44E-15 | 5.31E-12 |
| [101972052](https://www.ncbi.nlm.nih.gov/entrez/query.fcgi?db=gene&cmd=Retrieve&dopt=full_report&list_uids=101972052) | Vwa3b | -2.79417 | -6.9363 | 1.16E-07 | 2.25E-05 |
| [101956400](https://www.ncbi.nlm.nih.gov/entrez/query.fcgi?db=gene&cmd=Retrieve&dopt=full_report&list_uids=101956400) | LOC101956400 | -2.79883 | -6.95875 | 1.33E-15 | 1.99E-12 |
| [101968562](https://www.ncbi.nlm.nih.gov/entrez/query.fcgi?db=gene&cmd=Retrieve&dopt=full_report&list_uids=101968562) | Adamtsl2 | -2.82809 | -7.10135 | 9.04E-06 | 0.000917 |
| [101973490](https://www.ncbi.nlm.nih.gov/entrez/query.fcgi?db=gene&cmd=Retrieve&dopt=full_report&list_uids=101973490) | Abcb4 | -2.84855 | -7.20275 | 3.9E-07 | 6.57E-05 |
| [101961989](https://www.ncbi.nlm.nih.gov/entrez/query.fcgi?db=gene&cmd=Retrieve&dopt=full_report&list_uids=101961989) | Tsc22d3 | -2.90359 | -7.48288 | 6.44E-15 | 6.42E-12 |
| [101975126](https://www.ncbi.nlm.nih.gov/entrez/query.fcgi?db=gene&cmd=Retrieve&dopt=full_report&list_uids=101975126) | LOC101975126 | -2.90737 | -7.50247 | 1.66E-05 | 0.001451 |
| [101969148](https://www.ncbi.nlm.nih.gov/entrez/query.fcgi?db=gene&cmd=Retrieve&dopt=full_report&list_uids=101969148) | LOC101969148 | -2.9138 | -7.53602 | 4.45E-09 | 1.4E-06 |
| [101961786](https://www.ncbi.nlm.nih.gov/entrez/query.fcgi?db=gene&cmd=Retrieve&dopt=full_report&list_uids=101961786) | LOC101961786 | -2.94656 | -7.70911 | 0.000544 | 0.022118 |
| [101964964](https://www.ncbi.nlm.nih.gov/entrez/query.fcgi?db=gene&cmd=Retrieve&dopt=full_report&list_uids=101964964) | Btg2 | -2.99076 | -7.94892 | 7.3E-08 | 1.56E-05 |
| [101964310](https://www.ncbi.nlm.nih.gov/entrez/query.fcgi?db=gene&cmd=Retrieve&dopt=full_report&list_uids=101964310) | LOC101964310 | -3.00787 | -8.04378 | 0.000276 | 0.013284 |
| [101963592](https://www.ncbi.nlm.nih.gov/entrez/query.fcgi?db=gene&cmd=Retrieve&dopt=full_report&list_uids=101963592) | Klf15 | -3.07493 | -8.42648 | 6.05E-09 | 1.76E-06 |
| [101969717](https://www.ncbi.nlm.nih.gov/entrez/query.fcgi?db=gene&cmd=Retrieve&dopt=full_report&list_uids=101969717) | Mettl21c | -3.2881 | -9.76824 | 1.33E-06 | 0.000191 |
| [101967428](https://www.ncbi.nlm.nih.gov/entrez/query.fcgi?db=gene&cmd=Retrieve&dopt=full_report&list_uids=101967428) | Fkbp5 | -3.41462 | -10.6636 | 5.55E-16 | 1.11E-12 |
| [101955057](https://www.ncbi.nlm.nih.gov/entrez/query.fcgi?db=gene&cmd=Retrieve&dopt=full_report&list_uids=101955057) | Idi2 | -3.4166 | -10.6783 | 1.01E-12 | 8.02E-10 |
| [101964142](https://www.ncbi.nlm.nih.gov/entrez/query.fcgi?db=gene&cmd=Retrieve&dopt=full_report&list_uids=101964142) | Chac1 | -3.55353 | -11.7414 | 9.01E-07 | 0.000144 |
| [101967306](https://www.ncbi.nlm.nih.gov/entrez/query.fcgi?db=gene&cmd=Retrieve&dopt=full_report&list_uids=101967306) | Cebpd | -3.66822 | -12.7129 | 0 | 0 |
| [101967141](https://www.ncbi.nlm.nih.gov/entrez/query.fcgi?db=gene&cmd=Retrieve&dopt=full_report&list_uids=101967141) | LOC101967141 | -3.76625 | -13.6068 | 3.36E-13 | 2.87E-10 |
| [101969412](https://www.ncbi.nlm.nih.gov/entrez/query.fcgi?db=gene&cmd=Retrieve&dopt=full_report&list_uids=101969412) | LOC101969412 | -3.81473 | -14.0717 | 0 | 0 |
| [101965267](https://www.ncbi.nlm.nih.gov/entrez/query.fcgi?db=gene&cmd=Retrieve&dopt=full_report&list_uids=101965267) | Arrdc2 | -3.8393 | -14.3134 | 5.22E-14 | 4.8E-11 |
| [101956126](https://www.ncbi.nlm.nih.gov/entrez/query.fcgi?db=gene&cmd=Retrieve&dopt=full_report&list_uids=101956126) | Hspa1a | -4.02706 | -16.3029 | 0 | 0 |
| [101956579](https://www.ncbi.nlm.nih.gov/entrez/query.fcgi?db=gene&cmd=Retrieve&dopt=full_report&list_uids=101956579) | Ybx2 | -4.06518 | -16.7394 | 3.22E-15 | 4.28E-12 |
| [101975661](https://www.ncbi.nlm.nih.gov/entrez/query.fcgi?db=gene&cmd=Retrieve&dopt=full_report&list_uids=101975661) | Nfil3 | -4.15665 | -17.8352 | 3.9E-11 | 1.94E-08 |
| [101955831](https://www.ncbi.nlm.nih.gov/entrez/query.fcgi?db=gene&cmd=Retrieve&dopt=full_report&list_uids=101955831) | Hspa1b | -4.25074 | -19.0371 | 0 | 0 |
| [101975221](https://www.ncbi.nlm.nih.gov/entrez/query.fcgi?db=gene&cmd=Retrieve&dopt=full_report&list_uids=101975221) | Ddit4 | -5.04074 | -32.9166 | 0 | 0 |
| **LT vs. SA** |  |  |  |  |  |
| [101965856](https://www.ncbi.nlm.nih.gov/entrez/query.fcgi?db=gene&cmd=Retrieve&dopt=full_report&list_uids=101965856) | LOC101965856 | 10.01498 | 1034.686 | 0 | 0 |
| [101978243](https://www.ncbi.nlm.nih.gov/entrez/query.fcgi?db=gene&cmd=Retrieve&dopt=full_report&list_uids=101978243) | Itih3 | 9.178185 | 579.3071 | 0 | 0 |
| [101975693](https://www.ncbi.nlm.nih.gov/entrez/query.fcgi?db=gene&cmd=Retrieve&dopt=full_report&list_uids=101975693) | Aldob | 8.110734 | 276.423 | 0 | 0 |
| [101970551](https://www.ncbi.nlm.nih.gov/entrez/query.fcgi?db=gene&cmd=Retrieve&dopt=full_report&list_uids=101970551) | Tf | 7.246811 | 151.8824 | 1.11E-16 | 9.58E-14 |
| [101970831](https://www.ncbi.nlm.nih.gov/entrez/query.fcgi?db=gene&cmd=Retrieve&dopt=full_report&list_uids=101970831) | LOC101970831 | 7.241901 | 151.3663 | 0 | 0 |
| [101962170](https://www.ncbi.nlm.nih.gov/entrez/query.fcgi?db=gene&cmd=Retrieve&dopt=full_report&list_uids=101962170) | Hpx | 6.567213 | 94.82614 | 4.44E-16 | 3.58E-13 |
| [101954486](https://www.ncbi.nlm.nih.gov/entrez/query.fcgi?db=gene&cmd=Retrieve&dopt=full_report&list_uids=101954486) | Itih4 | 6.133022 | 70.18164 | 1.11E-16 | 9.58E-14 |
| [101963717](https://www.ncbi.nlm.nih.gov/entrez/query.fcgi?db=gene&cmd=Retrieve&dopt=full_report&list_uids=101963717) | Cfi | 5.842113 | 57.36555 | 1.7E-12 | 6.43E-10 |
| [101973439](https://www.ncbi.nlm.nih.gov/entrez/query.fcgi?db=gene&cmd=Retrieve&dopt=full_report&list_uids=101973439) | LOC101973439 | 4.740071 | 26.72412 | 4.07E-12 | 1.41E-09 |
| [101958459](https://www.ncbi.nlm.nih.gov/entrez/query.fcgi?db=gene&cmd=Retrieve&dopt=full_report&list_uids=101958459) | Serpina5 | 4.649943 | 25.1057 | 5.19E-13 | 2.51E-10 |
| [101967403](https://www.ncbi.nlm.nih.gov/entrez/query.fcgi?db=gene&cmd=Retrieve&dopt=full_report&list_uids=101967403) | F12 | 4.604221 | 24.32253 | 2.72E-08 | 3.97E-06 |
| [101969507](https://www.ncbi.nlm.nih.gov/entrez/query.fcgi?db=gene&cmd=Retrieve&dopt=full_report&list_uids=101969507) | Fbp1 | 4.572849 | 23.79934 | 1.18E-09 | 2.19E-07 |
| [101963163](https://www.ncbi.nlm.nih.gov/entrez/query.fcgi?db=gene&cmd=Retrieve&dopt=full_report&list_uids=101963163) | Slc25a47 | 4.536258 | 23.2033 | 4.28E-10 | 9.58E-08 |
| [101977232](https://www.ncbi.nlm.nih.gov/entrez/query.fcgi?db=gene&cmd=Retrieve&dopt=full_report&list_uids=101977232) | Cfb | 4.350993 | 20.40701 | 4.51E-09 | 7.68E-07 |
| [101975171](https://www.ncbi.nlm.nih.gov/entrez/query.fcgi?db=gene&cmd=Retrieve&dopt=full_report&list_uids=101975171) | LOC101975171 | 4.328605 | 20.09278 | 7.31E-10 | 1.47E-07 |
| [101959820](https://www.ncbi.nlm.nih.gov/entrez/query.fcgi?db=gene&cmd=Retrieve&dopt=full_report&list_uids=101959820) | Vtn | 4.188543 | 18.23379 | 1.17E-10 | 3.07E-08 |
| [101972730](https://www.ncbi.nlm.nih.gov/entrez/query.fcgi?db=gene&cmd=Retrieve&dopt=full_report&list_uids=101972730) | Ncapg | 4.021427 | 16.23941 | 3.63E-08 | 5.1E-06 |
| [101965332](https://www.ncbi.nlm.nih.gov/entrez/query.fcgi?db=gene&cmd=Retrieve&dopt=full_report&list_uids=101965332) | LOC101965332 | 3.921484 | 15.1525 | 5.34E-10 | 1.14E-07 |
| [101965561](https://www.ncbi.nlm.nih.gov/entrez/query.fcgi?db=gene&cmd=Retrieve&dopt=full_report&list_uids=101965561) | Plk5 | 3.885625 | 14.78052 | 3.86E-11 | 1.14E-08 |
| [101955377](https://www.ncbi.nlm.nih.gov/entrez/query.fcgi?db=gene&cmd=Retrieve&dopt=full_report&list_uids=101955377) | Rgn | 3.85373 | 14.45734 | 1.2E-09 | 2.2E-07 |
| [101975452](https://www.ncbi.nlm.nih.gov/entrez/query.fcgi?db=gene&cmd=Retrieve&dopt=full_report&list_uids=101975452) | LOC101975452 | 3.843915 | 14.35931 | 8.36E-10 | 1.63E-07 |
| [101963149](https://www.ncbi.nlm.nih.gov/entrez/query.fcgi?db=gene&cmd=Retrieve&dopt=full_report&list_uids=101963149) | Igfn1 | 3.688658 | 12.89427 | 6.75E-14 | 3.88E-11 |
| [101976500](https://www.ncbi.nlm.nih.gov/entrez/query.fcgi?db=gene&cmd=Retrieve&dopt=full_report&list_uids=101976500) | Hp | 3.674416 | 12.7676 | 1.56E-06 | 0.000142 |
| [101971136](https://www.ncbi.nlm.nih.gov/entrez/query.fcgi?db=gene&cmd=Retrieve&dopt=full_report&list_uids=101971136) | Pklr | 3.633604 | 12.41149 | 6.98E-07 | 7.09E-05 |
| [101966057](https://www.ncbi.nlm.nih.gov/entrez/query.fcgi?db=gene&cmd=Retrieve&dopt=full_report&list_uids=101966057) | Rbp4 | 3.576941 | 11.93347 | 1.36E-07 | 1.69E-05 |
| [101961664](https://www.ncbi.nlm.nih.gov/entrez/query.fcgi?db=gene&cmd=Retrieve&dopt=full_report&list_uids=101961664) | Apoe | 3.560069 | 11.79472 | 9.99E-10 | 1.89E-07 |
| [101967464](https://www.ncbi.nlm.nih.gov/entrez/query.fcgi?db=gene&cmd=Retrieve&dopt=full_report&list_uids=101967464) | LOC101967464 | 3.526081 | 11.52009 | 8.96E-09 | 1.46E-06 |
| [101977160](https://www.ncbi.nlm.nih.gov/entrez/query.fcgi?db=gene&cmd=Retrieve&dopt=full_report&list_uids=101977160) | LOC101977160 | 3.48728 | 11.2144 | 5.11E-06 | 0.000394 |
| [101968287](https://www.ncbi.nlm.nih.gov/entrez/query.fcgi?db=gene&cmd=Retrieve&dopt=full_report&list_uids=101968287) | Pck2 | 3.273209 | 9.667944 | 3.38E-07 | 3.72E-05 |
| [101956719](https://www.ncbi.nlm.nih.gov/entrez/query.fcgi?db=gene&cmd=Retrieve&dopt=full_report&list_uids=101956719) | Sec16b | 3.267115 | 9.62719 | 4.36E-06 | 0.000354 |
| [101974020](https://www.ncbi.nlm.nih.gov/entrez/query.fcgi?db=gene&cmd=Retrieve&dopt=full_report&list_uids=101974020) | Myh3 | 3.24855 | 9.504102 | 2.95E-07 | 3.34E-05 |
| [101967402](https://www.ncbi.nlm.nih.gov/entrez/query.fcgi?db=gene&cmd=Retrieve&dopt=full_report&list_uids=101967402) | LOC101967402 | 3.22867 | 9.374032 | 4.09E-07 | 4.45E-05 |
| [101962133](https://www.ncbi.nlm.nih.gov/entrez/query.fcgi?db=gene&cmd=Retrieve&dopt=full_report&list_uids=101962133) | Gls2 | 3.205526 | 9.224856 | 2.65E-09 | 4.65E-07 |
| [101963934](https://www.ncbi.nlm.nih.gov/entrez/query.fcgi?db=gene&cmd=Retrieve&dopt=full_report&list_uids=101963934) | Sfrp5 | 3.182027 | 9.075813 | 1.02E-08 | 1.64E-06 |
| [101974890](https://www.ncbi.nlm.nih.gov/entrez/query.fcgi?db=gene&cmd=Retrieve&dopt=full_report&list_uids=101974890) | LOC101974890 | 3.114677 | 8.661864 | 1.52E-07 | 1.84E-05 |
| [101966983](https://www.ncbi.nlm.nih.gov/entrez/query.fcgi?db=gene&cmd=Retrieve&dopt=full_report&list_uids=101966983) | Sdc1 | 3.083872 | 8.478869 | 7.41E-05 | 0.003551 |
| [101966104](https://www.ncbi.nlm.nih.gov/entrez/query.fcgi?db=gene&cmd=Retrieve&dopt=full_report&list_uids=101966104) | Ccl16 | 3.00111 | 8.006156 | 2.78E-05 | 0.001587 |
| [101962704](https://www.ncbi.nlm.nih.gov/entrez/query.fcgi?db=gene&cmd=Retrieve&dopt=full_report&list_uids=101962704) | Loxl4 | 2.962338 | 7.79386 | 4.3E-07 | 4.6E-05 |
| [101964363](https://www.ncbi.nlm.nih.gov/entrez/query.fcgi?db=gene&cmd=Retrieve&dopt=full_report&list_uids=101964363) | Acat2 | 2.962041 | 7.792253 | 1.13E-08 | 1.8E-06 |
| [101972152](https://www.ncbi.nlm.nih.gov/entrez/query.fcgi?db=gene&cmd=Retrieve&dopt=full_report&list_uids=101972152) | LOC101972152 | 2.954357 | 7.750864 | 2.09E-07 | 2.47E-05 |
| [101973237](https://www.ncbi.nlm.nih.gov/entrez/query.fcgi?db=gene&cmd=Retrieve&dopt=full_report&list_uids=101973237) | Krt18 | 2.90707 | 7.500935 | 8.24E-06 | 0.000569 |
| [101967759](https://www.ncbi.nlm.nih.gov/entrez/query.fcgi?db=gene&cmd=Retrieve&dopt=full_report&list_uids=101967759) | Galm | 2.905461 | 7.49257 | 4.53E-07 | 4.76E-05 |
| [101973144](https://www.ncbi.nlm.nih.gov/entrez/query.fcgi?db=gene&cmd=Retrieve&dopt=full_report&list_uids=101973144) | Crym | 2.844401 | 7.182077 | 2.34E-06 | 0.000201 |
| [101971217](https://www.ncbi.nlm.nih.gov/entrez/query.fcgi?db=gene&cmd=Retrieve&dopt=full_report&list_uids=101971217) | Msrb2 | 2.800444 | 6.966549 | 5.15E-07 | 5.37E-05 |
| [101963917](https://www.ncbi.nlm.nih.gov/entrez/query.fcgi?db=gene&cmd=Retrieve&dopt=full_report&list_uids=101963917) | Paqr9 | 2.770467 | 6.823288 | 1.16E-11 | 3.8E-09 |
| [101955669](https://www.ncbi.nlm.nih.gov/entrez/query.fcgi?db=gene&cmd=Retrieve&dopt=full_report&list_uids=101955669) | LOC101955669 | 2.737801 | 6.670526 | 1.81E-06 | 0.000161 |
| [101961465](https://www.ncbi.nlm.nih.gov/entrez/query.fcgi?db=gene&cmd=Retrieve&dopt=full_report&list_uids=101961465) | Hmgcs2 | 2.651049 | 6.281239 | 0.000127 | 0.005546 |
| [101959731](https://www.ncbi.nlm.nih.gov/entrez/query.fcgi?db=gene&cmd=Retrieve&dopt=full_report&list_uids=101959731) | C3 | 2.614474 | 6.123997 | 4.32E-06 | 0.000353 |
| [101968376](https://www.ncbi.nlm.nih.gov/entrez/query.fcgi?db=gene&cmd=Retrieve&dopt=full_report&list_uids=101968376) | Gpld1 | 2.584741 | 5.999077 | 0.00018 | 0.007131 |
| [101963016](https://www.ncbi.nlm.nih.gov/entrez/query.fcgi?db=gene&cmd=Retrieve&dopt=full_report&list_uids=101963016) | Col18a1 | 2.532947 | 5.787528 | 4.75E-06 | 0.000375 |
| [101966269](https://www.ncbi.nlm.nih.gov/entrez/query.fcgi?db=gene&cmd=Retrieve&dopt=full_report&list_uids=101966269) | Dnase1l3 | 2.518165 | 5.728529 | 0.000107 | 0.004855 |
| [101964067](https://www.ncbi.nlm.nih.gov/entrez/query.fcgi?db=gene&cmd=Retrieve&dopt=full_report&list_uids=101964067) | Esrp2 | 2.469997 | 5.540426 | 0.000178 | 0.007118 |
| [101975635](https://www.ncbi.nlm.nih.gov/entrez/query.fcgi?db=gene&cmd=Retrieve&dopt=full_report&list_uids=101975635) | LOC101975635 | 2.420482 | 5.353498 | 1.01E-06 | 9.62E-05 |
| [101961005](https://www.ncbi.nlm.nih.gov/entrez/query.fcgi?db=gene&cmd=Retrieve&dopt=full_report&list_uids=101961005) | Pros1 | 2.398761 | 5.273501 | 5.39E-06 | 0.000412 |
| [101973579](https://www.ncbi.nlm.nih.gov/entrez/query.fcgi?db=gene&cmd=Retrieve&dopt=full_report&list_uids=101973579) | LOC101973579 | 2.365127 | 5.151979 | 5.94E-08 | 7.98E-06 |
| [101969545](https://www.ncbi.nlm.nih.gov/entrez/query.fcgi?db=gene&cmd=Retrieve&dopt=full_report&list_uids=101969545) | Rgs16 | 2.324503 | 5.008933 | 0.000108 | 0.004888 |
| [101972738](https://www.ncbi.nlm.nih.gov/entrez/query.fcgi?db=gene&cmd=Retrieve&dopt=full_report&list_uids=101972738) | Anpep | 2.305665 | 4.943954 | 7.27E-06 | 0.000517 |
| [101969970](https://www.ncbi.nlm.nih.gov/entrez/query.fcgi?db=gene&cmd=Retrieve&dopt=full_report&list_uids=101969970) | Perp | 2.300959 | 4.927854 | 1.19E-05 | 0.000771 |
| [101974575](https://www.ncbi.nlm.nih.gov/entrez/query.fcgi?db=gene&cmd=Retrieve&dopt=full_report&list_uids=101974575) | Lrg1 | 2.285432 | 4.875099 | 3.26E-07 | 3.65E-05 |
| [101971053](https://www.ncbi.nlm.nih.gov/entrez/query.fcgi?db=gene&cmd=Retrieve&dopt=full_report&list_uids=101971053) | Sfxn2 | 2.27039 | 4.824535 | 0.002439 | 0.047722 |
| [101957255](https://www.ncbi.nlm.nih.gov/entrez/query.fcgi?db=gene&cmd=Retrieve&dopt=full_report&list_uids=101957255) | Atp7b | 2.256633 | 4.778748 | 1.06E-06 | 0.0001 |
| [101970238](https://www.ncbi.nlm.nih.gov/entrez/query.fcgi?db=gene&cmd=Retrieve&dopt=full_report&list_uids=101970238) | Pygl | 2.210772 | 4.629228 | 7.67E-08 | 1.01E-05 |
| [101959551](https://www.ncbi.nlm.nih.gov/entrez/query.fcgi?db=gene&cmd=Retrieve&dopt=full_report&list_uids=101959551) | Ncam1 | 2.191245 | 4.566995 | 3.04E-10 | 7.2E-08 |
| [101956675](https://www.ncbi.nlm.nih.gov/entrez/query.fcgi?db=gene&cmd=Retrieve&dopt=full_report&list_uids=101956675) | Fam124b | 2.177935 | 4.525053 | 1.82E-05 | 0.001111 |
| [101956524](https://www.ncbi.nlm.nih.gov/entrez/query.fcgi?db=gene&cmd=Retrieve&dopt=full_report&list_uids=101956524) | Slc25a18 | 2.165398 | 4.485901 | 0.001339 | 0.03211 |
| [101974314](https://www.ncbi.nlm.nih.gov/entrez/query.fcgi?db=gene&cmd=Retrieve&dopt=full_report&list_uids=101974314) | Psat1 | 2.144293 | 4.420755 | 0.000135 | 0.005824 |
| [101973303](https://www.ncbi.nlm.nih.gov/entrez/query.fcgi?db=gene&cmd=Retrieve&dopt=full_report&list_uids=101973303) | Enpp5 | 2.099618 | 4.285958 | 0.000203 | 0.007882 |
| [101966045](https://www.ncbi.nlm.nih.gov/entrez/query.fcgi?db=gene&cmd=Retrieve&dopt=full_report&list_uids=101966045) | LOC101966045 | 2.096543 | 4.276833 | 0.000351 | 0.011885 |
| [101976320](https://www.ncbi.nlm.nih.gov/entrez/query.fcgi?db=gene&cmd=Retrieve&dopt=full_report&list_uids=101976320) | Kcnk3 | 2.095337 | 4.273259 | 1.78E-06 | 0.000159 |
| [101963797](https://www.ncbi.nlm.nih.gov/entrez/query.fcgi?db=gene&cmd=Retrieve&dopt=full_report&list_uids=101963797) | Plekhd1 | 2.090465 | 4.258854 | 0.000839 | 0.022153 |
| [101967654](https://www.ncbi.nlm.nih.gov/entrez/query.fcgi?db=gene&cmd=Retrieve&dopt=full_report&list_uids=101967654) | Thrsp | 2.081641 | 4.232885 | 0.000473 | 0.014821 |
| [101962234](https://www.ncbi.nlm.nih.gov/entrez/query.fcgi?db=gene&cmd=Retrieve&dopt=full_report&list_uids=101962234) | Colq | 2.08158 | 4.232704 | 5.11E-05 | 0.002597 |
| [101978680](https://www.ncbi.nlm.nih.gov/entrez/query.fcgi?db=gene&cmd=Retrieve&dopt=full_report&list_uids=101978680) | Fggy | 2.080237 | 4.228768 | 1.43E-05 | 0.000893 |
| [101954843](https://www.ncbi.nlm.nih.gov/entrez/query.fcgi?db=gene&cmd=Retrieve&dopt=full_report&list_uids=101954843) | Slc6a12 | 2.0586 | 4.165817 | 0.000104 | 0.00477 |
| [101967535](https://www.ncbi.nlm.nih.gov/entrez/query.fcgi?db=gene&cmd=Retrieve&dopt=full_report&list_uids=101967535) | Emb | 2.048246 | 4.136029 | 9.97E-07 | 9.56E-05 |
| [101961794](https://www.ncbi.nlm.nih.gov/entrez/query.fcgi?db=gene&cmd=Retrieve&dopt=full_report&list_uids=101961794) | Eps8l2 | 2.046949 | 4.132312 | 0.000274 | 0.009959 |
| [101974039](https://www.ncbi.nlm.nih.gov/entrez/query.fcgi?db=gene&cmd=Retrieve&dopt=full_report&list_uids=101974039) | Xylb | 2.017166 | 4.047877 | 0.000809 | 0.021729 |
| [101969252](https://www.ncbi.nlm.nih.gov/entrez/query.fcgi?db=gene&cmd=Retrieve&dopt=full_report&list_uids=101969252) | Fam107a | 2.007855 | 4.021839 | 2.46E-05 | 0.001421 |
| [101957238](https://www.ncbi.nlm.nih.gov/entrez/query.fcgi?db=gene&cmd=Retrieve&dopt=full_report&list_uids=101957238) | Hoxd8 | 2.004494 | 4.012478 | 0.000417 | 0.013399 |
| [101974217](https://www.ncbi.nlm.nih.gov/entrez/query.fcgi?db=gene&cmd=Retrieve&dopt=full_report&list_uids=101974217) | Zfr2 | 2.004262 | 4.011836 | 0.00064 | 0.018451 |
| [101964237](https://www.ncbi.nlm.nih.gov/entrez/query.fcgi?db=gene&cmd=Retrieve&dopt=full_report&list_uids=101964237) | Srebf1 | 2.003848 | 4.010682 | 5.59E-06 | 0.000423 |
| [101972974](https://www.ncbi.nlm.nih.gov/entrez/query.fcgi?db=gene&cmd=Retrieve&dopt=full_report&list_uids=101972974) | Mgst1 | 1.985568 | 3.960186 | 1.97E-05 | 0.001181 |
| [101962454](https://www.ncbi.nlm.nih.gov/entrez/query.fcgi?db=gene&cmd=Retrieve&dopt=full_report&list_uids=101962454) | Fam129a | 1.957126 | 3.882878 | 5.04E-10 | 1.11E-07 |
| [101967584](https://www.ncbi.nlm.nih.gov/entrez/query.fcgi?db=gene&cmd=Retrieve&dopt=full_report&list_uids=101967584) | LOC101967584 | 1.939664 | 3.836164 | 8.21E-10 | 1.63E-07 |
| [101971739](https://www.ncbi.nlm.nih.gov/entrez/query.fcgi?db=gene&cmd=Retrieve&dopt=full_report&list_uids=101971739) | LOC101971739 | 1.922607 | 3.791076 | 0.000536 | 0.016271 |
| [101966179](https://www.ncbi.nlm.nih.gov/entrez/query.fcgi?db=gene&cmd=Retrieve&dopt=full_report&list_uids=101966179) | Sord | 1.909672 | 3.757236 | 8.13E-05 | 0.003867 |
| [101955610](https://www.ncbi.nlm.nih.gov/entrez/query.fcgi?db=gene&cmd=Retrieve&dopt=full_report&list_uids=101955610) | Unc5a | 1.907898 | 3.75262 | 0.000776 | 0.02118 |
| [101973974](https://www.ncbi.nlm.nih.gov/entrez/query.fcgi?db=gene&cmd=Retrieve&dopt=full_report&list_uids=101973974) | F5 | 1.900848 | 3.734325 | 0.002356 | 0.04691 |
| [101975511](https://www.ncbi.nlm.nih.gov/entrez/query.fcgi?db=gene&cmd=Retrieve&dopt=full_report&list_uids=101975511) | Bhlhe41 | 1.897468 | 3.725588 | 3.85E-11 | 1.14E-08 |
| [101975580](https://www.ncbi.nlm.nih.gov/entrez/query.fcgi?db=gene&cmd=Retrieve&dopt=full_report&list_uids=101975580) | Igfbp4 | 1.872043 | 3.660507 | 4.44E-06 | 0.000355 |
| [101957914](https://www.ncbi.nlm.nih.gov/entrez/query.fcgi?db=gene&cmd=Retrieve&dopt=full_report&list_uids=101957914) | Iqgap2 | 1.854491 | 3.616241 | 0.00073 | 0.020217 |
| [101971210](https://www.ncbi.nlm.nih.gov/entrez/query.fcgi?db=gene&cmd=Retrieve&dopt=full_report&list_uids=101971210) | Map3k7cl | 1.839907 | 3.57987 | 6.58E-06 | 0.000479 |
| [101967240](https://www.ncbi.nlm.nih.gov/entrez/query.fcgi?db=gene&cmd=Retrieve&dopt=full_report&list_uids=101967240) | Fah | 1.835266 | 3.568372 | 0.000213 | 0.008202 |
| [101974266](https://www.ncbi.nlm.nih.gov/entrez/query.fcgi?db=gene&cmd=Retrieve&dopt=full_report&list_uids=101974266) | LOC101974266 | 1.833608 | 3.564274 | 0.000295 | 0.010468 |
| [101959312](https://www.ncbi.nlm.nih.gov/entrez/query.fcgi?db=gene&cmd=Retrieve&dopt=full_report&list_uids=101959312) | Ndrg2 | 1.823181 | 3.538605 | 1.19E-06 | 0.000111 |
| [101971234](https://www.ncbi.nlm.nih.gov/entrez/query.fcgi?db=gene&cmd=Retrieve&dopt=full_report&list_uids=101971234) | Hsd11b1 | 1.820262 | 3.531453 | 3.34E-05 | 0.001879 |
| [101975450](https://www.ncbi.nlm.nih.gov/entrez/query.fcgi?db=gene&cmd=Retrieve&dopt=full_report&list_uids=101975450) | Tnfaip8 | 1.814293 | 3.516873 | 0.000221 | 0.008419 |
| [101965656](https://www.ncbi.nlm.nih.gov/entrez/query.fcgi?db=gene&cmd=Retrieve&dopt=full_report&list_uids=101965656) | Slc16a11 | 1.806153 | 3.497085 | 0.000114 | 0.005083 |
| [101960796](https://www.ncbi.nlm.nih.gov/entrez/query.fcgi?db=gene&cmd=Retrieve&dopt=full_report&list_uids=101960796) | Sema4g | 1.803258 | 3.490076 | 0.001944 | 0.041276 |
| [101977590](https://www.ncbi.nlm.nih.gov/entrez/query.fcgi?db=gene&cmd=Retrieve&dopt=full_report&list_uids=101977590) | Fzd10 | 1.788488 | 3.454528 | 1.7E-07 | 2.04E-05 |
| [101961301](https://www.ncbi.nlm.nih.gov/entrez/query.fcgi?db=gene&cmd=Retrieve&dopt=full_report&list_uids=101961301) | Lrrc38 | 1.776131 | 3.425065 | 0.00195 | 0.041276 |
| [101969691](https://www.ncbi.nlm.nih.gov/entrez/query.fcgi?db=gene&cmd=Retrieve&dopt=full_report&list_uids=101969691) | Tnfaip3 | 1.771334 | 3.413694 | 1.58E-05 | 0.000975 |
| [101963894](https://www.ncbi.nlm.nih.gov/entrez/query.fcgi?db=gene&cmd=Retrieve&dopt=full_report&list_uids=101963894) | Dact1 | 1.767617 | 3.40491 | 0.000285 | 0.010256 |
| [101956027](https://www.ncbi.nlm.nih.gov/entrez/query.fcgi?db=gene&cmd=Retrieve&dopt=full_report&list_uids=101956027) | Ptprf | 1.761146 | 3.389673 | 0.000636 | 0.018431 |
| [101962344](https://www.ncbi.nlm.nih.gov/entrez/query.fcgi?db=gene&cmd=Retrieve&dopt=full_report&list_uids=101962344) | Slc7a1 | 1.753685 | 3.372188 | 9.64E-06 | 0.000647 |
| [101960513](https://www.ncbi.nlm.nih.gov/entrez/query.fcgi?db=gene&cmd=Retrieve&dopt=full_report&list_uids=101960513) | Dpys | 1.739221 | 3.338548 | 0.001076 | 0.026928 |
| [101977057](https://www.ncbi.nlm.nih.gov/entrez/query.fcgi?db=gene&cmd=Retrieve&dopt=full_report&list_uids=101977057) | Spon2 | 1.73879 | 3.33755 | 0.000492 | 0.015167 |
| [101957080](https://www.ncbi.nlm.nih.gov/entrez/query.fcgi?db=gene&cmd=Retrieve&dopt=full_report&list_uids=101957080) | Mettl7b | 1.738096 | 3.335945 | 0.001972 | 0.041579 |
| [101957431](https://www.ncbi.nlm.nih.gov/entrez/query.fcgi?db=gene&cmd=Retrieve&dopt=full_report&list_uids=101957431) | LOC101957431 | 1.737326 | 3.334166 | 0.000526 | 0.016021 |
| [101956165](https://www.ncbi.nlm.nih.gov/entrez/query.fcgi?db=gene&cmd=Retrieve&dopt=full_report&list_uids=101956165) | Slc1a1 | 1.725766 | 3.307556 | 0.000731 | 0.020217 |
| [101955132](https://www.ncbi.nlm.nih.gov/entrez/query.fcgi?db=gene&cmd=Retrieve&dopt=full_report&list_uids=101955132) | LOC101955132 | 1.723117 | 3.30149 | 4.38E-05 | 0.002333 |
| [101973558](https://www.ncbi.nlm.nih.gov/entrez/query.fcgi?db=gene&cmd=Retrieve&dopt=full_report&list_uids=101973558) | Sfxn1 | 1.717691 | 3.289096 | 6.38E-05 | 0.003147 |
| [101965878](https://www.ncbi.nlm.nih.gov/entrez/query.fcgi?db=gene&cmd=Retrieve&dopt=full_report&list_uids=101965878) | LOC101965878 | 1.702947 | 3.255654 | 1.13E-05 | 0.000738 |
| [101961105](https://www.ncbi.nlm.nih.gov/entrez/query.fcgi?db=gene&cmd=Retrieve&dopt=full_report&list_uids=101961105) | Ncaph | 1.698395 | 3.245397 | 0.001375 | 0.032528 |
| [101963835](https://www.ncbi.nlm.nih.gov/entrez/query.fcgi?db=gene&cmd=Retrieve&dopt=full_report&list_uids=101963835) | Cpt1a | 1.698197 | 3.244951 | 3.31E-07 | 3.67E-05 |
| [101971285](https://www.ncbi.nlm.nih.gov/entrez/query.fcgi?db=gene&cmd=Retrieve&dopt=full_report&list_uids=101971285) | Dhcr7 | 1.684145 | 3.213499 | 0.002019 | 0.04222 |
| [101954279](https://www.ncbi.nlm.nih.gov/entrez/query.fcgi?db=gene&cmd=Retrieve&dopt=full_report&list_uids=101954279) | Dact3 | 1.680086 | 3.20447 | 0.000435 | 0.013884 |
| [101971546](https://www.ncbi.nlm.nih.gov/entrez/query.fcgi?db=gene&cmd=Retrieve&dopt=full_report&list_uids=101971546) | LOC101971546 | 1.670464 | 3.183169 | 0.000462 | 0.014625 |
| [101963574](https://www.ncbi.nlm.nih.gov/entrez/query.fcgi?db=gene&cmd=Retrieve&dopt=full_report&list_uids=101963574) | Apom | 1.669351 | 3.180716 | 0.000538 | 0.016293 |
| [101957760](https://www.ncbi.nlm.nih.gov/entrez/query.fcgi?db=gene&cmd=Retrieve&dopt=full_report&list_uids=101957760) | LOC101957760 | 1.654607 | 3.148374 | 7.54E-05 | 0.003602 |
| [101970763](https://www.ncbi.nlm.nih.gov/entrez/query.fcgi?db=gene&cmd=Retrieve&dopt=full_report&list_uids=101970763) | Ocel1 | 1.651745 | 3.142135 | 0.000281 | 0.010201 |
| [101975773](https://www.ncbi.nlm.nih.gov/entrez/query.fcgi?db=gene&cmd=Retrieve&dopt=full_report&list_uids=101975773) | Dach1 | 1.650428 | 3.139267 | 0.000806 | 0.021702 |
| [101977189](https://www.ncbi.nlm.nih.gov/entrez/query.fcgi?db=gene&cmd=Retrieve&dopt=full_report&list_uids=101977189) | Ca2 | 1.649942 | 3.13821 | 2.48E-08 | 3.69E-06 |
| [101973739](https://www.ncbi.nlm.nih.gov/entrez/query.fcgi?db=gene&cmd=Retrieve&dopt=full_report&list_uids=101973739) | Lgi4 | 1.642385 | 3.121815 | 0.000339 | 0.011631 |
| [101961570](https://www.ncbi.nlm.nih.gov/entrez/query.fcgi?db=gene&cmd=Retrieve&dopt=full_report&list_uids=101961570) | Ahnak2 | 1.633734 | 3.103152 | 0.000384 | 0.012704 |
| [101972067](https://www.ncbi.nlm.nih.gov/entrez/query.fcgi?db=gene&cmd=Retrieve&dopt=full_report&list_uids=101972067) | Nr1d1 | 1.597419 | 3.026015 | 3.98E-05 | 0.002145 |
| [101974275](https://www.ncbi.nlm.nih.gov/entrez/query.fcgi?db=gene&cmd=Retrieve&dopt=full_report&list_uids=101974275) | Maob | 1.596419 | 3.023919 | 9.55E-06 | 0.000644 |
| [101966585](https://www.ncbi.nlm.nih.gov/entrez/query.fcgi?db=gene&cmd=Retrieve&dopt=full_report&list_uids=101966585) | Ak8 | 1.592199 | 3.015086 | 0.002462 | 0.047722 |
| [101971345](https://www.ncbi.nlm.nih.gov/entrez/query.fcgi?db=gene&cmd=Retrieve&dopt=full_report&list_uids=101971345) | Jchain | 1.58972 | 3.009909 | 0.000263 | 0.009737 |
| [101963060](https://www.ncbi.nlm.nih.gov/entrez/query.fcgi?db=gene&cmd=Retrieve&dopt=full_report&list_uids=101963060) | LOC101963060 | 1.588296 | 3.006939 | 0.000658 | 0.018847 |
| [101971489](https://www.ncbi.nlm.nih.gov/entrez/query.fcgi?db=gene&cmd=Retrieve&dopt=full_report&list_uids=101971489) | Tbx6 | 1.577014 | 2.983518 | 0.001705 | 0.037609 |
| [101960521](https://www.ncbi.nlm.nih.gov/entrez/query.fcgi?db=gene&cmd=Retrieve&dopt=full_report&list_uids=101960521) | Hes1 | 1.569372 | 2.967754 | 3.08E-05 | 0.001748 |
| [101956908](https://www.ncbi.nlm.nih.gov/entrez/query.fcgi?db=gene&cmd=Retrieve&dopt=full_report&list_uids=101956908) | Vwa5a | 1.557252 | 2.942928 | 0.000174 | 0.007102 |
| [101969521](https://www.ncbi.nlm.nih.gov/entrez/query.fcgi?db=gene&cmd=Retrieve&dopt=full_report&list_uids=101969521) | Lrrc16a | 1.538933 | 2.905795 | 0.000714 | 0.01992 |
| [101967952](https://www.ncbi.nlm.nih.gov/entrez/query.fcgi?db=gene&cmd=Retrieve&dopt=full_report&list_uids=101967952) | Znf503 | 1.517894 | 2.863728 | 0.000194 | 0.007579 |
| [101973305](https://www.ncbi.nlm.nih.gov/entrez/query.fcgi?db=gene&cmd=Retrieve&dopt=full_report&list_uids=101973305) | Fam184b | 1.503477 | 2.835251 | 0.001984 | 0.041632 |
| [101968312](https://www.ncbi.nlm.nih.gov/entrez/query.fcgi?db=gene&cmd=Retrieve&dopt=full_report&list_uids=101968312) | Ttc32 | 1.501684 | 2.831731 | 0.00014 | 0.006002 |
| [101969793](https://www.ncbi.nlm.nih.gov/entrez/query.fcgi?db=gene&cmd=Retrieve&dopt=full_report&list_uids=101969793) | Rhbdf2 | 1.493374 | 2.815466 | 0.000641 | 0.018451 |
| [101962402](https://www.ncbi.nlm.nih.gov/entrez/query.fcgi?db=gene&cmd=Retrieve&dopt=full_report&list_uids=101962402) | Abtb2 | 1.490949 | 2.810738 | 0.0015 | 0.034467 |
| [101957761](https://www.ncbi.nlm.nih.gov/entrez/query.fcgi?db=gene&cmd=Retrieve&dopt=full_report&list_uids=101957761) | Smtnl2 | 1.483306 | 2.795887 | 2.08E-08 | 3.23E-06 |
| [101962107](https://www.ncbi.nlm.nih.gov/entrez/query.fcgi?db=gene&cmd=Retrieve&dopt=full_report&list_uids=101962107) | Cat | 1.476956 | 2.783608 | 1.99E-06 | 0.000173 |
| [101967779](https://www.ncbi.nlm.nih.gov/entrez/query.fcgi?db=gene&cmd=Retrieve&dopt=full_report&list_uids=101967779) | Cebpa | 1.471143 | 2.772415 | 0.001948 | 0.041276 |
| [101974859](https://www.ncbi.nlm.nih.gov/entrez/query.fcgi?db=gene&cmd=Retrieve&dopt=full_report&list_uids=101974859) | Plin5 | 1.462192 | 2.755268 | 0.000213 | 0.008202 |
| [101969799](https://www.ncbi.nlm.nih.gov/entrez/query.fcgi?db=gene&cmd=Retrieve&dopt=full_report&list_uids=101969799) | Arhgap20 | 1.446971 | 2.72635 | 2.04E-05 | 0.001213 |
| [101959052](https://www.ncbi.nlm.nih.gov/entrez/query.fcgi?db=gene&cmd=Retrieve&dopt=full_report&list_uids=101959052) | Ppp1r3g | 1.436297 | 2.706253 | 0.001448 | 0.033726 |
| [101966766](https://www.ncbi.nlm.nih.gov/entrez/query.fcgi?db=gene&cmd=Retrieve&dopt=full_report&list_uids=101966766) | LOC101966766 | 1.434603 | 2.703078 | 0.002288 | 0.046142 |
| [101971819](https://www.ncbi.nlm.nih.gov/entrez/query.fcgi?db=gene&cmd=Retrieve&dopt=full_report&list_uids=101971819) | Lamb3 | 1.410199 | 2.657739 | 0.000389 | 0.012797 |
| [101971706](https://www.ncbi.nlm.nih.gov/entrez/query.fcgi?db=gene&cmd=Retrieve&dopt=full_report&list_uids=101971706) | LOC101971706 | 1.400704 | 2.640304 | 0.000181 | 0.007131 |
| [101954396](https://www.ncbi.nlm.nih.gov/entrez/query.fcgi?db=gene&cmd=Retrieve&dopt=full_report&list_uids=101954396) | Celsr2 | 1.399467 | 2.638041 | 0.000152 | 0.006416 |
| [101972042](https://www.ncbi.nlm.nih.gov/entrez/query.fcgi?db=gene&cmd=Retrieve&dopt=full_report&list_uids=101972042) | LOC101972042 | 1.39635 | 2.632348 | 2.09E-06 | 0.000181 |
| [101973055](https://www.ncbi.nlm.nih.gov/entrez/query.fcgi?db=gene&cmd=Retrieve&dopt=full_report&list_uids=101973055) | LOC101973055 | 1.38663 | 2.614672 | 1.26E-07 | 1.61E-05 |
| [101976178](https://www.ncbi.nlm.nih.gov/entrez/query.fcgi?db=gene&cmd=Retrieve&dopt=full_report&list_uids=101976178) | Fsd1l | 1.385418 | 2.612477 | 3.6E-06 | 0.0003 |
| [101958312](https://www.ncbi.nlm.nih.gov/entrez/query.fcgi?db=gene&cmd=Retrieve&dopt=full_report&list_uids=101958312) | Ntn1 | 1.38099 | 2.60447 | 0.00059 | 0.017421 |
| [101954561](https://www.ncbi.nlm.nih.gov/entrez/query.fcgi?db=gene&cmd=Retrieve&dopt=full_report&list_uids=101954561) | Pdzrn3 | 1.378129 | 2.599311 | 2.32E-08 | 3.54E-06 |
| [101971461](https://www.ncbi.nlm.nih.gov/entrez/query.fcgi?db=gene&cmd=Retrieve&dopt=full_report&list_uids=101971461) | Prrx1 | 1.376614 | 2.596583 | 7.26E-06 | 0.000517 |
| [101960577](https://www.ncbi.nlm.nih.gov/entrez/query.fcgi?db=gene&cmd=Retrieve&dopt=full_report&list_uids=101960577) | Ephx1 | 1.372152 | 2.588563 | 8.65E-05 | 0.004082 |
| [101966213](https://www.ncbi.nlm.nih.gov/entrez/query.fcgi?db=gene&cmd=Retrieve&dopt=full_report&list_uids=101966213) | Ghdc | 1.36704 | 2.579408 | 3.97E-05 | 0.002145 |
| [101963455](https://www.ncbi.nlm.nih.gov/entrez/query.fcgi?db=gene&cmd=Retrieve&dopt=full_report&list_uids=101963455) | Crtac1 | 1.36417 | 2.574282 | 0.00239 | 0.047273 |
| [101963287](https://www.ncbi.nlm.nih.gov/entrez/query.fcgi?db=gene&cmd=Retrieve&dopt=full_report&list_uids=101963287) | Pemt | 1.364032 | 2.574036 | 0.00102 | 0.025696 |
| [101959398](https://www.ncbi.nlm.nih.gov/entrez/query.fcgi?db=gene&cmd=Retrieve&dopt=full_report&list_uids=101959398) | Usp18 | 1.355666 | 2.559152 | 0.000987 | 0.025227 |
| [101973838](https://www.ncbi.nlm.nih.gov/entrez/query.fcgi?db=gene&cmd=Retrieve&dopt=full_report&list_uids=101973838) | Met | 1.353897 | 2.556016 | 5.68E-06 | 0.000424 |
| [101961909](https://www.ncbi.nlm.nih.gov/entrez/query.fcgi?db=gene&cmd=Retrieve&dopt=full_report&list_uids=101961909) | Tmem86a | 1.350481 | 2.549971 | 0.000157 | 0.006569 |
| [101966729](https://www.ncbi.nlm.nih.gov/entrez/query.fcgi?db=gene&cmd=Retrieve&dopt=full_report&list_uids=101966729) | LOC101966729 | 1.328328 | 2.511114 | 6.27E-05 | 0.00312 |
| [101957767](https://www.ncbi.nlm.nih.gov/entrez/query.fcgi?db=gene&cmd=Retrieve&dopt=full_report&list_uids=101957767) | Gpc4 | 1.311073 | 2.48126 | 0.000218 | 0.008382 |
| [101954416](https://www.ncbi.nlm.nih.gov/entrez/query.fcgi?db=gene&cmd=Retrieve&dopt=full_report&list_uids=101954416) | Tspyl2 | 1.299033 | 2.460639 | 0.002419 | 0.04768 |
| [101964808](https://www.ncbi.nlm.nih.gov/entrez/query.fcgi?db=gene&cmd=Retrieve&dopt=full_report&list_uids=101964808) | Erbb2ip | 1.29317 | 2.450659 | 6.47E-06 | 0.000474 |
| [101970501](https://www.ncbi.nlm.nih.gov/entrez/query.fcgi?db=gene&cmd=Retrieve&dopt=full_report&list_uids=101970501) | Ppm1l | 1.287001 | 2.440202 | 1.86E-06 | 0.000164 |
| [101974513](https://www.ncbi.nlm.nih.gov/entrez/query.fcgi?db=gene&cmd=Retrieve&dopt=full_report&list_uids=101974513) | Spag1 | 1.279112 | 2.426895 | 0.000908 | 0.023501 |
| [101975276](https://www.ncbi.nlm.nih.gov/entrez/query.fcgi?db=gene&cmd=Retrieve&dopt=full_report&list_uids=101975276) | LOC101975276 | 1.259065 | 2.393406 | 3.12E-06 | 0.000262 |
| [101961107](https://www.ncbi.nlm.nih.gov/entrez/query.fcgi?db=gene&cmd=Retrieve&dopt=full_report&list_uids=101961107) | Pgrmc1 | 1.255454 | 2.387423 | 0.000315 | 0.010985 |
| [101975344](https://www.ncbi.nlm.nih.gov/entrez/query.fcgi?db=gene&cmd=Retrieve&dopt=full_report&list_uids=101975344) | Plce1 | 1.253149 | 2.383612 | 0.000624 | 0.018132 |
| [101961575](https://www.ncbi.nlm.nih.gov/entrez/query.fcgi?db=gene&cmd=Retrieve&dopt=full_report&list_uids=101961575) | Eya1 | 1.241995 | 2.365254 | 2.18E-05 | 0.001284 |
| [101961354](https://www.ncbi.nlm.nih.gov/entrez/query.fcgi?db=gene&cmd=Retrieve&dopt=full_report&list_uids=101961354) | Taf9b | 1.24076 | 2.36323 | 3.8E-05 | 0.002081 |
| [101975330](https://www.ncbi.nlm.nih.gov/entrez/query.fcgi?db=gene&cmd=Retrieve&dopt=full_report&list_uids=101975330) | Tmem38b | 1.238579 | 2.359659 | 0.000245 | 0.009157 |
| [101958402](https://www.ncbi.nlm.nih.gov/entrez/query.fcgi?db=gene&cmd=Retrieve&dopt=full_report&list_uids=101958402) | Tmem120b | 1.233858 | 2.351951 | 6.26E-07 | 6.41E-05 |
| [101975357](https://www.ncbi.nlm.nih.gov/entrez/query.fcgi?db=gene&cmd=Retrieve&dopt=full_report&list_uids=101975357) | LOC101975357 | 1.23241 | 2.349591 | 0.001803 | 0.039125 |
| [101975205](https://www.ncbi.nlm.nih.gov/entrez/query.fcgi?db=gene&cmd=Retrieve&dopt=full_report&list_uids=101975205) | Ucp2 | 1.221919 | 2.332568 | 0.001689 | 0.037427 |
| [101970747](https://www.ncbi.nlm.nih.gov/entrez/query.fcgi?db=gene&cmd=Retrieve&dopt=full_report&list_uids=101970747) | Cxxc5 | 1.207454 | 2.309298 | 2.39E-05 | 0.001395 |
| [101957454](https://www.ncbi.nlm.nih.gov/entrez/query.fcgi?db=gene&cmd=Retrieve&dopt=full_report&list_uids=101957454) | Fancd2 | 1.20182 | 2.300296 | 0.00149 | 0.034364 |
| [101964202](https://www.ncbi.nlm.nih.gov/entrez/query.fcgi?db=gene&cmd=Retrieve&dopt=full_report&list_uids=101964202) | Gramd3 | 1.198045 | 2.294285 | 0.000235 | 0.008877 |
| [101958168](https://www.ncbi.nlm.nih.gov/entrez/query.fcgi?db=gene&cmd=Retrieve&dopt=full_report&list_uids=101958168) | Slc25a17 | 1.197154 | 2.292869 | 0.000191 | 0.007512 |
| [101963429](https://www.ncbi.nlm.nih.gov/entrez/query.fcgi?db=gene&cmd=Retrieve&dopt=full_report&list_uids=101963429) | Prkag3 | 1.185797 | 2.27489 | 4.7E-05 | 0.002447 |
| [101969885](https://www.ncbi.nlm.nih.gov/entrez/query.fcgi?db=gene&cmd=Retrieve&dopt=full_report&list_uids=101969885) | Aacs | 1.182659 | 2.269948 | 3.37E-05 | 0.001887 |
| [101957018](https://www.ncbi.nlm.nih.gov/entrez/query.fcgi?db=gene&cmd=Retrieve&dopt=full_report&list_uids=101957018) | LOC101957018 | 1.180617 | 2.266737 | 0.002256 | 0.045689 |
| [101964642](https://www.ncbi.nlm.nih.gov/entrez/query.fcgi?db=gene&cmd=Retrieve&dopt=full_report&list_uids=101964642) | Srsf5 | 1.169057 | 2.248646 | 0.000238 | 0.008947 |
| [101957345](https://www.ncbi.nlm.nih.gov/entrez/query.fcgi?db=gene&cmd=Retrieve&dopt=full_report&list_uids=101957345) | Scn4b | 1.166005 | 2.243895 | 0.001002 | 0.025432 |
| [101977569](https://www.ncbi.nlm.nih.gov/entrez/query.fcgi?db=gene&cmd=Retrieve&dopt=full_report&list_uids=101977569) | Kctd15 | 1.160103 | 2.234733 | 3.96E-05 | 0.002145 |
| [101975284](https://www.ncbi.nlm.nih.gov/entrez/query.fcgi?db=gene&cmd=Retrieve&dopt=full_report&list_uids=101975284) | Agbl1 | 1.155403 | 2.227465 | 0.00022 | 0.008416 |
| [101955019](https://www.ncbi.nlm.nih.gov/entrez/query.fcgi?db=gene&cmd=Retrieve&dopt=full_report&list_uids=101955019) | Slc25a42 | 1.150526 | 2.219949 | 4.86E-05 | 0.002511 |
| [101977465](https://www.ncbi.nlm.nih.gov/entrez/query.fcgi?db=gene&cmd=Retrieve&dopt=full_report&list_uids=101977465) | Hdac9 | 1.148202 | 2.216375 | 0.002216 | 0.045086 |
| [101974392](https://www.ncbi.nlm.nih.gov/entrez/query.fcgi?db=gene&cmd=Retrieve&dopt=full_report&list_uids=101974392) | Pfkfb2 | 1.144075 | 2.210044 | 0.002541 | 0.04889 |
| [101966572](https://www.ncbi.nlm.nih.gov/entrez/query.fcgi?db=gene&cmd=Retrieve&dopt=full_report&list_uids=101966572) | Rnf122 | 1.135086 | 2.196317 | 0.000329 | 0.011396 |
| [101954306](https://www.ncbi.nlm.nih.gov/entrez/query.fcgi?db=gene&cmd=Retrieve&dopt=full_report&list_uids=101954306) | Plk2 | 1.111444 | 2.160617 | 0.001512 | 0.034679 |
| [101976297](https://www.ncbi.nlm.nih.gov/entrez/query.fcgi?db=gene&cmd=Retrieve&dopt=full_report&list_uids=101976297) | Myf6 | 1.111097 | 2.160098 | 0.00044 | 0.014005 |
| [101958070](https://www.ncbi.nlm.nih.gov/entrez/query.fcgi?db=gene&cmd=Retrieve&dopt=full_report&list_uids=101958070) | Rnf216 | 1.110593 | 2.159345 | 0.001835 | 0.039615 |
| [101968617](https://www.ncbi.nlm.nih.gov/entrez/query.fcgi?db=gene&cmd=Retrieve&dopt=full_report&list_uids=101968617) | Afap1l1 | 1.106064 | 2.152575 | 0.000476 | 0.014867 |
| [101962197](https://www.ncbi.nlm.nih.gov/entrez/query.fcgi?db=gene&cmd=Retrieve&dopt=full_report&list_uids=101962197) | Oaf | 1.104029 | 2.149541 | 0.001627 | 0.036275 |
| [101962030](https://www.ncbi.nlm.nih.gov/entrez/query.fcgi?db=gene&cmd=Retrieve&dopt=full_report&list_uids=101962030) | Txnrd2 | 1.10206 | 2.146609 | 3.64E-05 | 0.002 |
| [101962349](https://www.ncbi.nlm.nih.gov/entrez/query.fcgi?db=gene&cmd=Retrieve&dopt=full_report&list_uids=101962349) | Ahdc1 | 1.100086 | 2.143674 | 0.000408 | 0.01325 |
| [101956982](https://www.ncbi.nlm.nih.gov/entrez/query.fcgi?db=gene&cmd=Retrieve&dopt=full_report&list_uids=101956982) | Ldlr | 1.099944 | 2.143464 | 0.000661 | 0.018898 |
| [106144883](https://www.ncbi.nlm.nih.gov/entrez/query.fcgi?db=gene&cmd=Retrieve&dopt=full_report&list_uids=106144883) | LOC106144883 | 1.097962 | 2.140521 | 0.000273 | 0.009959 |
| [101956444](https://www.ncbi.nlm.nih.gov/entrez/query.fcgi?db=gene&cmd=Retrieve&dopt=full_report&list_uids=101956444) | Hlcs | 1.093435 | 2.133814 | 0.001148 | 0.028375 |
| [101976442](https://www.ncbi.nlm.nih.gov/entrez/query.fcgi?db=gene&cmd=Retrieve&dopt=full_report&list_uids=101976442) | Frzb | 1.092377 | 2.13225 | 0.000482 | 0.015005 |
| [101962486](https://www.ncbi.nlm.nih.gov/entrez/query.fcgi?db=gene&cmd=Retrieve&dopt=full_report&list_uids=101962486) | Atp1b3 | 1.090198 | 2.129033 | 0.000328 | 0.011396 |
| [101965785](https://www.ncbi.nlm.nih.gov/entrez/query.fcgi?db=gene&cmd=Retrieve&dopt=full_report&list_uids=101965785) | Rbm3 | 1.087693 | 2.125339 | 0.000204 | 0.007892 |
| [101959761](https://www.ncbi.nlm.nih.gov/entrez/query.fcgi?db=gene&cmd=Retrieve&dopt=full_report&list_uids=101959761) | Stk26 | 1.081853 | 2.116754 | 0.000825 | 0.021863 |
| [101957829](https://www.ncbi.nlm.nih.gov/entrez/query.fcgi?db=gene&cmd=Retrieve&dopt=full_report&list_uids=101957829) | Ctnnal1 | 1.078477 | 2.111805 | 0.000135 | 0.005824 |
| [101974695](https://www.ncbi.nlm.nih.gov/entrez/query.fcgi?db=gene&cmd=Retrieve&dopt=full_report&list_uids=101974695) | Clu | 1.076945 | 2.109565 | 0.001984 | 0.041632 |
| [101976716](https://www.ncbi.nlm.nih.gov/entrez/query.fcgi?db=gene&cmd=Retrieve&dopt=full_report&list_uids=101976716) | Rasgrp3 | 1.071036 | 2.100942 | 0.000619 | 0.018075 |
| [101959472](https://www.ncbi.nlm.nih.gov/entrez/query.fcgi?db=gene&cmd=Retrieve&dopt=full_report&list_uids=101959472) | Rap2c | 1.062552 | 2.088623 | 0.000364 | 0.012219 |
| [101959046](https://www.ncbi.nlm.nih.gov/entrez/query.fcgi?db=gene&cmd=Retrieve&dopt=full_report&list_uids=101959046) | Slc38a4 | 1.05767 | 2.081567 | 0.00041 | 0.01325 |
| [101976477](https://www.ncbi.nlm.nih.gov/entrez/query.fcgi?db=gene&cmd=Retrieve&dopt=full_report&list_uids=101976477) | Xrcc3 | 1.056054 | 2.079237 | 0.001374 | 0.032528 |
| [101978492](https://www.ncbi.nlm.nih.gov/entrez/query.fcgi?db=gene&cmd=Retrieve&dopt=full_report&list_uids=101978492) | B4galt5 | 1.055302 | 2.078154 | 0.000611 | 0.017907 |
| [101959257](https://www.ncbi.nlm.nih.gov/entrez/query.fcgi?db=gene&cmd=Retrieve&dopt=full_report&list_uids=101959257) | Mansc1 | 1.049313 | 2.069544 | 0.00254 | 0.04889 |
| [101959430](https://www.ncbi.nlm.nih.gov/entrez/query.fcgi?db=gene&cmd=Retrieve&dopt=full_report&list_uids=101959430) | Reep5 | 1.039154 | 2.055022 | 0.000167 | 0.006902 |
| [101963168](https://www.ncbi.nlm.nih.gov/entrez/query.fcgi?db=gene&cmd=Retrieve&dopt=full_report&list_uids=101963168) | Impa2 | 1.038437 | 2.054002 | 0.000708 | 0.019895 |
| [101971740](https://www.ncbi.nlm.nih.gov/entrez/query.fcgi?db=gene&cmd=Retrieve&dopt=full_report&list_uids=101971740) | Dap | 1.035153 | 2.049332 | 0.000282 | 0.010208 |
| [101958941](https://www.ncbi.nlm.nih.gov/entrez/query.fcgi?db=gene&cmd=Retrieve&dopt=full_report&list_uids=101958941) | Acsl4 | 1.022072 | 2.030834 | 0.000168 | 0.00691 |
| [101975796](https://www.ncbi.nlm.nih.gov/entrez/query.fcgi?db=gene&cmd=Retrieve&dopt=full_report&list_uids=101975796) | Slc43a3 | 1.019792 | 2.027627 | 4.68E-05 | 0.002447 |
| [101958905](https://www.ncbi.nlm.nih.gov/entrez/query.fcgi?db=gene&cmd=Retrieve&dopt=full_report&list_uids=101958905) | LOC101958905 | 1.017588 | 2.024531 | 0.001212 | 0.029889 |
| [101955620](https://www.ncbi.nlm.nih.gov/entrez/query.fcgi?db=gene&cmd=Retrieve&dopt=full_report&list_uids=101955620) | Trpm4 | 1.014768 | 2.020578 | 0.000331 | 0.011444 |
| [101968306](https://www.ncbi.nlm.nih.gov/entrez/query.fcgi?db=gene&cmd=Retrieve&dopt=full_report&list_uids=101968306) | Dkc1 | 1.014624 | 2.020376 | 0.000356 | 0.012018 |
| [101966165](https://www.ncbi.nlm.nih.gov/entrez/query.fcgi?db=gene&cmd=Retrieve&dopt=full_report&list_uids=101966165) | Piezo1 | 1.014188 | 2.019766 | 0.000955 | 0.024512 |
| [101955988](https://www.ncbi.nlm.nih.gov/entrez/query.fcgi?db=gene&cmd=Retrieve&dopt=full_report&list_uids=101955988) | Hebp1 | 1.012483 | 2.01738 | 0.001422 | 0.033233 |
| [101970856](https://www.ncbi.nlm.nih.gov/entrez/query.fcgi?db=gene&cmd=Retrieve&dopt=full_report&list_uids=101970856) | Exosc5 | 1.012209 | 2.016997 | 0.000897 | 0.023369 |
| [101961588](https://www.ncbi.nlm.nih.gov/entrez/query.fcgi?db=gene&cmd=Retrieve&dopt=full_report&list_uids=101961588) | LOC101961588 | 1.010709 | 2.014901 | 0.002226 | 0.045218 |
| [101956951](https://www.ncbi.nlm.nih.gov/entrez/query.fcgi?db=gene&cmd=Retrieve&dopt=full_report&list_uids=101956951) | Dhcr24 | 1.010673 | 2.014851 | 0.000814 | 0.021751 |
| [101957133](https://www.ncbi.nlm.nih.gov/entrez/query.fcgi?db=gene&cmd=Retrieve&dopt=full_report&list_uids=101957133) | Dusp12 | 1.008005 | 2.011128 | 0.000295 | 0.010468 |
| [101962758](https://www.ncbi.nlm.nih.gov/entrez/query.fcgi?db=gene&cmd=Retrieve&dopt=full_report&list_uids=101962758) | St8sia5 | 1.002949 | 2.004093 | 0.001555 | 0.035379 |
| [101966501](https://www.ncbi.nlm.nih.gov/entrez/query.fcgi?db=gene&cmd=Retrieve&dopt=full_report&list_uids=101966501) | Igf2 | 1.000331 | 2.000459 | 0.000586 | 0.01735 |
| [101966639](https://www.ncbi.nlm.nih.gov/entrez/query.fcgi?db=gene&cmd=Retrieve&dopt=full_report&list_uids=101966639) | Exoc6 | 1.000311 | 2.000431 | 8.8E-05 | 0.00414 |
| [101971984](https://www.ncbi.nlm.nih.gov/entrez/query.fcgi?db=gene&cmd=Retrieve&dopt=full_report&list_uids=101971984) | Sel1l | 0.998637 | 1.998111 | 0.000177 | 0.007118 |
| [101969040](https://www.ncbi.nlm.nih.gov/entrez/query.fcgi?db=gene&cmd=Retrieve&dopt=full_report&list_uids=101969040) | Pitpnm1 | 0.997625 | 1.99671 | 0.000806 | 0.021702 |
| [101959446](https://www.ncbi.nlm.nih.gov/entrez/query.fcgi?db=gene&cmd=Retrieve&dopt=full_report&list_uids=101959446) | Cst6 | 0.99586 | 1.99427 | 0.002046 | 0.0426 |
| [101967389](https://www.ncbi.nlm.nih.gov/entrez/query.fcgi?db=gene&cmd=Retrieve&dopt=full_report&list_uids=101967389) | Tdrd7 | 0.984174 | 1.978181 | 0.000641 | 0.018451 |
| [101961829](https://www.ncbi.nlm.nih.gov/entrez/query.fcgi?db=gene&cmd=Retrieve&dopt=full_report&list_uids=101961829) | Psmg4 | 0.979503 | 1.971786 | 0.00099 | 0.02524 |
| [101958758](https://www.ncbi.nlm.nih.gov/entrez/query.fcgi?db=gene&cmd=Retrieve&dopt=full_report&list_uids=101958758) | Mrps6 | 0.978241 | 1.970062 | 0.000582 | 0.01731 |
| [101957513](https://www.ncbi.nlm.nih.gov/entrez/query.fcgi?db=gene&cmd=Retrieve&dopt=full_report&list_uids=101957513) | Asph | 0.959352 | 1.944437 | 0.001385 | 0.032634 |
| [101954605](https://www.ncbi.nlm.nih.gov/entrez/query.fcgi?db=gene&cmd=Retrieve&dopt=full_report&list_uids=101954605) | LOC101954605 | 0.954911 | 1.938461 | 0.000783 | 0.021278 |
| [101961125](https://www.ncbi.nlm.nih.gov/entrez/query.fcgi?db=gene&cmd=Retrieve&dopt=full_report&list_uids=101961125) | Klhl33 | 0.95182 | 1.934311 | 0.001361 | 0.032324 |
| [101972733](https://www.ncbi.nlm.nih.gov/entrez/query.fcgi?db=gene&cmd=Retrieve&dopt=full_report&list_uids=101972733) | Smtn | 0.945874 | 1.926355 | 0.000469 | 0.014748 |
| [101973381](https://www.ncbi.nlm.nih.gov/entrez/query.fcgi?db=gene&cmd=Retrieve&dopt=full_report&list_uids=101973381) | LOC101973381 | 0.943383 | 1.923033 | 0.000939 | 0.024143 |
| [101963323](https://www.ncbi.nlm.nih.gov/entrez/query.fcgi?db=gene&cmd=Retrieve&dopt=full_report&list_uids=101963323) | Fmr1 | 0.935729 | 1.912857 | 0.001292 | 0.031368 |
| [101973291](https://www.ncbi.nlm.nih.gov/entrez/query.fcgi?db=gene&cmd=Retrieve&dopt=full_report&list_uids=101973291) | Alg5 | 0.934151 | 1.910766 | 0.001342 | 0.032118 |
| [101955095](https://www.ncbi.nlm.nih.gov/entrez/query.fcgi?db=gene&cmd=Retrieve&dopt=full_report&list_uids=101955095) | Dyrk2 | 0.933171 | 1.909468 | 0.002326 | 0.046584 |
| [101974916](https://www.ncbi.nlm.nih.gov/entrez/query.fcgi?db=gene&cmd=Retrieve&dopt=full_report&list_uids=101974916) | Chpt1 | 0.924548 | 1.89809 | 0.000496 | 0.015221 |
| [101975603](https://www.ncbi.nlm.nih.gov/entrez/query.fcgi?db=gene&cmd=Retrieve&dopt=full_report&list_uids=101975603) | Sc5d | 0.924372 | 1.897858 | 0.000314 | 0.010985 |
| [101954397](https://www.ncbi.nlm.nih.gov/entrez/query.fcgi?db=gene&cmd=Retrieve&dopt=full_report&list_uids=101954397) | Fam213a | 0.914135 | 1.884439 | 0.000621 | 0.01808 |
| [101976363](https://www.ncbi.nlm.nih.gov/entrez/query.fcgi?db=gene&cmd=Retrieve&dopt=full_report&list_uids=101976363) | Chrnb1 | 0.903954 | 1.871187 | 0.000391 | 0.012797 |
| [101966254](https://www.ncbi.nlm.nih.gov/entrez/query.fcgi?db=gene&cmd=Retrieve&dopt=full_report&list_uids=101966254) | Morn4 | 0.896012 | 1.860914 | 0.002444 | 0.047722 |
| [101961881](https://www.ncbi.nlm.nih.gov/entrez/query.fcgi?db=gene&cmd=Retrieve&dopt=full_report&list_uids=101961881) | Ppia | 0.883241 | 1.844515 | 0.002464 | 0.047722 |
| [101969613](https://www.ncbi.nlm.nih.gov/entrez/query.fcgi?db=gene&cmd=Retrieve&dopt=full_report&list_uids=101969613) | Acot9 | 0.882045 | 1.842986 | 0.00087 | 0.02288 |
| [101974603](https://www.ncbi.nlm.nih.gov/entrez/query.fcgi?db=gene&cmd=Retrieve&dopt=full_report&list_uids=101974603) | Ngly1 | 0.878497 | 1.838459 | 0.000294 | 0.010468 |
| [101973826](https://www.ncbi.nlm.nih.gov/entrez/query.fcgi?db=gene&cmd=Retrieve&dopt=full_report&list_uids=101973826) | Pole4 | 0.875596 | 1.834766 | 0.001044 | 0.026182 |
| [101968966](https://www.ncbi.nlm.nih.gov/entrez/query.fcgi?db=gene&cmd=Retrieve&dopt=full_report&list_uids=101968966) | Gfer | 0.871774 | 1.829912 | 0.000407 | 0.01325 |
| [101978136](https://www.ncbi.nlm.nih.gov/entrez/query.fcgi?db=gene&cmd=Retrieve&dopt=full_report&list_uids=101978136) | Tpd52 | 0.871134 | 1.8291 | 0.00093 | 0.023975 |
| [101977126](https://www.ncbi.nlm.nih.gov/entrez/query.fcgi?db=gene&cmd=Retrieve&dopt=full_report&list_uids=101977126) | Acsl6 | 0.858304 | 1.812906 | 0.001864 | 0.040144 |
| [101954479](https://www.ncbi.nlm.nih.gov/entrez/query.fcgi?db=gene&cmd=Retrieve&dopt=full_report&list_uids=101954479) | Efr3a | 0.852626 | 1.805785 | 0.000792 | 0.021456 |
| [101960962](https://www.ncbi.nlm.nih.gov/entrez/query.fcgi?db=gene&cmd=Retrieve&dopt=full_report&list_uids=101960962) | Rnf38 | 0.851662 | 1.804578 | 0.002347 | 0.046845 |
| [101964183](https://www.ncbi.nlm.nih.gov/entrez/query.fcgi?db=gene&cmd=Retrieve&dopt=full_report&list_uids=101964183) | Cln5 | 0.850927 | 1.80366 | 0.001107 | 0.027585 |
| [101978454](https://www.ncbi.nlm.nih.gov/entrez/query.fcgi?db=gene&cmd=Retrieve&dopt=full_report&list_uids=101978454) | Creg1 | 0.847624 | 1.799535 | 0.002073 | 0.042906 |
| [101978550](https://www.ncbi.nlm.nih.gov/entrez/query.fcgi?db=gene&cmd=Retrieve&dopt=full_report&list_uids=101978550) | Cpeb2 | 0.846713 | 1.798399 | 0.001404 | 0.03295 |
| [101959010](https://www.ncbi.nlm.nih.gov/entrez/query.fcgi?db=gene&cmd=Retrieve&dopt=full_report&list_uids=101959010) | Polr1b | 0.822615 | 1.768609 | 0.002203 | 0.044973 |
| [101957486](https://www.ncbi.nlm.nih.gov/entrez/query.fcgi?db=gene&cmd=Retrieve&dopt=full_report&list_uids=101957486) | Fbxo9 | 0.809931 | 1.753127 | 0.001239 | 0.030249 |
| [101976559](https://www.ncbi.nlm.nih.gov/entrez/query.fcgi?db=gene&cmd=Retrieve&dopt=full_report&list_uids=101976559) | Popdc3 | 0.80031 | 1.741476 | 0.001542 | 0.035191 |
| [101968145](https://www.ncbi.nlm.nih.gov/entrez/query.fcgi?db=gene&cmd=Retrieve&dopt=full_report&list_uids=101968145) | Larp7 | 0.792122 | 1.73162 | 0.002523 | 0.04871 |
| [101957560](https://www.ncbi.nlm.nih.gov/entrez/query.fcgi?db=gene&cmd=Retrieve&dopt=full_report&list_uids=101957560) | Pxmp2 | 0.792046 | 1.731529 | 0.002126 | 0.043769 |
| [101965737](https://www.ncbi.nlm.nih.gov/entrez/query.fcgi?db=gene&cmd=Retrieve&dopt=full_report&list_uids=101965737) | Naa50 | 0.79141 | 1.730766 | 0.001323 | 0.03185 |
| [101957552](https://www.ncbi.nlm.nih.gov/entrez/query.fcgi?db=gene&cmd=Retrieve&dopt=full_report&list_uids=101957552) | Kiaa1191 | 0.788538 | 1.727324 | 0.002445 | 0.047722 |
| [101977470](https://www.ncbi.nlm.nih.gov/entrez/query.fcgi?db=gene&cmd=Retrieve&dopt=full_report&list_uids=101977470) | Tmem9 | 0.786469 | 1.724848 | 0.002015 | 0.042194 |
| [101977153](https://www.ncbi.nlm.nih.gov/entrez/query.fcgi?db=gene&cmd=Retrieve&dopt=full_report&list_uids=101977153) | Spint2 | 0.77307 | 1.708903 | 0.001419 | 0.033224 |
| [101965678](https://www.ncbi.nlm.nih.gov/entrez/query.fcgi?db=gene&cmd=Retrieve&dopt=full_report&list_uids=101965678) | Parvb | -0.7624 | -1.69631 | 0.00135 | 0.032122 |
| [101975385](https://www.ncbi.nlm.nih.gov/entrez/query.fcgi?db=gene&cmd=Retrieve&dopt=full_report&list_uids=101975385) | Tob1 | -0.78458 | -1.72259 | 0.001901 | 0.040596 |
| [101958027](https://www.ncbi.nlm.nih.gov/entrez/query.fcgi?db=gene&cmd=Retrieve&dopt=full_report&list_uids=101958027) | Fuca2 | -0.81596 | -1.76047 | 0.002071 | 0.042906 |
| [101969460](https://www.ncbi.nlm.nih.gov/entrez/query.fcgi?db=gene&cmd=Retrieve&dopt=full_report&list_uids=101969460) | LOC101969460 | -0.8188 | -1.76394 | 0.002126 | 0.043769 |
| [101956019](https://www.ncbi.nlm.nih.gov/entrez/query.fcgi?db=gene&cmd=Retrieve&dopt=full_report&list_uids=101956019) | Col4a1 | -0.82117 | -1.76683 | 0.001836 | 0.039615 |
| [101977625](https://www.ncbi.nlm.nih.gov/entrez/query.fcgi?db=gene&cmd=Retrieve&dopt=full_report&list_uids=101977625) | Ppp1r12c | -0.82183 | -1.76764 | 0.002144 | 0.043984 |
| [101976157](https://www.ncbi.nlm.nih.gov/entrez/query.fcgi?db=gene&cmd=Retrieve&dopt=full_report&list_uids=101976157) | Epas1 | -0.82334 | -1.7695 | 0.002257 | 0.045689 |
| [101968489](https://www.ncbi.nlm.nih.gov/entrez/query.fcgi?db=gene&cmd=Retrieve&dopt=full_report&list_uids=101968489) | Tagln2 | -0.83274 | -1.78107 | 0.001142 | 0.028345 |
| [101978700](https://www.ncbi.nlm.nih.gov/entrez/query.fcgi?db=gene&cmd=Retrieve&dopt=full_report&list_uids=101978700) | Atf7ip | -0.83756 | -1.78703 | 0.001965 | 0.041516 |
| [101964061](https://www.ncbi.nlm.nih.gov/entrez/query.fcgi?db=gene&cmd=Retrieve&dopt=full_report&list_uids=101964061) | Pde12 | -0.83757 | -1.78703 | 0.001939 | 0.041255 |
| [101967336](https://www.ncbi.nlm.nih.gov/entrez/query.fcgi?db=gene&cmd=Retrieve&dopt=full_report&list_uids=101967336) | Cd99l2 | -0.85258 | -1.80573 | 0.001321 | 0.03185 |
| [101970546](https://www.ncbi.nlm.nih.gov/entrez/query.fcgi?db=gene&cmd=Retrieve&dopt=full_report&list_uids=101970546) | Mapre2 | -0.87464 | -1.83356 | 0.001808 | 0.039151 |
| [101961672](https://www.ncbi.nlm.nih.gov/entrez/query.fcgi?db=gene&cmd=Retrieve&dopt=full_report&list_uids=101961672) | Klhdc3 | -0.87595 | -1.83522 | 0.001482 | 0.034241 |
| [101978110](https://www.ncbi.nlm.nih.gov/entrez/query.fcgi?db=gene&cmd=Retrieve&dopt=full_report&list_uids=101978110) | Srsf6 | -0.87825 | -1.83814 | 0.001463 | 0.034002 |
| [101968921](https://www.ncbi.nlm.nih.gov/entrez/query.fcgi?db=gene&cmd=Retrieve&dopt=full_report&list_uids=101968921) | Ace | -0.9023 | -1.86904 | 0.001569 | 0.035514 |
| [101975060](https://www.ncbi.nlm.nih.gov/entrez/query.fcgi?db=gene&cmd=Retrieve&dopt=full_report&list_uids=101975060) | Cpeb4 | -0.91128 | -1.88072 | 0.001673 | 0.037238 |
| [101959116](https://www.ncbi.nlm.nih.gov/entrez/query.fcgi?db=gene&cmd=Retrieve&dopt=full_report&list_uids=101959116) | Kat6a | -0.91323 | -1.88326 | 0.001021 | 0.025696 |
| [101973060](https://www.ncbi.nlm.nih.gov/entrez/query.fcgi?db=gene&cmd=Retrieve&dopt=full_report&list_uids=101973060) | Rbm5 | -0.91493 | -1.88548 | 0.000677 | 0.019238 |
| [101977233](https://www.ncbi.nlm.nih.gov/entrez/query.fcgi?db=gene&cmd=Retrieve&dopt=full_report&list_uids=101977233) | Vegfa | -0.91647 | -1.88749 | 0.002121 | 0.043769 |
| [101959200](https://www.ncbi.nlm.nih.gov/entrez/query.fcgi?db=gene&cmd=Retrieve&dopt=full_report&list_uids=101959200) | Brd2 | -0.91939 | -1.89131 | 0.000372 | 0.012415 |
| [101965681](https://www.ncbi.nlm.nih.gov/entrez/query.fcgi?db=gene&cmd=Retrieve&dopt=full_report&list_uids=101965681) | Pla2g15 | -0.92173 | -1.89438 | 0.001975 | 0.041579 |
| [101961037](https://www.ncbi.nlm.nih.gov/entrez/query.fcgi?db=gene&cmd=Retrieve&dopt=full_report&list_uids=101961037) | Phrf1 | -0.92367 | -1.89693 | 0.001692 | 0.037427 |
| [101960436](https://www.ncbi.nlm.nih.gov/entrez/query.fcgi?db=gene&cmd=Retrieve&dopt=full_report&list_uids=101960436) | Adcy4 | -0.92593 | -1.89991 | 0.001041 | 0.026165 |
| [101978324](https://www.ncbi.nlm.nih.gov/entrez/query.fcgi?db=gene&cmd=Retrieve&dopt=full_report&list_uids=101978324) | Fnip1 | -0.92618 | -1.90024 | 0.000581 | 0.01731 |
| [101970212](https://www.ncbi.nlm.nih.gov/entrez/query.fcgi?db=gene&cmd=Retrieve&dopt=full_report&list_uids=101970212) | Klhl7 | -0.92867 | -1.90352 | 0.000286 | 0.010256 |
| [101977819](https://www.ncbi.nlm.nih.gov/entrez/query.fcgi?db=gene&cmd=Retrieve&dopt=full_report&list_uids=101977819) | Znf532 | -0.93236 | -1.9084 | 0.000876 | 0.022957 |
| [101978138](https://www.ncbi.nlm.nih.gov/entrez/query.fcgi?db=gene&cmd=Retrieve&dopt=full_report&list_uids=101978138) | Calm3 | -0.9372 | -1.9148 | 0.000958 | 0.024536 |
| [101965126](https://www.ncbi.nlm.nih.gov/entrez/query.fcgi?db=gene&cmd=Retrieve&dopt=full_report&list_uids=101965126) | Sun2 | -0.93786 | -1.91568 | 0.001613 | 0.036208 |
| [101971500](https://www.ncbi.nlm.nih.gov/entrez/query.fcgi?db=gene&cmd=Retrieve&dopt=full_report&list_uids=101971500) | Znf746 | -0.94078 | -1.91956 | 0.0018 | 0.039113 |
| [101976503](https://www.ncbi.nlm.nih.gov/entrez/query.fcgi?db=gene&cmd=Retrieve&dopt=full_report&list_uids=101976503) | Prom1 | -0.94317 | -1.92274 | 0.002048 | 0.0426 |
| [101962055](https://www.ncbi.nlm.nih.gov/entrez/query.fcgi?db=gene&cmd=Retrieve&dopt=full_report&list_uids=101962055) | Wasf2 | -0.94383 | -1.92362 | 0.000675 | 0.019233 |
| [101956498](https://www.ncbi.nlm.nih.gov/entrez/query.fcgi?db=gene&cmd=Retrieve&dopt=full_report&list_uids=101956498) | Adal | -0.94401 | -1.92386 | 0.001266 | 0.030856 |
| [101961783](https://www.ncbi.nlm.nih.gov/entrez/query.fcgi?db=gene&cmd=Retrieve&dopt=full_report&list_uids=101961783) | Rc3h1 | -0.97107 | -1.96029 | 0.001871 | 0.040229 |
| [101977987](https://www.ncbi.nlm.nih.gov/entrez/query.fcgi?db=gene&cmd=Retrieve&dopt=full_report&list_uids=101977987) | Aldh6a1 | -0.98069 | -1.9734 | 0.00034 | 0.011631 |
| [101970298](https://www.ncbi.nlm.nih.gov/entrez/query.fcgi?db=gene&cmd=Retrieve&dopt=full_report&list_uids=101970298) | Adgrf5 | -0.98342 | -1.97715 | 0.002461 | 0.047722 |
| [101963735](https://www.ncbi.nlm.nih.gov/entrez/query.fcgi?db=gene&cmd=Retrieve&dopt=full_report&list_uids=101963735) | Sec31b | -0.98358 | -1.97736 | 0.000584 | 0.017342 |
| [101976350](https://www.ncbi.nlm.nih.gov/entrez/query.fcgi?db=gene&cmd=Retrieve&dopt=full_report&list_uids=101976350) | Mlxip | -0.98638 | -1.9812 | 0.00047 | 0.014748 |
| [101977147](https://www.ncbi.nlm.nih.gov/entrez/query.fcgi?db=gene&cmd=Retrieve&dopt=full_report&list_uids=101977147) | Pde4d | -0.98739 | -1.98259 | 0.001756 | 0.038437 |
| [101964351](https://www.ncbi.nlm.nih.gov/entrez/query.fcgi?db=gene&cmd=Retrieve&dopt=full_report&list_uids=101964351) | Ramp3 | -0.98816 | -1.98365 | 0.002387 | 0.047273 |
| [101971550](https://www.ncbi.nlm.nih.gov/entrez/query.fcgi?db=gene&cmd=Retrieve&dopt=full_report&list_uids=101971550) | Plcd1 | -0.99331 | -1.99074 | 0.001477 | 0.034184 |
| [101955300](https://www.ncbi.nlm.nih.gov/entrez/query.fcgi?db=gene&cmd=Retrieve&dopt=full_report&list_uids=101955300) | Osgin2 | -1.00113 | -2.00157 | 0.001694 | 0.037427 |
| [101964987](https://www.ncbi.nlm.nih.gov/entrez/query.fcgi?db=gene&cmd=Retrieve&dopt=full_report&list_uids=101964987) | Ammecr1l | -1.00132 | -2.00183 | 0.000547 | 0.016491 |
| [101963067](https://www.ncbi.nlm.nih.gov/entrez/query.fcgi?db=gene&cmd=Retrieve&dopt=full_report&list_uids=101963067) | Nol9 | -1.00744 | -2.01034 | 0.000816 | 0.021761 |
| [101976667](https://www.ncbi.nlm.nih.gov/entrez/query.fcgi?db=gene&cmd=Retrieve&dopt=full_report&list_uids=101976667) | Dhdh | -1.00887 | -2.01233 | 0.00018 | 0.007131 |
| [101976598](https://www.ncbi.nlm.nih.gov/entrez/query.fcgi?db=gene&cmd=Retrieve&dopt=full_report&list_uids=101976598) | Sned1 | -1.01012 | -2.01408 | 0.001884 | 0.040434 |
| [101962343](https://www.ncbi.nlm.nih.gov/entrez/query.fcgi?db=gene&cmd=Retrieve&dopt=full_report&list_uids=101962343) | Mcf2l | -1.01031 | -2.01434 | 0.000995 | 0.025324 |
| [101956221](https://www.ncbi.nlm.nih.gov/entrez/query.fcgi?db=gene&cmd=Retrieve&dopt=full_report&list_uids=101956221) | Hp1bp3 | -1.01052 | -2.01464 | 0.001426 | 0.033261 |
| [101958125](https://www.ncbi.nlm.nih.gov/entrez/query.fcgi?db=gene&cmd=Retrieve&dopt=full_report&list_uids=101958125) | Upf1 | -1.01204 | -2.01676 | 0.000302 | 0.010629 |
| [101975067](https://www.ncbi.nlm.nih.gov/entrez/query.fcgi?db=gene&cmd=Retrieve&dopt=full_report&list_uids=101975067) | Vgll2 | -1.01207 | -2.0168 | 0.001295 | 0.031368 |
| [101954731](https://www.ncbi.nlm.nih.gov/entrez/query.fcgi?db=gene&cmd=Retrieve&dopt=full_report&list_uids=101954731) | Jun | -1.0154 | -2.02146 | 0.000178 | 0.007118 |
| [101955455](https://www.ncbi.nlm.nih.gov/entrez/query.fcgi?db=gene&cmd=Retrieve&dopt=full_report&list_uids=101955455) | Srpk2 | -1.01593 | -2.02221 | 0.001922 | 0.040962 |
| [101974952](https://www.ncbi.nlm.nih.gov/entrez/query.fcgi?db=gene&cmd=Retrieve&dopt=full_report&list_uids=101974952) | Mcl1 | -1.01739 | -2.02425 | 0.001396 | 0.032817 |
| [101960467](https://www.ncbi.nlm.nih.gov/entrez/query.fcgi?db=gene&cmd=Retrieve&dopt=full_report&list_uids=101960467) | Heatr5a | -1.01855 | -2.02588 | 0.0002 | 0.007778 |
| [101975901](https://www.ncbi.nlm.nih.gov/entrez/query.fcgi?db=gene&cmd=Retrieve&dopt=full_report&list_uids=101975901) | Usp36 | -1.02254 | -2.03149 | 0.001717 | 0.037803 |
| [101968963](https://www.ncbi.nlm.nih.gov/entrez/query.fcgi?db=gene&cmd=Retrieve&dopt=full_report&list_uids=101968963) | Rsbn1 | -1.02353 | -2.03289 | 0.001236 | 0.030249 |
| [101962360](https://www.ncbi.nlm.nih.gov/entrez/query.fcgi?db=gene&cmd=Retrieve&dopt=full_report&list_uids=101962360) | Pik3r1 | -1.02366 | -2.03307 | 0.000488 | 0.015131 |
| [101974332](https://www.ncbi.nlm.nih.gov/entrez/query.fcgi?db=gene&cmd=Retrieve&dopt=full_report&list_uids=101974332) | Sdc3 | -1.03609 | -2.05066 | 0.001467 | 0.034033 |
| [101961823](https://www.ncbi.nlm.nih.gov/entrez/query.fcgi?db=gene&cmd=Retrieve&dopt=full_report&list_uids=101961823) | Nfx1 | -1.03913 | -2.05499 | 0.002139 | 0.043959 |
| [101962642](https://www.ncbi.nlm.nih.gov/entrez/query.fcgi?db=gene&cmd=Retrieve&dopt=full_report&list_uids=101962642) | Ankrd13a | -1.04072 | -2.05725 | 0.000164 | 0.006807 |
| [101968792](https://www.ncbi.nlm.nih.gov/entrez/query.fcgi?db=gene&cmd=Retrieve&dopt=full_report&list_uids=101968792) | Kiaa0226 | -1.0417 | -2.05865 | 0.001623 | 0.036266 |
| [101967580](https://www.ncbi.nlm.nih.gov/entrez/query.fcgi?db=gene&cmd=Retrieve&dopt=full_report&list_uids=101967580) | Rcor3 | -1.04229 | -2.05949 | 0.00023 | 0.008733 |
| [101975915](https://www.ncbi.nlm.nih.gov/entrez/query.fcgi?db=gene&cmd=Retrieve&dopt=full_report&list_uids=101975915) | Bcat2 | -1.0428 | -2.06022 | 0.000373 | 0.012431 |
| [101974568](https://www.ncbi.nlm.nih.gov/entrez/query.fcgi?db=gene&cmd=Retrieve&dopt=full_report&list_uids=101974568) | Csrp2 | -1.04348 | -2.0612 | 0.002036 | 0.042498 |
| [101958806](https://www.ncbi.nlm.nih.gov/entrez/query.fcgi?db=gene&cmd=Retrieve&dopt=full_report&list_uids=101958806) | Chfr | -1.05504 | -2.07777 | 0.001336 | 0.032089 |
| [101960580](https://www.ncbi.nlm.nih.gov/entrez/query.fcgi?db=gene&cmd=Retrieve&dopt=full_report&list_uids=101960580) | Noct | -1.05702 | -2.08063 | 0.000571 | 0.0171 |
| [101957471](https://www.ncbi.nlm.nih.gov/entrez/query.fcgi?db=gene&cmd=Retrieve&dopt=full_report&list_uids=101957471) | Nedd1 | -1.0582 | -2.08234 | 0.000555 | 0.016693 |
| [101966483](https://www.ncbi.nlm.nih.gov/entrez/query.fcgi?db=gene&cmd=Retrieve&dopt=full_report&list_uids=101966483) | Ldoc1l | -1.05852 | -2.08279 | 0.000402 | 0.013115 |
| [101965467](https://www.ncbi.nlm.nih.gov/entrez/query.fcgi?db=gene&cmd=Retrieve&dopt=full_report&list_uids=101965467) | Crebrf | -1.06024 | -2.08528 | 0.001019 | 0.025696 |
| [101964070](https://www.ncbi.nlm.nih.gov/entrez/query.fcgi?db=gene&cmd=Retrieve&dopt=full_report&list_uids=101964070) | Ankrd23 | -1.06109 | -2.0865 | 0.002328 | 0.046584 |
| [101974141](https://www.ncbi.nlm.nih.gov/entrez/query.fcgi?db=gene&cmd=Retrieve&dopt=full_report&list_uids=101974141) | Tmem57 | -1.06249 | -2.08854 | 6.27E-05 | 0.00312 |
| [101977408](https://www.ncbi.nlm.nih.gov/entrez/query.fcgi?db=gene&cmd=Retrieve&dopt=full_report&list_uids=101977408) | Tmem204 | -1.06622 | -2.09394 | 0.001614 | 0.036208 |
| [101960006](https://www.ncbi.nlm.nih.gov/entrez/query.fcgi?db=gene&cmd=Retrieve&dopt=full_report&list_uids=101960006) | Enah | -1.06624 | -2.09397 | 0.001295 | 0.031368 |
| [101955500](https://www.ncbi.nlm.nih.gov/entrez/query.fcgi?db=gene&cmd=Retrieve&dopt=full_report&list_uids=101955500) | Slc41a1 | -1.0682 | -2.09682 | 0.000727 | 0.020198 |
| [101964546](https://www.ncbi.nlm.nih.gov/entrez/query.fcgi?db=gene&cmd=Retrieve&dopt=full_report&list_uids=101964546) | Klf9 | -1.06922 | -2.0983 | 0.000759 | 0.020796 |
| [101970547](https://www.ncbi.nlm.nih.gov/entrez/query.fcgi?db=gene&cmd=Retrieve&dopt=full_report&list_uids=101970547) | LOC101970547 | -1.07604 | -2.10824 | 0.000811 | 0.021742 |
| [101958749](https://www.ncbi.nlm.nih.gov/entrez/query.fcgi?db=gene&cmd=Retrieve&dopt=full_report&list_uids=101958749) | LOC101958749 | -1.07759 | -2.11051 | 0.001316 | 0.03181 |
| [101968875](https://www.ncbi.nlm.nih.gov/entrez/query.fcgi?db=gene&cmd=Retrieve&dopt=full_report&list_uids=101968875) | Phka1 | -1.08108 | -2.11562 | 9.47E-05 | 0.004387 |
| [101974771](https://www.ncbi.nlm.nih.gov/entrez/query.fcgi?db=gene&cmd=Retrieve&dopt=full_report&list_uids=101974771) | Phlda3 | -1.08108 | -2.11562 | 0.001528 | 0.034967 |
| [101958858](https://www.ncbi.nlm.nih.gov/entrez/query.fcgi?db=gene&cmd=Retrieve&dopt=full_report&list_uids=101958858) | Mettl7a | -1.08133 | -2.11599 | 0.000232 | 0.008782 |
| [101954747](https://www.ncbi.nlm.nih.gov/entrez/query.fcgi?db=gene&cmd=Retrieve&dopt=full_report&list_uids=101954747) | Smg5 | -1.08154 | -2.1163 | 4.8E-06 | 0.000377 |
| [101956868](https://www.ncbi.nlm.nih.gov/entrez/query.fcgi?db=gene&cmd=Retrieve&dopt=full_report&list_uids=101956868) | Ddx47 | -1.08318 | -2.1187 | 0.000468 | 0.014748 |
| [101956930](https://www.ncbi.nlm.nih.gov/entrez/query.fcgi?db=gene&cmd=Retrieve&dopt=full_report&list_uids=101956930) | Slc22a3 | -1.08942 | -2.12788 | 0.000526 | 0.016021 |
| [101967616](https://www.ncbi.nlm.nih.gov/entrez/query.fcgi?db=gene&cmd=Retrieve&dopt=full_report&list_uids=101967616) | Phc2 | -1.09215 | -2.13191 | 4.47E-05 | 0.00235 |
| [101971841](https://www.ncbi.nlm.nih.gov/entrez/query.fcgi?db=gene&cmd=Retrieve&dopt=full_report&list_uids=101971841) | Senp7 | -1.09533 | -2.13662 | 0.001615 | 0.036208 |
| [101972294](https://www.ncbi.nlm.nih.gov/entrez/query.fcgi?db=gene&cmd=Retrieve&dopt=full_report&list_uids=101972294) | Coq10b | -1.09879 | -2.14176 | 0.000102 | 0.004708 |
| [101962049](https://www.ncbi.nlm.nih.gov/entrez/query.fcgi?db=gene&cmd=Retrieve&dopt=full_report&list_uids=101962049) | Trpm7 | -1.09913 | -2.14225 | 0.000606 | 0.017815 |
| [101972273](https://www.ncbi.nlm.nih.gov/entrez/query.fcgi?db=gene&cmd=Retrieve&dopt=full_report&list_uids=101972273) | LOC101972273 | -1.10111 | -2.1452 | 0.001899 | 0.040596 |
| [101974419](https://www.ncbi.nlm.nih.gov/entrez/query.fcgi?db=gene&cmd=Retrieve&dopt=full_report&list_uids=101974419) | Capn3 | -1.10235 | -2.14704 | 0.00075 | 0.020591 |
| [101973146](https://www.ncbi.nlm.nih.gov/entrez/query.fcgi?db=gene&cmd=Retrieve&dopt=full_report&list_uids=101973146) | Dusp6 | -1.10309 | -2.14814 | 0.000905 | 0.023463 |
| [101969924](https://www.ncbi.nlm.nih.gov/entrez/query.fcgi?db=gene&cmd=Retrieve&dopt=full_report&list_uids=101969924) | Fam126a | -1.1052 | -2.15129 | 0.000246 | 0.009157 |
| [101959584](https://www.ncbi.nlm.nih.gov/entrez/query.fcgi?db=gene&cmd=Retrieve&dopt=full_report&list_uids=101959584) | Slc20a1 | -1.10572 | -2.15207 | 0.000493 | 0.015167 |
| [101966235](https://www.ncbi.nlm.nih.gov/entrez/query.fcgi?db=gene&cmd=Retrieve&dopt=full_report&list_uids=101966235) | Fam222b | -1.10577 | -2.15214 | 0.000895 | 0.023369 |
| [101972098](https://www.ncbi.nlm.nih.gov/entrez/query.fcgi?db=gene&cmd=Retrieve&dopt=full_report&list_uids=101972098) | Akt3 | -1.10729 | -2.1544 | 0.000899 | 0.023369 |
| [101969737](https://www.ncbi.nlm.nih.gov/entrez/query.fcgi?db=gene&cmd=Retrieve&dopt=full_report&list_uids=101969737) | Ctsv | -1.1096 | -2.15786 | 0.000262 | 0.009725 |
| [101966808](https://www.ncbi.nlm.nih.gov/entrez/query.fcgi?db=gene&cmd=Retrieve&dopt=full_report&list_uids=101966808) | Aldh1a1 | -1.11224 | -2.16182 | 8.17E-05 | 0.003873 |
| [101972038](https://www.ncbi.nlm.nih.gov/entrez/query.fcgi?db=gene&cmd=Retrieve&dopt=full_report&list_uids=101972038) | Rnf111 | -1.11253 | -2.16224 | 0.001594 | 0.036008 |
| [101972675](https://www.ncbi.nlm.nih.gov/entrez/query.fcgi?db=gene&cmd=Retrieve&dopt=full_report&list_uids=101972675) | Cep170 | -1.11268 | -2.16247 | 0.000347 | 0.011821 |
| [101954686](https://www.ncbi.nlm.nih.gov/entrez/query.fcgi?db=gene&cmd=Retrieve&dopt=full_report&list_uids=101954686) | Xirp1 | -1.1128 | -2.16265 | 0.001795 | 0.039075 |
| [101957557](https://www.ncbi.nlm.nih.gov/entrez/query.fcgi?db=gene&cmd=Retrieve&dopt=full_report&list_uids=101957557) | Dnmbp | -1.11385 | -2.16422 | 0.000485 | 0.01506 |
| [101966750](https://www.ncbi.nlm.nih.gov/entrez/query.fcgi?db=gene&cmd=Retrieve&dopt=full_report&list_uids=101966750) | Acer2 | -1.11921 | -2.17228 | 0.000344 | 0.011749 |
| [101976226](https://www.ncbi.nlm.nih.gov/entrez/query.fcgi?db=gene&cmd=Retrieve&dopt=full_report&list_uids=101976226) | Dpt | -1.11967 | -2.17298 | 0.001727 | 0.037951 |
| [101977068](https://www.ncbi.nlm.nih.gov/entrez/query.fcgi?db=gene&cmd=Retrieve&dopt=full_report&list_uids=101977068) | Net1 | -1.12467 | -2.18051 | 0.000254 | 0.009448 |
| [101973062](https://www.ncbi.nlm.nih.gov/entrez/query.fcgi?db=gene&cmd=Retrieve&dopt=full_report&list_uids=101973062) | Mettl15 | -1.1277 | -2.1851 | 0.000612 | 0.017907 |
| [101959549](https://www.ncbi.nlm.nih.gov/entrez/query.fcgi?db=gene&cmd=Retrieve&dopt=full_report&list_uids=101959549) | LOC101959549 | -1.12778 | -2.18523 | 0.001688 | 0.037427 |
| [101965686](https://www.ncbi.nlm.nih.gov/entrez/query.fcgi?db=gene&cmd=Retrieve&dopt=full_report&list_uids=101965686) | Tsc22d2 | -1.13016 | -2.18883 | 0.000416 | 0.013399 |
| [101969965](https://www.ncbi.nlm.nih.gov/entrez/query.fcgi?db=gene&cmd=Retrieve&dopt=full_report&list_uids=101969965) | Col6a3 | -1.13118 | -2.19038 | 0.002349 | 0.046845 |
| [101963726](https://www.ncbi.nlm.nih.gov/entrez/query.fcgi?db=gene&cmd=Retrieve&dopt=full_report&list_uids=101963726) | Abcc5 | -1.13215 | -2.19185 | 0.000121 | 0.005398 |
| [101975914](https://www.ncbi.nlm.nih.gov/entrez/query.fcgi?db=gene&cmd=Retrieve&dopt=full_report&list_uids=101975914) | Atl2 | -1.1387 | -2.20183 | 0.000141 | 0.006002 |
| [101956290](https://www.ncbi.nlm.nih.gov/entrez/query.fcgi?db=gene&cmd=Retrieve&dopt=full_report&list_uids=101956290) | Ppp1r3c | -1.15007 | -2.21924 | 0.00228 | 0.046069 |
| [101971386](https://www.ncbi.nlm.nih.gov/entrez/query.fcgi?db=gene&cmd=Retrieve&dopt=full_report&list_uids=101971386) | Fam134b | -1.15379 | -2.22497 | 0.000782 | 0.021276 |
| [101974997](https://www.ncbi.nlm.nih.gov/entrez/query.fcgi?db=gene&cmd=Retrieve&dopt=full_report&list_uids=101974997) | Tsc22d1 | -1.15462 | -2.22625 | 5.11E-05 | 0.002597 |
| [101957365](https://www.ncbi.nlm.nih.gov/entrez/query.fcgi?db=gene&cmd=Retrieve&dopt=full_report&list_uids=101957365) | Apold1 | -1.1554 | -2.22745 | 0.000146 | 0.006185 |
| [101972453](https://www.ncbi.nlm.nih.gov/entrez/query.fcgi?db=gene&cmd=Retrieve&dopt=full_report&list_uids=101972453) | Aspn | -1.15757 | -2.23081 | 0.000871 | 0.02288 |
| [101978437](https://www.ncbi.nlm.nih.gov/entrez/query.fcgi?db=gene&cmd=Retrieve&dopt=full_report&list_uids=101978437) | Gas6 | -1.15887 | -2.23283 | 0.000269 | 0.00984 |
| [101978712](https://www.ncbi.nlm.nih.gov/entrez/query.fcgi?db=gene&cmd=Retrieve&dopt=full_report&list_uids=101978712) | Krit1 | -1.16061 | -2.23552 | 0.000333 | 0.011449 |
| [101969000](https://www.ncbi.nlm.nih.gov/entrez/query.fcgi?db=gene&cmd=Retrieve&dopt=full_report&list_uids=101969000) | Six4 | -1.16068 | -2.23563 | 0.000125 | 0.005522 |
| [101967439](https://www.ncbi.nlm.nih.gov/entrez/query.fcgi?db=gene&cmd=Retrieve&dopt=full_report&list_uids=101967439) | Sipa1l2 | -1.16274 | -2.23882 | 0.000651 | 0.018674 |
| [101969616](https://www.ncbi.nlm.nih.gov/entrez/query.fcgi?db=gene&cmd=Retrieve&dopt=full_report&list_uids=101969616) | Klhl9 | -1.16278 | -2.23888 | 0.000132 | 0.005753 |
| [101966799](https://www.ncbi.nlm.nih.gov/entrez/query.fcgi?db=gene&cmd=Retrieve&dopt=full_report&list_uids=101966799) | Nfkbia | -1.16286 | -2.23901 | 6.58E-05 | 0.003211 |
| [101963307](https://www.ncbi.nlm.nih.gov/entrez/query.fcgi?db=gene&cmd=Retrieve&dopt=full_report&list_uids=101963307) | B4galt3 | -1.16376 | -2.2404 | 0.000701 | 0.019748 |
| [101970847](https://www.ncbi.nlm.nih.gov/entrez/query.fcgi?db=gene&cmd=Retrieve&dopt=full_report&list_uids=101970847) | Slc2a4 | -1.16651 | -2.24468 | 0.000162 | 0.006765 |
| [101967168](https://www.ncbi.nlm.nih.gov/entrez/query.fcgi?db=gene&cmd=Retrieve&dopt=full_report&list_uids=101967168) | Tdg | -1.16794 | -2.2469 | 0.000107 | 0.004855 |
| [101969110](https://www.ncbi.nlm.nih.gov/entrez/query.fcgi?db=gene&cmd=Retrieve&dopt=full_report&list_uids=101969110) | Synpo2l | -1.16899 | -2.24854 | 0.00038 | 0.012608 |
| [101975030](https://www.ncbi.nlm.nih.gov/entrez/query.fcgi?db=gene&cmd=Retrieve&dopt=full_report&list_uids=101975030) | Rusc2 | -1.17535 | -2.25848 | 0.000147 | 0.006231 |
| [101958434](https://www.ncbi.nlm.nih.gov/entrez/query.fcgi?db=gene&cmd=Retrieve&dopt=full_report&list_uids=101958434) | Gadl1 | -1.18 | -2.26576 | 0.000299 | 0.010572 |
| [101971911](https://www.ncbi.nlm.nih.gov/entrez/query.fcgi?db=gene&cmd=Retrieve&dopt=full_report&list_uids=101971911) | Dnajb5 | -1.18617 | -2.27547 | 4.83E-05 | 0.002506 |
| [101970688](https://www.ncbi.nlm.nih.gov/entrez/query.fcgi?db=gene&cmd=Retrieve&dopt=full_report&list_uids=101970688) | Cdc42ep3 | -1.18815 | -2.2786 | 0.000169 | 0.006923 |
| [101960834](https://www.ncbi.nlm.nih.gov/entrez/query.fcgi?db=gene&cmd=Retrieve&dopt=full_report&list_uids=101960834) | Homer2 | -1.19142 | -2.28377 | 6.43E-05 | 0.003158 |
| [101965590](https://www.ncbi.nlm.nih.gov/entrez/query.fcgi?db=gene&cmd=Retrieve&dopt=full_report&list_uids=101965590) | Eya3 | -1.19215 | -2.28492 | 3.59E-05 | 0.001978 |
| [101960536](https://www.ncbi.nlm.nih.gov/entrez/query.fcgi?db=gene&cmd=Retrieve&dopt=full_report&list_uids=101960536) | Aasdh | -1.19627 | -2.29147 | 0.000493 | 0.015167 |
| [101974258](https://www.ncbi.nlm.nih.gov/entrez/query.fcgi?db=gene&cmd=Retrieve&dopt=full_report&list_uids=101974258) | Slc19a2 | -1.1988 | -2.29548 | 0.00071 | 0.019911 |
| [101975209](https://www.ncbi.nlm.nih.gov/entrez/query.fcgi?db=gene&cmd=Retrieve&dopt=full_report&list_uids=101975209) | Ets2 | -1.20516 | -2.30562 | 0.000312 | 0.010945 |
| [101959540](https://www.ncbi.nlm.nih.gov/entrez/query.fcgi?db=gene&cmd=Retrieve&dopt=full_report&list_uids=101959540) | Slc3a2 | -1.20713 | -2.30879 | 0.001565 | 0.035514 |
| [101959631](https://www.ncbi.nlm.nih.gov/entrez/query.fcgi?db=gene&cmd=Retrieve&dopt=full_report&list_uids=101959631) | LOC101959631 | -1.20831 | -2.31067 | 1.19E-05 | 0.000771 |
| [101960615](https://www.ncbi.nlm.nih.gov/entrez/query.fcgi?db=gene&cmd=Retrieve&dopt=full_report&list_uids=101960615) | Fam214a | -1.20891 | -2.31162 | 0.001121 | 0.027881 |
| [101969943](https://www.ncbi.nlm.nih.gov/entrez/query.fcgi?db=gene&cmd=Retrieve&dopt=full_report&list_uids=101969943) | Znf280b | -1.21039 | -2.314 | 0.001618 | 0.036208 |
| [101970967](https://www.ncbi.nlm.nih.gov/entrez/query.fcgi?db=gene&cmd=Retrieve&dopt=full_report&list_uids=101970967) | Jmjd7 | -1.21055 | -2.31425 | 0.000543 | 0.016406 |
| [101974624](https://www.ncbi.nlm.nih.gov/entrez/query.fcgi?db=gene&cmd=Retrieve&dopt=full_report&list_uids=101974624) | Gadd45g | -1.21189 | -2.3164 | 0.000285 | 0.010256 |
| [101965563](https://www.ncbi.nlm.nih.gov/entrez/query.fcgi?db=gene&cmd=Retrieve&dopt=full_report&list_uids=101965563) | Mafg | -1.21307 | -2.31831 | 0.000748 | 0.020591 |
| [101967238](https://www.ncbi.nlm.nih.gov/entrez/query.fcgi?db=gene&cmd=Retrieve&dopt=full_report&list_uids=101967238) | Znf280d | -1.21478 | -2.32105 | 3.4E-05 | 0.001894 |
| [101961857](https://www.ncbi.nlm.nih.gov/entrez/query.fcgi?db=gene&cmd=Retrieve&dopt=full_report&list_uids=101961857) | Clk4 | -1.21707 | -2.32474 | 1.48E-05 | 0.000921 |
| [101970968](https://www.ncbi.nlm.nih.gov/entrez/query.fcgi?db=gene&cmd=Retrieve&dopt=full_report&list_uids=101970968) | Fam63b | -1.21813 | -2.32646 | 9.4E-06 | 0.000642 |
| [101958103](https://www.ncbi.nlm.nih.gov/entrez/query.fcgi?db=gene&cmd=Retrieve&dopt=full_report&list_uids=101958103) | Tiparp | -1.21913 | -2.32806 | 0.000268 | 0.00984 |
| [101954875](https://www.ncbi.nlm.nih.gov/entrez/query.fcgi?db=gene&cmd=Retrieve&dopt=full_report&list_uids=101954875) | Pik3ip1 | -1.22097 | -2.33103 | 0.001495 | 0.03441 |
| [101974784](https://www.ncbi.nlm.nih.gov/entrez/query.fcgi?db=gene&cmd=Retrieve&dopt=full_report&list_uids=101974784) | Kbtbd8 | -1.22785 | -2.34218 | 0.00013 | 0.005652 |
| [101958121](https://www.ncbi.nlm.nih.gov/entrez/query.fcgi?db=gene&cmd=Retrieve&dopt=full_report&list_uids=101958121) | Mknk2 | -1.22935 | -2.34461 | 4.23E-06 | 0.000348 |
| [101961929](https://www.ncbi.nlm.nih.gov/entrez/query.fcgi?db=gene&cmd=Retrieve&dopt=full_report&list_uids=101961929) | Ell | -1.23045 | -2.34641 | 9.13E-07 | 8.97E-05 |
| [101977558](https://www.ncbi.nlm.nih.gov/entrez/query.fcgi?db=gene&cmd=Retrieve&dopt=full_report&list_uids=101977558) | Cep95 | -1.23387 | -2.35197 | 0.000162 | 0.006765 |
| [101973922](https://www.ncbi.nlm.nih.gov/entrez/query.fcgi?db=gene&cmd=Retrieve&dopt=full_report&list_uids=101973922) | Smarcd2 | -1.23391 | -2.35204 | 1.54E-05 | 0.000957 |
| [101961352](https://www.ncbi.nlm.nih.gov/entrez/query.fcgi?db=gene&cmd=Retrieve&dopt=full_report&list_uids=101961352) | Abca9 | -1.23862 | -2.35974 | 9.46E-05 | 0.004387 |
| [101978543](https://www.ncbi.nlm.nih.gov/entrez/query.fcgi?db=gene&cmd=Retrieve&dopt=full_report&list_uids=101978543) | Cetp | -1.23877 | -2.35998 | 4.96E-05 | 0.002538 |
| [101966164](https://www.ncbi.nlm.nih.gov/entrez/query.fcgi?db=gene&cmd=Retrieve&dopt=full_report&list_uids=101966164) | Rgs2 | -1.24193 | -2.36514 | 0.002216 | 0.045086 |
| [101978078](https://www.ncbi.nlm.nih.gov/entrez/query.fcgi?db=gene&cmd=Retrieve&dopt=full_report&list_uids=101978078) | Zbtb34 | -1.25923 | -2.39367 | 0.001599 | 0.036061 |
| [101960011](https://www.ncbi.nlm.nih.gov/entrez/query.fcgi?db=gene&cmd=Retrieve&dopt=full_report&list_uids=101960011) | LOC101960011 | -1.26344 | -2.40067 | 0.000146 | 0.006185 |
| [101963280](https://www.ncbi.nlm.nih.gov/entrez/query.fcgi?db=gene&cmd=Retrieve&dopt=full_report&list_uids=101963280) | Ncapd2 | -1.26873 | -2.40949 | 0.002369 | 0.047078 |
| [101966350](https://www.ncbi.nlm.nih.gov/entrez/query.fcgi?db=gene&cmd=Retrieve&dopt=full_report&list_uids=101966350) | Sik2 | -1.27248 | -2.41577 | 0.000176 | 0.007118 |
| [101975348](https://www.ncbi.nlm.nih.gov/entrez/query.fcgi?db=gene&cmd=Retrieve&dopt=full_report&list_uids=101975348) | Col8a1 | -1.29082 | -2.44667 | 0.000806 | 0.021702 |
| [101958547](https://www.ncbi.nlm.nih.gov/entrez/query.fcgi?db=gene&cmd=Retrieve&dopt=full_report&list_uids=101958547) | Lins | -1.29418 | -2.45238 | 0.000925 | 0.02388 |
| [101964370](https://www.ncbi.nlm.nih.gov/entrez/query.fcgi?db=gene&cmd=Retrieve&dopt=full_report&list_uids=101964370) | Susd6 | -1.29592 | -2.45534 | 1.32E-05 | 0.00084 |
| [101972605](https://www.ncbi.nlm.nih.gov/entrez/query.fcgi?db=gene&cmd=Retrieve&dopt=full_report&list_uids=101972605) | Klhl34 | -1.2964 | -2.45616 | 0.001783 | 0.038963 |
| [101958260](https://www.ncbi.nlm.nih.gov/entrez/query.fcgi?db=gene&cmd=Retrieve&dopt=full_report&list_uids=101958260) | Ccm2l | -1.30289 | -2.46723 | 0.002291 | 0.046142 |
| [101972101](https://www.ncbi.nlm.nih.gov/entrez/query.fcgi?db=gene&cmd=Retrieve&dopt=full_report&list_uids=101972101) | Pik3c2a | -1.30715 | -2.47453 | 3.31E-05 | 0.00187 |
| [101958243](https://www.ncbi.nlm.nih.gov/entrez/query.fcgi?db=gene&cmd=Retrieve&dopt=full_report&list_uids=101958243) | Abhd4 | -1.31255 | -2.4838 | 1.04E-05 | 0.00069 |
| [101972020](https://www.ncbi.nlm.nih.gov/entrez/query.fcgi?db=gene&cmd=Retrieve&dopt=full_report&list_uids=101972020) | Crem | -1.3133 | -2.4851 | 1.66E-05 | 0.001018 |
| [101955637](https://www.ncbi.nlm.nih.gov/entrez/query.fcgi?db=gene&cmd=Retrieve&dopt=full_report&list_uids=101955637) | Clk1 | -1.32227 | -2.50058 | 9.06E-07 | 8.97E-05 |
| [101960177](https://www.ncbi.nlm.nih.gov/entrez/query.fcgi?db=gene&cmd=Retrieve&dopt=full_report&list_uids=101960177) | LOC101960177 | -1.32563 | -2.50643 | 0.001567 | 0.035514 |
| [101964079](https://www.ncbi.nlm.nih.gov/entrez/query.fcgi?db=gene&cmd=Retrieve&dopt=full_report&list_uids=101964079) | Hey1 | -1.32651 | -2.50795 | 5.99E-05 | 0.003005 |
| [101975322](https://www.ncbi.nlm.nih.gov/entrez/query.fcgi?db=gene&cmd=Retrieve&dopt=full_report&list_uids=101975322) | Tfdp2 | -1.32723 | -2.5092 | 1.42E-05 | 0.000893 |
| [101964669](https://www.ncbi.nlm.nih.gov/entrez/query.fcgi?db=gene&cmd=Retrieve&dopt=full_report&list_uids=101964669) | Hspb1 | -1.32734 | -2.50939 | 0.000176 | 0.007118 |
| [101954660](https://www.ncbi.nlm.nih.gov/entrez/query.fcgi?db=gene&cmd=Retrieve&dopt=full_report&list_uids=101954660) | Rapgef2 | -1.32758 | -2.50982 | 0.001005 | 0.02547 |
| [101978190](https://www.ncbi.nlm.nih.gov/entrez/query.fcgi?db=gene&cmd=Retrieve&dopt=full_report&list_uids=101978190) | Pde7a | -1.33219 | -2.51784 | 0.000329 | 0.011396 |
| [101956305](https://www.ncbi.nlm.nih.gov/entrez/query.fcgi?db=gene&cmd=Retrieve&dopt=full_report&list_uids=101956305) | Cep85l | -1.336 | -2.52451 | 0.000825 | 0.021863 |
| [101956546](https://www.ncbi.nlm.nih.gov/entrez/query.fcgi?db=gene&cmd=Retrieve&dopt=full_report&list_uids=101956546) | Xdh | -1.34026 | -2.53197 | 0.000726 | 0.020198 |
| [101957267](https://www.ncbi.nlm.nih.gov/entrez/query.fcgi?db=gene&cmd=Retrieve&dopt=full_report&list_uids=101957267) | Zbtb16 | -1.3431 | -2.53696 | 1.06E-05 | 0.000698 |
| [101957501](https://www.ncbi.nlm.nih.gov/entrez/query.fcgi?db=gene&cmd=Retrieve&dopt=full_report&list_uids=101957501) | Gpcpd1 | -1.34335 | -2.5374 | 3.73E-06 | 0.000309 |
| [101955242](https://www.ncbi.nlm.nih.gov/entrez/query.fcgi?db=gene&cmd=Retrieve&dopt=full_report&list_uids=101955242) | LOC101955242 | -1.35415 | -2.55646 | 7.56E-07 | 7.61E-05 |
| [101966787](https://www.ncbi.nlm.nih.gov/entrez/query.fcgi?db=gene&cmd=Retrieve&dopt=full_report&list_uids=101966787) | Ier3 | -1.35667 | -2.56093 | 0.000821 | 0.021863 |
| [101968165](https://www.ncbi.nlm.nih.gov/entrez/query.fcgi?db=gene&cmd=Retrieve&dopt=full_report&list_uids=101968165) | Bicc1 | -1.35788 | -2.56308 | 0.000269 | 0.00984 |
| [101955324](https://www.ncbi.nlm.nih.gov/entrez/query.fcgi?db=gene&cmd=Retrieve&dopt=full_report&list_uids=101955324) | Fkrp | -1.36097 | -2.56858 | 0.000126 | 0.005543 |
| [101962491](https://www.ncbi.nlm.nih.gov/entrez/query.fcgi?db=gene&cmd=Retrieve&dopt=full_report&list_uids=101962491) | Actg2 | -1.36513 | -2.576 | 0.002378 | 0.047181 |
| [101958711](https://www.ncbi.nlm.nih.gov/entrez/query.fcgi?db=gene&cmd=Retrieve&dopt=full_report&list_uids=101958711) | LOC101958711 | -1.36691 | -2.57918 | 1.19E-06 | 0.000111 |
| [101978217](https://www.ncbi.nlm.nih.gov/entrez/query.fcgi?db=gene&cmd=Retrieve&dopt=full_report&list_uids=101978217) | Dnajb4 | -1.37579 | -2.59509 | 6.92E-06 | 0.000501 |
| [101976351](https://www.ncbi.nlm.nih.gov/entrez/query.fcgi?db=gene&cmd=Retrieve&dopt=full_report&list_uids=101976351) | Tp53inp2 | -1.37715 | -2.59754 | 7.27E-06 | 0.000517 |
| [101961480](https://www.ncbi.nlm.nih.gov/entrez/query.fcgi?db=gene&cmd=Retrieve&dopt=full_report&list_uids=101961480) | Sh2d7 | -1.3815 | -2.6054 | 7.37E-06 | 0.000521 |
| [101968355](https://www.ncbi.nlm.nih.gov/entrez/query.fcgi?db=gene&cmd=Retrieve&dopt=full_report&list_uids=101968355) | Taf5 | -1.38189 | -2.6061 | 0.00218 | 0.04457 |
| [101975694](https://www.ncbi.nlm.nih.gov/entrez/query.fcgi?db=gene&cmd=Retrieve&dopt=full_report&list_uids=101975694) | Msl2 | -1.38694 | -2.61523 | 4.42E-06 | 0.000355 |
| [101954362](https://www.ncbi.nlm.nih.gov/entrez/query.fcgi?db=gene&cmd=Retrieve&dopt=full_report&list_uids=101954362) | LOC101954362 | -1.39041 | -2.62153 | 0.000136 | 0.005828 |
| [101965480](https://www.ncbi.nlm.nih.gov/entrez/query.fcgi?db=gene&cmd=Retrieve&dopt=full_report&list_uids=101965480) | Azin1 | -1.40346 | -2.64535 | 6.29E-06 | 0.000464 |
| [101961677](https://www.ncbi.nlm.nih.gov/entrez/query.fcgi?db=gene&cmd=Retrieve&dopt=full_report&list_uids=101961677) | Lonrf3 | -1.40427 | -2.64683 | 0.001091 | 0.027232 |
| [101954349](https://www.ncbi.nlm.nih.gov/entrez/query.fcgi?db=gene&cmd=Retrieve&dopt=full_report&list_uids=101954349) | Kcnma1 | -1.40578 | -2.64961 | 0.001381 | 0.032588 |
| [101958010](https://www.ncbi.nlm.nih.gov/entrez/query.fcgi?db=gene&cmd=Retrieve&dopt=full_report&list_uids=101958010) | Map3k14 | -1.41682 | -2.66997 | 0.000106 | 0.004812 |
| [101977713](https://www.ncbi.nlm.nih.gov/entrez/query.fcgi?db=gene&cmd=Retrieve&dopt=full_report&list_uids=101977713) | Mybpc2 | -1.42121 | -2.67811 | 0.001791 | 0.039065 |
| [101962987](https://www.ncbi.nlm.nih.gov/entrez/query.fcgi?db=gene&cmd=Retrieve&dopt=full_report&list_uids=101962987) | Rbms2 | -1.42706 | -2.68898 | 6.13E-07 | 6.34E-05 |
| [101971147](https://www.ncbi.nlm.nih.gov/entrez/query.fcgi?db=gene&cmd=Retrieve&dopt=full_report&list_uids=101971147) | Kcnj2 | -1.42737 | -2.68956 | 1.26E-05 | 0.000807 |
| [101970786](https://www.ncbi.nlm.nih.gov/entrez/query.fcgi?db=gene&cmd=Retrieve&dopt=full_report&list_uids=101970786) | B3galnt1 | -1.42793 | -2.6906 | 0.000454 | 0.014411 |
| [101978361](https://www.ncbi.nlm.nih.gov/entrez/query.fcgi?db=gene&cmd=Retrieve&dopt=full_report&list_uids=101978361) | Ppfibp2 | -1.44315 | -2.71914 | 0.000175 | 0.007118 |
| [101956940](https://www.ncbi.nlm.nih.gov/entrez/query.fcgi?db=gene&cmd=Retrieve&dopt=full_report&list_uids=101956940) | Fzd4 | -1.44333 | -2.71948 | 6.34E-05 | 0.003141 |
| [101955532](https://www.ncbi.nlm.nih.gov/entrez/query.fcgi?db=gene&cmd=Retrieve&dopt=full_report&list_uids=101955532) | Znf526 | -1.45769 | -2.74667 | 7.66E-06 | 0.000538 |
| [101963881](https://www.ncbi.nlm.nih.gov/entrez/query.fcgi?db=gene&cmd=Retrieve&dopt=full_report&list_uids=101963881) | Dusp8 | -1.46126 | -2.75349 | 8.29E-06 | 0.000569 |
| [101964136](https://www.ncbi.nlm.nih.gov/entrez/query.fcgi?db=gene&cmd=Retrieve&dopt=full_report&list_uids=101964136) | Sec24a | -1.46397 | -2.75866 | 0.00035 | 0.011885 |
| [101959952](https://www.ncbi.nlm.nih.gov/entrez/query.fcgi?db=gene&cmd=Retrieve&dopt=full_report&list_uids=101959952) | Dph2 | -1.47209 | -2.77423 | 0.002398 | 0.04735 |
| [101966521](https://www.ncbi.nlm.nih.gov/entrez/query.fcgi?db=gene&cmd=Retrieve&dopt=full_report&list_uids=101966521) | Zfand5 | -1.47769 | -2.78503 | 2.94E-07 | 3.34E-05 |
| [101970407](https://www.ncbi.nlm.nih.gov/entrez/query.fcgi?db=gene&cmd=Retrieve&dopt=full_report&list_uids=101970407) | Igdcc4 | -1.47893 | -2.78742 | 0.002306 | 0.046378 |
| [101968523](https://www.ncbi.nlm.nih.gov/entrez/query.fcgi?db=gene&cmd=Retrieve&dopt=full_report&list_uids=101968523) | Kctd21 | -1.48384 | -2.79692 | 0.00243 | 0.04768 |
| [101977782](https://www.ncbi.nlm.nih.gov/entrez/query.fcgi?db=gene&cmd=Retrieve&dopt=full_report&list_uids=101977782) | Tinagl1 | -1.48491 | -2.799 | 1.1E-07 | 1.43E-05 |
| [101972491](https://www.ncbi.nlm.nih.gov/entrez/query.fcgi?db=gene&cmd=Retrieve&dopt=full_report&list_uids=101972491) | Tmem140 | -1.48551 | -2.80016 | 7.03E-05 | 0.003399 |
| [101978320](https://www.ncbi.nlm.nih.gov/entrez/query.fcgi?db=gene&cmd=Retrieve&dopt=full_report&list_uids=101978320) | Acss1 | -1.48632 | -2.80174 | 2.36E-05 | 0.001384 |
| [101961235](https://www.ncbi.nlm.nih.gov/entrez/query.fcgi?db=gene&cmd=Retrieve&dopt=full_report&list_uids=101961235) | LOC101961235 | -1.48762 | -2.80426 | 1.29E-06 | 0.000119 |
| [101974197](https://www.ncbi.nlm.nih.gov/entrez/query.fcgi?db=gene&cmd=Retrieve&dopt=full_report&list_uids=101974197) | Ddit4l | -1.48964 | -2.80818 | 4.34E-05 | 0.002322 |
| [101955624](https://www.ncbi.nlm.nih.gov/entrez/query.fcgi?db=gene&cmd=Retrieve&dopt=full_report&list_uids=101955624) | LOC101955624 | -1.49011 | -2.8091 | 3.54E-05 | 0.001965 |
| [106145291](https://www.ncbi.nlm.nih.gov/entrez/query.fcgi?db=gene&cmd=Retrieve&dopt=full_report&list_uids=106145291) | LOC106145291 | -1.49219 | -2.81315 | 2.68E-06 | 0.000227 |
| [101955906](https://www.ncbi.nlm.nih.gov/entrez/query.fcgi?db=gene&cmd=Retrieve&dopt=full_report&list_uids=101955906) | Fzd1 | -1.49581 | -2.82022 | 4.74E-06 | 0.000375 |
| [101978484](https://www.ncbi.nlm.nih.gov/entrez/query.fcgi?db=gene&cmd=Retrieve&dopt=full_report&list_uids=101978484) | Chst7 | -1.49844 | -2.82537 | 0.000746 | 0.020581 |
| [101973209](https://www.ncbi.nlm.nih.gov/entrez/query.fcgi?db=gene&cmd=Retrieve&dopt=full_report&list_uids=101973209) | Ogn | -1.49954 | -2.82753 | 0.000425 | 0.013613 |
| [101957463](https://www.ncbi.nlm.nih.gov/entrez/query.fcgi?db=gene&cmd=Retrieve&dopt=full_report&list_uids=101957463) | Pnpla8 | -1.50391 | -2.8361 | 0.000712 | 0.019919 |
| [101976994](https://www.ncbi.nlm.nih.gov/entrez/query.fcgi?db=gene&cmd=Retrieve&dopt=full_report&list_uids=101976994) | LOC101976994 | -1.51369 | -2.8554 | 9.47E-06 | 0.000643 |
| [101957276](https://www.ncbi.nlm.nih.gov/entrez/query.fcgi?db=gene&cmd=Retrieve&dopt=full_report&list_uids=101957276) | Zfp36 | -1.51975 | -2.86741 | 3.99E-09 | 6.89E-07 |
| [101973720](https://www.ncbi.nlm.nih.gov/entrez/query.fcgi?db=gene&cmd=Retrieve&dopt=full_report&list_uids=101973720) | Rapgef3 | -1.52155 | -2.87099 | 5.98E-06 | 0.000443 |
| [101961069](https://www.ncbi.nlm.nih.gov/entrez/query.fcgi?db=gene&cmd=Retrieve&dopt=full_report&list_uids=101961069) | Lonrf1 | -1.52665 | -2.88117 | 0.002508 | 0.04849 |
| [101967013](https://www.ncbi.nlm.nih.gov/entrez/query.fcgi?db=gene&cmd=Retrieve&dopt=full_report&list_uids=101967013) | Thbs3 | -1.52962 | -2.8871 | 0.002429 | 0.04768 |
| [102314766](https://www.ncbi.nlm.nih.gov/entrez/query.fcgi?db=gene&cmd=Retrieve&dopt=full_report&list_uids=102314766) | Ak6 | -1.53786 | -2.90363 | 1.03E-05 | 0.000686 |
| [101967668](https://www.ncbi.nlm.nih.gov/entrez/query.fcgi?db=gene&cmd=Retrieve&dopt=full_report&list_uids=101967668) | Cntfr | -1.54302 | -2.91403 | 0.001887 | 0.040434 |
| [101969382](https://www.ncbi.nlm.nih.gov/entrez/query.fcgi?db=gene&cmd=Retrieve&dopt=full_report&list_uids=101969382) | LOC101969382 | -1.54923 | -2.9266 | 1.73E-06 | 0.000157 |
| [101964036](https://www.ncbi.nlm.nih.gov/entrez/query.fcgi?db=gene&cmd=Retrieve&dopt=full_report&list_uids=101964036) | Arid5b | -1.55447 | -2.93725 | 8.25E-06 | 0.000569 |
| [101961599](https://www.ncbi.nlm.nih.gov/entrez/query.fcgi?db=gene&cmd=Retrieve&dopt=full_report&list_uids=101961599) | Fkbp14 | -1.55529 | -2.93894 | 0.002448 | 0.047722 |
| [101968164](https://www.ncbi.nlm.nih.gov/entrez/query.fcgi?db=gene&cmd=Retrieve&dopt=full_report&list_uids=101968164) | LOC101968164 | -1.55852 | -2.94551 | 0.000127 | 0.005546 |
| [101964605](https://www.ncbi.nlm.nih.gov/entrez/query.fcgi?db=gene&cmd=Retrieve&dopt=full_report&list_uids=101964605) | Traf3ip2 | -1.56145 | -2.95151 | 0.000361 | 0.012165 |
| [101971536](https://www.ncbi.nlm.nih.gov/entrez/query.fcgi?db=gene&cmd=Retrieve&dopt=full_report&list_uids=101971536) | Spc24 | -1.56175 | -2.95212 | 0.000688 | 0.019423 |
| [101958679](https://www.ncbi.nlm.nih.gov/entrez/query.fcgi?db=gene&cmd=Retrieve&dopt=full_report&list_uids=101958679) | Gna13 | -1.56952 | -2.96805 | 1.52E-07 | 1.84E-05 |
| [101978226](https://www.ncbi.nlm.nih.gov/entrez/query.fcgi?db=gene&cmd=Retrieve&dopt=full_report&list_uids=101978226) | Ubtd2 | -1.57849 | -2.98657 | 0.000898 | 0.023369 |
| [101955029](https://www.ncbi.nlm.nih.gov/entrez/query.fcgi?db=gene&cmd=Retrieve&dopt=full_report&list_uids=101955029) | LOC101955029 | -1.57874 | -2.98708 | 0.002163 | 0.044303 |
| [101964227](https://www.ncbi.nlm.nih.gov/entrez/query.fcgi?db=gene&cmd=Retrieve&dopt=full_report&list_uids=101964227) | Nova2 | -1.58233 | -2.99454 | 0.002427 | 0.04768 |
| [101973729](https://www.ncbi.nlm.nih.gov/entrez/query.fcgi?db=gene&cmd=Retrieve&dopt=full_report&list_uids=101973729) | Bud13 | -1.5831 | -2.99614 | 5.92E-05 | 0.00298 |
| [101967146](https://www.ncbi.nlm.nih.gov/entrez/query.fcgi?db=gene&cmd=Retrieve&dopt=full_report&list_uids=101967146) | Bcorl1 | -1.5835 | -2.99696 | 0.00068 | 0.019292 |
| [101956759](https://www.ncbi.nlm.nih.gov/entrez/query.fcgi?db=gene&cmd=Retrieve&dopt=full_report&list_uids=101956759) | Slc43a2 | -1.58686 | -3.00395 | 5.08E-06 | 0.000394 |
| [101977094](https://www.ncbi.nlm.nih.gov/entrez/query.fcgi?db=gene&cmd=Retrieve&dopt=full_report&list_uids=101977094) | Lca5 | -1.58838 | -3.00712 | 0.002312 | 0.04641 |
| [101967490](https://www.ncbi.nlm.nih.gov/entrez/query.fcgi?db=gene&cmd=Retrieve&dopt=full_report&list_uids=101967490) | Foxo1 | -1.60583 | -3.04372 | 2.91E-07 | 3.34E-05 |
| [101970591](https://www.ncbi.nlm.nih.gov/entrez/query.fcgi?db=gene&cmd=Retrieve&dopt=full_report&list_uids=101970591) | Tmem150a | -1.6105 | -3.05357 | 2.03E-09 | 3.61E-07 |
| [101966793](https://www.ncbi.nlm.nih.gov/entrez/query.fcgi?db=gene&cmd=Retrieve&dopt=full_report&list_uids=101966793) | LOC101966793 | -1.61444 | -3.06194 | 0.000776 | 0.02118 |
| [101962188](https://www.ncbi.nlm.nih.gov/entrez/query.fcgi?db=gene&cmd=Retrieve&dopt=full_report&list_uids=101962188) | Gemin6 | -1.61981 | -3.07335 | 0.001751 | 0.038397 |
| [101958044](https://www.ncbi.nlm.nih.gov/entrez/query.fcgi?db=gene&cmd=Retrieve&dopt=full_report&list_uids=101958044) | Abra | -1.62728 | -3.08929 | 4.15E-05 | 0.00223 |
| [101975939](https://www.ncbi.nlm.nih.gov/entrez/query.fcgi?db=gene&cmd=Retrieve&dopt=full_report&list_uids=101975939) | Pfkfb1 | -1.62756 | -3.0899 | 2.72E-08 | 3.97E-06 |
| [101978068](https://www.ncbi.nlm.nih.gov/entrez/query.fcgi?db=gene&cmd=Retrieve&dopt=full_report&list_uids=101978068) | Rasl11b | -1.62827 | -3.09143 | 0.001147 | 0.028375 |
| [101977187](https://www.ncbi.nlm.nih.gov/entrez/query.fcgi?db=gene&cmd=Retrieve&dopt=full_report&list_uids=101977187) | Tmco6 | -1.62906 | -3.09312 | 0.00084 | 0.022153 |
| [101975334](https://www.ncbi.nlm.nih.gov/entrez/query.fcgi?db=gene&cmd=Retrieve&dopt=full_report&list_uids=101975334) | Znf599 | -1.63707 | -3.11034 | 0.00207 | 0.042906 |
| [101972672](https://www.ncbi.nlm.nih.gov/entrez/query.fcgi?db=gene&cmd=Retrieve&dopt=full_report&list_uids=101972672) | Zswim3 | -1.63925 | -3.11505 | 0.000596 | 0.017571 |
| [101959146](https://www.ncbi.nlm.nih.gov/entrez/query.fcgi?db=gene&cmd=Retrieve&dopt=full_report&list_uids=101959146) | Arl5b | -1.64987 | -3.13806 | 6.62E-05 | 0.003211 |
| [101954857](https://www.ncbi.nlm.nih.gov/entrez/query.fcgi?db=gene&cmd=Retrieve&dopt=full_report&list_uids=101954857) | LOC101954857 | -1.65533 | -3.14996 | 1.74E-06 | 0.000157 |
| [101977859](https://www.ncbi.nlm.nih.gov/entrez/query.fcgi?db=gene&cmd=Retrieve&dopt=full_report&list_uids=101977859) | Actn3 | -1.67328 | -3.18939 | 5.52E-05 | 0.00279 |
| [101959477](https://www.ncbi.nlm.nih.gov/entrez/query.fcgi?db=gene&cmd=Retrieve&dopt=full_report&list_uids=101959477) | Mylk3 | -1.67357 | -3.19003 | 0.001543 | 0.035191 |
| [101957921](https://www.ncbi.nlm.nih.gov/entrez/query.fcgi?db=gene&cmd=Retrieve&dopt=full_report&list_uids=101957921) | Foxo3 | -1.69692 | -3.24208 | 7.94E-07 | 7.93E-05 |
| [101965304](https://www.ncbi.nlm.nih.gov/entrez/query.fcgi?db=gene&cmd=Retrieve&dopt=full_report&list_uids=101965304) | Dgkh | -1.69699 | -3.24224 | 0.0005 | 0.015308 |
| [101975260](https://www.ncbi.nlm.nih.gov/entrez/query.fcgi?db=gene&cmd=Retrieve&dopt=full_report&list_uids=101975260) | Calml4 | -1.69836 | -3.24532 | 0.001347 | 0.032122 |
| [101971504](https://www.ncbi.nlm.nih.gov/entrez/query.fcgi?db=gene&cmd=Retrieve&dopt=full_report&list_uids=101971504) | Atp6v1c2 | -1.70657 | -3.26383 | 0.001239 | 0.030249 |
| [101957188](https://www.ncbi.nlm.nih.gov/entrez/query.fcgi?db=gene&cmd=Retrieve&dopt=full_report&list_uids=101957188) | Tnfrsf12a | -1.70977 | -3.27109 | 1.21E-05 | 0.000778 |
| [101966159](https://www.ncbi.nlm.nih.gov/entrez/query.fcgi?db=gene&cmd=Retrieve&dopt=full_report&list_uids=101966159) | Parp11 | -1.72349 | -3.30234 | 0.00011 | 0.004937 |
| [101969046](https://www.ncbi.nlm.nih.gov/entrez/query.fcgi?db=gene&cmd=Retrieve&dopt=full_report&list_uids=101969046) | Gpatch2 | -1.72748 | -3.31148 | 0.000391 | 0.012797 |
| [101977521](https://www.ncbi.nlm.nih.gov/entrez/query.fcgi?db=gene&cmd=Retrieve&dopt=full_report&list_uids=101977521) | Sema4b | -1.72991 | -3.31708 | 6.55E-10 | 1.34E-07 |
| [101963592](https://www.ncbi.nlm.nih.gov/entrez/query.fcgi?db=gene&cmd=Retrieve&dopt=full_report&list_uids=101963592) | Klf15 | -1.74767 | -3.35816 | 0.000389 | 0.012797 |
| [101968459](https://www.ncbi.nlm.nih.gov/entrez/query.fcgi?db=gene&cmd=Retrieve&dopt=full_report&list_uids=101968459) | Wbp1l | -1.74993 | -3.36341 | 1.3E-07 | 1.63E-05 |
| [101955615](https://www.ncbi.nlm.nih.gov/entrez/query.fcgi?db=gene&cmd=Retrieve&dopt=full_report&list_uids=101955615) | Slc16a7 | -1.74995 | -3.36348 | 2.48E-05 | 0.001425 |
| [101958764](https://www.ncbi.nlm.nih.gov/entrez/query.fcgi?db=gene&cmd=Retrieve&dopt=full_report&list_uids=101958764) | Slc38a2 | -1.75045 | -3.36463 | 4.37E-07 | 4.63E-05 |
| [101966157](https://www.ncbi.nlm.nih.gov/entrez/query.fcgi?db=gene&cmd=Retrieve&dopt=full_report&list_uids=101966157) | Sh3pxd2a | -1.76795 | -3.4057 | 1.05E-05 | 0.000696 |
| [101956680](https://www.ncbi.nlm.nih.gov/entrez/query.fcgi?db=gene&cmd=Retrieve&dopt=full_report&list_uids=101956680) | Baiap2 | -1.77222 | -3.41579 | 4.46E-05 | 0.00235 |
| [101977049](https://www.ncbi.nlm.nih.gov/entrez/query.fcgi?db=gene&cmd=Retrieve&dopt=full_report&list_uids=101977049) | Trim63 | -1.77364 | -3.41917 | 9.4E-05 | 0.004387 |
| [101954312](https://www.ncbi.nlm.nih.gov/entrez/query.fcgi?db=gene&cmd=Retrieve&dopt=full_report&list_uids=101954312) | LOC101954312 | -1.84566 | -3.59418 | 0.000682 | 0.019309 |
| [101956590](https://www.ncbi.nlm.nih.gov/entrez/query.fcgi?db=gene&cmd=Retrieve&dopt=full_report&list_uids=101956590) | Adamts1 | -1.85158 | -3.60895 | 1.96E-05 | 0.001176 |
| [101967602](https://www.ncbi.nlm.nih.gov/entrez/query.fcgi?db=gene&cmd=Retrieve&dopt=full_report&list_uids=101967602) | Banp | -1.85908 | -3.62775 | 1.39E-05 | 0.000878 |
| [101977384](https://www.ncbi.nlm.nih.gov/entrez/query.fcgi?db=gene&cmd=Retrieve&dopt=full_report&list_uids=101977384) | Ccdc28a | -1.8595 | -3.62883 | 0.00041 | 0.01325 |
| [101960258](https://www.ncbi.nlm.nih.gov/entrez/query.fcgi?db=gene&cmd=Retrieve&dopt=full_report&list_uids=101960258) | Smox | -1.86602 | -3.64525 | 2.06E-05 | 0.001218 |
| [101970884](https://www.ncbi.nlm.nih.gov/entrez/query.fcgi?db=gene&cmd=Retrieve&dopt=full_report&list_uids=101970884) | Glul | -1.87115 | -3.65825 | 5.4E-10 | 1.14E-07 |
| [101968061](https://www.ncbi.nlm.nih.gov/entrez/query.fcgi?db=gene&cmd=Retrieve&dopt=full_report&list_uids=101968061) | Ndel1 | -1.87722 | -3.67366 | 1.17E-10 | 3.07E-08 |
| [101967491](https://www.ncbi.nlm.nih.gov/entrez/query.fcgi?db=gene&cmd=Retrieve&dopt=full_report&list_uids=101967491) | Wtip | -1.87761 | -3.67465 | 7.4E-05 | 0.003551 |
| [101978616](https://www.ncbi.nlm.nih.gov/entrez/query.fcgi?db=gene&cmd=Retrieve&dopt=full_report&list_uids=101978616) | Casq2 | -1.90068 | -3.7339 | 0.000295 | 0.010468 |
| [101964142](https://www.ncbi.nlm.nih.gov/entrez/query.fcgi?db=gene&cmd=Retrieve&dopt=full_report&list_uids=101964142) | Chac1 | -1.90693 | -3.75011 | 0.001238 | 0.030249 |
| [101962368](https://www.ncbi.nlm.nih.gov/entrez/query.fcgi?db=gene&cmd=Retrieve&dopt=full_report&list_uids=101962368) | Slc25a25 | -1.90735 | -3.75119 | 9.29E-10 | 1.78E-07 |
| [101962985](https://www.ncbi.nlm.nih.gov/entrez/query.fcgi?db=gene&cmd=Retrieve&dopt=full_report&list_uids=101962985) | Adamts9 | -1.91055 | -3.75951 | 0.000236 | 0.008877 |
| [101956443](https://www.ncbi.nlm.nih.gov/entrez/query.fcgi?db=gene&cmd=Retrieve&dopt=full_report&list_uids=101956443) | Rcan1 | -1.92283 | -3.79166 | 9.87E-07 | 9.54E-05 |
| [101978630](https://www.ncbi.nlm.nih.gov/entrez/query.fcgi?db=gene&cmd=Retrieve&dopt=full_report&list_uids=101978630) | Cep85 | -1.92344 | -3.79326 | 3.8E-11 | 1.14E-08 |
| [101959177](https://www.ncbi.nlm.nih.gov/entrez/query.fcgi?db=gene&cmd=Retrieve&dopt=full_report&list_uids=101959177) | LOC101959177 | -1.92624 | -3.80065 | 0.001219 | 0.030007 |
| [101958857](https://www.ncbi.nlm.nih.gov/entrez/query.fcgi?db=gene&cmd=Retrieve&dopt=full_report&list_uids=101958857) | Col3a1 | -1.9386 | -3.83333 | 0.000177 | 0.007118 |
| [101958963](https://www.ncbi.nlm.nih.gov/entrez/query.fcgi?db=gene&cmd=Retrieve&dopt=full_report&list_uids=101958963) | LOC101958963 | -1.94324 | -3.8457 | 7.47E-12 | 2.51E-09 |
| [101956810](https://www.ncbi.nlm.nih.gov/entrez/query.fcgi?db=gene&cmd=Retrieve&dopt=full_report&list_uids=101956810) | Fam126b | -1.95035 | -3.86467 | 2.61E-05 | 0.001494 |
| [101967021](https://www.ncbi.nlm.nih.gov/entrez/query.fcgi?db=gene&cmd=Retrieve&dopt=full_report&list_uids=101967021) | Pde4c | -1.97404 | -3.92868 | 0.000167 | 0.006902 |
| [101966425](https://www.ncbi.nlm.nih.gov/entrez/query.fcgi?db=gene&cmd=Retrieve&dopt=full_report&list_uids=101966425) | Myod1 | -1.97454 | -3.93003 | 9.15E-05 | 0.004288 |
| [101965112](https://www.ncbi.nlm.nih.gov/entrez/query.fcgi?db=gene&cmd=Retrieve&dopt=full_report&list_uids=101965112) | Klhl25 | -1.9954 | -3.98727 | 3.7E-12 | 1.31E-09 |
| [101954518](https://www.ncbi.nlm.nih.gov/entrez/query.fcgi?db=gene&cmd=Retrieve&dopt=full_report&list_uids=101954518) | Tob2 | -1.9978 | -3.9939 | 1.02E-12 | 4.25E-10 |
| [101973557](https://www.ncbi.nlm.nih.gov/entrez/query.fcgi?db=gene&cmd=Retrieve&dopt=full_report&list_uids=101973557) | Slc2a13 | -2.00165 | -4.00459 | 0.00246 | 0.047722 |
| [101957870](https://www.ncbi.nlm.nih.gov/entrez/query.fcgi?db=gene&cmd=Retrieve&dopt=full_report&list_uids=101957870) | Bcl2l11 | -2.01189 | -4.03311 | 1.88E-05 | 0.001139 |
| [101961009](https://www.ncbi.nlm.nih.gov/entrez/query.fcgi?db=gene&cmd=Retrieve&dopt=full_report&list_uids=101961009) | Cib2 | -2.01639 | -4.04569 | 4.14E-10 | 9.44E-08 |
| [101970527](https://www.ncbi.nlm.nih.gov/entrez/query.fcgi?db=gene&cmd=Retrieve&dopt=full_report&list_uids=101970527) | Bcl9 | -2.03381 | -4.09485 | 2.49E-06 | 0.000212 |
| [101976785](https://www.ncbi.nlm.nih.gov/entrez/query.fcgi?db=gene&cmd=Retrieve&dopt=full_report&list_uids=101976785) | LOC101976785 | -2.05701 | -4.16122 | 7.06E-08 | 9.37E-06 |
| [101959724](https://www.ncbi.nlm.nih.gov/entrez/query.fcgi?db=gene&cmd=Retrieve&dopt=full_report&list_uids=101959724) | Sesn1 | -2.07045 | -4.20018 | 1.81E-10 | 4.55E-08 |
| [106144852](https://www.ncbi.nlm.nih.gov/entrez/query.fcgi?db=gene&cmd=Retrieve&dopt=full_report&list_uids=106144852) | LOC106144852 | -2.07236 | -4.20575 | 1.87E-06 | 0.000164 |
| [101965763](https://www.ncbi.nlm.nih.gov/entrez/query.fcgi?db=gene&cmd=Retrieve&dopt=full_report&list_uids=101965763) | Ulk1 | -2.07254 | -4.20625 | 3.25E-08 | 4.67E-06 |
| [101965076](https://www.ncbi.nlm.nih.gov/entrez/query.fcgi?db=gene&cmd=Retrieve&dopt=full_report&list_uids=101965076) | Arrdc3 | -2.0769 | -4.219 | 7.49E-13 | 3.35E-10 |
| [101957577](https://www.ncbi.nlm.nih.gov/entrez/query.fcgi?db=gene&cmd=Retrieve&dopt=full_report&list_uids=101957577) | Cflar | -2.0861 | -4.246 | 1.02E-12 | 4.25E-10 |
| [101974277](https://www.ncbi.nlm.nih.gov/entrez/query.fcgi?db=gene&cmd=Retrieve&dopt=full_report&list_uids=101974277) | Hecw2 | -2.08642 | -4.24693 | 4.14E-07 | 4.46E-05 |
| [101976907](https://www.ncbi.nlm.nih.gov/entrez/query.fcgi?db=gene&cmd=Retrieve&dopt=full_report&list_uids=101976907) | Mical2 | -2.09074 | -4.25967 | 7.84E-09 | 1.3E-06 |
| [101965957](https://www.ncbi.nlm.nih.gov/entrez/query.fcgi?db=gene&cmd=Retrieve&dopt=full_report&list_uids=101965957) | LOC101965957 | -2.10299 | -4.29599 | 4.63E-08 | 6.36E-06 |
| [101960347](https://www.ncbi.nlm.nih.gov/entrez/query.fcgi?db=gene&cmd=Retrieve&dopt=full_report&list_uids=101960347) | Btnl9 | -2.11238 | -4.32403 | 6.48E-10 | 1.34E-07 |
| [101963637](https://www.ncbi.nlm.nih.gov/entrez/query.fcgi?db=gene&cmd=Retrieve&dopt=full_report&list_uids=101963637) | Per1 | -2.12639 | -4.36624 | 7.71E-11 | 2.12E-08 |
| [101954990](https://www.ncbi.nlm.nih.gov/entrez/query.fcgi?db=gene&cmd=Retrieve&dopt=full_report&list_uids=101954990) | LOC101954990 | -2.13399 | -4.38929 | 2.21E-07 | 2.56E-05 |
| [101962514](https://www.ncbi.nlm.nih.gov/entrez/query.fcgi?db=gene&cmd=Retrieve&dopt=full_report&list_uids=101962514) | Klhl38 | -2.15346 | -4.44893 | 2E-09 | 3.61E-07 |
| [101955139](https://www.ncbi.nlm.nih.gov/entrez/query.fcgi?db=gene&cmd=Retrieve&dopt=full_report&list_uids=101955139) | Ppm1k | -2.15572 | -4.45592 | 5.67E-06 | 0.000424 |
| [101959575](https://www.ncbi.nlm.nih.gov/entrez/query.fcgi?db=gene&cmd=Retrieve&dopt=full_report&list_uids=101959575) | Fgd2 | -2.16606 | -4.48795 | 5.56E-06 | 0.000423 |
| [101960532](https://www.ncbi.nlm.nih.gov/entrez/query.fcgi?db=gene&cmd=Retrieve&dopt=full_report&list_uids=101960532) | Slc25a43 | -2.17836 | -4.52639 | 0.000194 | 0.007579 |
| [101956173](https://www.ncbi.nlm.nih.gov/entrez/query.fcgi?db=gene&cmd=Retrieve&dopt=full_report&list_uids=101956173) | Ampd3 | -2.18904 | -4.56001 | 1.45E-07 | 1.79E-05 |
| [101958055](https://www.ncbi.nlm.nih.gov/entrez/query.fcgi?db=gene&cmd=Retrieve&dopt=full_report&list_uids=101958055) | Paqr4 | -2.19052 | -4.56469 | 2.4E-05 | 0.001395 |
| [101968280](https://www.ncbi.nlm.nih.gov/entrez/query.fcgi?db=gene&cmd=Retrieve&dopt=full_report&list_uids=101968280) | Gpd1l | -2.19616 | -4.58258 | 1.2E-10 | 3.08E-08 |
| [101965820](https://www.ncbi.nlm.nih.gov/entrez/query.fcgi?db=gene&cmd=Retrieve&dopt=full_report&list_uids=101965820) | Sik1 | -2.22055 | -4.6607 | 2.06E-08 | 3.23E-06 |
| [101973761](https://www.ncbi.nlm.nih.gov/entrez/query.fcgi?db=gene&cmd=Retrieve&dopt=full_report&list_uids=101973761) | Postn | -2.22264 | -4.66748 | 0.000367 | 0.012276 |
| [101963811](https://www.ncbi.nlm.nih.gov/entrez/query.fcgi?db=gene&cmd=Retrieve&dopt=full_report&list_uids=101963811) | Pnrc1 | -2.24028 | -4.72489 | 3.33E-08 | 4.73E-06 |
| [101967428](https://www.ncbi.nlm.nih.gov/entrez/query.fcgi?db=gene&cmd=Retrieve&dopt=full_report&list_uids=101967428) | Fkbp5 | -2.24668 | -4.7459 | 5.8E-13 | 2.69E-10 |
| [101975661](https://www.ncbi.nlm.nih.gov/entrez/query.fcgi?db=gene&cmd=Retrieve&dopt=full_report&list_uids=101975661) | Nfil3 | -2.24936 | -4.7547 | 0.000572 | 0.0171 |
| [101968562](https://www.ncbi.nlm.nih.gov/entrez/query.fcgi?db=gene&cmd=Retrieve&dopt=full_report&list_uids=101968562) | Adamtsl2 | -2.26015 | -4.79042 | 0.000122 | 0.005442 |
| [101956400](https://www.ncbi.nlm.nih.gov/entrez/query.fcgi?db=gene&cmd=Retrieve&dopt=full_report&list_uids=101956400) | LOC101956400 | -2.26712 | -4.8136 | 3.52E-10 | 8.19E-08 |
| [101961849](https://www.ncbi.nlm.nih.gov/entrez/query.fcgi?db=gene&cmd=Retrieve&dopt=full_report&list_uids=101961849) | Itprip | -2.26926 | -4.82077 | 4.41E-05 | 0.002339 |
| [101961989](https://www.ncbi.nlm.nih.gov/entrez/query.fcgi?db=gene&cmd=Retrieve&dopt=full_report&list_uids=101961989) | Tsc22d3 | -2.31788 | -4.98599 | 5.86E-11 | 1.65E-08 |
| [101973082](https://www.ncbi.nlm.nih.gov/entrez/query.fcgi?db=gene&cmd=Retrieve&dopt=full_report&list_uids=101973082) | Klhl21 | -2.31873 | -4.98892 | 1.54E-13 | 8.46E-11 |
| [101969711](https://www.ncbi.nlm.nih.gov/entrez/query.fcgi?db=gene&cmd=Retrieve&dopt=full_report&list_uids=101969711) | Frat2 | -2.33336 | -5.03979 | 2.04E-10 | 5.04E-08 |
| [101976333](https://www.ncbi.nlm.nih.gov/entrez/query.fcgi?db=gene&cmd=Retrieve&dopt=full_report&list_uids=101976333) | Nabp1 | -2.36783 | -5.16166 | 2.11E-07 | 2.47E-05 |
| [101970857](https://www.ncbi.nlm.nih.gov/entrez/query.fcgi?db=gene&cmd=Retrieve&dopt=full_report&list_uids=101970857) | Kcnj14 | -2.38084 | -5.20838 | 4.89E-05 | 0.002515 |
| [101957628](https://www.ncbi.nlm.nih.gov/entrez/query.fcgi?db=gene&cmd=Retrieve&dopt=full_report&list_uids=101957628) | Slc26a7 | -2.38476 | -5.22256 | 7.82E-06 | 0.000546 |
| [101961955](https://www.ncbi.nlm.nih.gov/entrez/query.fcgi?db=gene&cmd=Retrieve&dopt=full_report&list_uids=101961955) | Cfap74 | -2.40355 | -5.29105 | 9.61E-05 | 0.004431 |
| [101956113](https://www.ncbi.nlm.nih.gov/entrez/query.fcgi?db=gene&cmd=Retrieve&dopt=full_report&list_uids=101956113) | Cxcr4 | -2.42369 | -5.36541 | 4.93E-06 | 0.000384 |
| [101964315](https://www.ncbi.nlm.nih.gov/entrez/query.fcgi?db=gene&cmd=Retrieve&dopt=full_report&list_uids=101964315) | LOC101964315 | -2.46334 | -5.51491 | 0 | 0 |
| [101978376](https://www.ncbi.nlm.nih.gov/entrez/query.fcgi?db=gene&cmd=Retrieve&dopt=full_report&list_uids=101978376) | Fntb | -2.46743 | -5.53057 | 3.5E-11 | 1.11E-08 |
| [101977672](https://www.ncbi.nlm.nih.gov/entrez/query.fcgi?db=gene&cmd=Retrieve&dopt=full_report&list_uids=101977672) | Slc25a33 | -2.56385 | -5.91283 | 1.33E-15 | 1.01E-12 |
| [101967141](https://www.ncbi.nlm.nih.gov/entrez/query.fcgi?db=gene&cmd=Retrieve&dopt=full_report&list_uids=101967141) | LOC101967141 | -2.58876 | -6.01582 | 5.33E-08 | 7.24E-06 |
| [101970431](https://www.ncbi.nlm.nih.gov/entrez/query.fcgi?db=gene&cmd=Retrieve&dopt=full_report&list_uids=101970431) | LOC101970431 | -2.59439 | -6.03935 | 3E-15 | 2.13E-12 |
| [101969612](https://www.ncbi.nlm.nih.gov/entrez/query.fcgi?db=gene&cmd=Retrieve&dopt=full_report&list_uids=101969612) | Rnf207 | -2.61759 | -6.13725 | 9.25E-07 | 9.01E-05 |
| [101974987](https://www.ncbi.nlm.nih.gov/entrez/query.fcgi?db=gene&cmd=Retrieve&dopt=full_report&list_uids=101974987) | Ip6k3 | -2.63765 | -6.22316 | 1.67E-12 | 6.43E-10 |
| [101977815](https://www.ncbi.nlm.nih.gov/entrez/query.fcgi?db=gene&cmd=Retrieve&dopt=full_report&list_uids=101977815) | Max | -2.67222 | -6.3741 | 0 | 0 |
| [101967306](https://www.ncbi.nlm.nih.gov/entrez/query.fcgi?db=gene&cmd=Retrieve&dopt=full_report&list_uids=101967306) | Cebpd | -2.68008 | -6.40892 | 5.73E-11 | 1.65E-08 |
| [101977121](https://www.ncbi.nlm.nih.gov/entrez/query.fcgi?db=gene&cmd=Retrieve&dopt=full_report&list_uids=101977121) | Mcph1 | -2.68921 | -6.44958 | 1.86E-05 | 0.001129 |
| [101975528](https://www.ncbi.nlm.nih.gov/entrez/query.fcgi?db=gene&cmd=Retrieve&dopt=full_report&list_uids=101975528) | Pitx3 | -2.73708 | -6.66717 | 6.1E-09 | 1.02E-06 |
| [101956579](https://www.ncbi.nlm.nih.gov/entrez/query.fcgi?db=gene&cmd=Retrieve&dopt=full_report&list_uids=101956579) | Ybx2 | -2.76109 | -6.77908 | 1.21E-07 | 1.55E-05 |
| [101963246](https://www.ncbi.nlm.nih.gov/entrez/query.fcgi?db=gene&cmd=Retrieve&dopt=full_report&list_uids=101963246) | Sgk1 | -2.78312 | -6.88338 | 1.93E-13 | 1.01E-10 |
| [101976940](https://www.ncbi.nlm.nih.gov/entrez/query.fcgi?db=gene&cmd=Retrieve&dopt=full_report&list_uids=101976940) | Hif3a | -2.85274 | -7.2237 | 6.61E-05 | 0.003211 |
| [101962590](https://www.ncbi.nlm.nih.gov/entrez/query.fcgi?db=gene&cmd=Retrieve&dopt=full_report&list_uids=101962590) | Atf3 | -2.94023 | -7.67535 | 8.22E-15 | 5.52E-12 |
| [101956126](https://www.ncbi.nlm.nih.gov/entrez/query.fcgi?db=gene&cmd=Retrieve&dopt=full_report&list_uids=101956126) | Hspa1a | -2.98406 | -7.91209 | 2.99E-10 | 7.2E-08 |
| [101956216](https://www.ncbi.nlm.nih.gov/entrez/query.fcgi?db=gene&cmd=Retrieve&dopt=full_report&list_uids=101956216) | Nrg4 | -3.04056 | -8.22813 | 3.03E-13 | 1.52E-10 |
| [101974891](https://www.ncbi.nlm.nih.gov/entrez/query.fcgi?db=gene&cmd=Retrieve&dopt=full_report&list_uids=101974891) | Slc7a8 | -3.04111 | -8.23122 | 5.56E-14 | 3.36E-11 |
| [101964964](https://www.ncbi.nlm.nih.gov/entrez/query.fcgi?db=gene&cmd=Retrieve&dopt=full_report&list_uids=101964964) | Btg2 | -3.04949 | -8.27918 | 3.94E-08 | 5.47E-06 |
| [101955831](https://www.ncbi.nlm.nih.gov/entrez/query.fcgi?db=gene&cmd=Retrieve&dopt=full_report&list_uids=101955831) | Hspa1b | -3.06152 | -8.34851 | 2.18E-12 | 7.97E-10 |
| [101954642](https://www.ncbi.nlm.nih.gov/entrez/query.fcgi?db=gene&cmd=Retrieve&dopt=full_report&list_uids=101954642) | Cish | -3.07405 | -8.42132 | 2.34E-08 | 3.54E-06 |
| [101976847](https://www.ncbi.nlm.nih.gov/entrez/query.fcgi?db=gene&cmd=Retrieve&dopt=full_report&list_uids=101976847) | Gpr157 | -3.11113 | -8.64058 | 2.22E-14 | 1.41E-11 |
| [101975062](https://www.ncbi.nlm.nih.gov/entrez/query.fcgi?db=gene&cmd=Retrieve&dopt=full_report&list_uids=101975062) | Errfi1 | -3.28915 | -9.77534 | 0 | 0 |
| [101955057](https://www.ncbi.nlm.nih.gov/entrez/query.fcgi?db=gene&cmd=Retrieve&dopt=full_report&list_uids=101955057) | Idi2 | -3.39769 | -10.5392 | 0 | 0 |
| [101970140](https://www.ncbi.nlm.nih.gov/entrez/query.fcgi?db=gene&cmd=Retrieve&dopt=full_report&list_uids=101970140) | LOC101970140 | -3.4146 | -10.6635 | 0.001349 | 0.032122 |
| [101965267](https://www.ncbi.nlm.nih.gov/entrez/query.fcgi?db=gene&cmd=Retrieve&dopt=full_report&list_uids=101965267) | Arrdc2 | -3.4573 | -10.9837 | 1.7E-12 | 6.43E-10 |
| [101956852](https://www.ncbi.nlm.nih.gov/entrez/query.fcgi?db=gene&cmd=Retrieve&dopt=full_report&list_uids=101956852) | Oxct1 | -3.47496 | -11.119 | 0 | 0 |
| [101959609](https://www.ncbi.nlm.nih.gov/entrez/query.fcgi?db=gene&cmd=Retrieve&dopt=full_report&list_uids=101959609) | LOC101959609 | -3.89507 | -14.8776 | 0.000426 | 0.013613 |
| [101965427](https://www.ncbi.nlm.nih.gov/entrez/query.fcgi?db=gene&cmd=Retrieve&dopt=full_report&list_uids=101965427) | Lipg | -4.20309 | -18.4186 | 0 | 0 |
| [101969412](https://www.ncbi.nlm.nih.gov/entrez/query.fcgi?db=gene&cmd=Retrieve&dopt=full_report&list_uids=101969412) | LOC101969412 | -4.40092 | -21.1256 | 0 | 0 |
| [101975221](https://www.ncbi.nlm.nih.gov/entrez/query.fcgi?db=gene&cmd=Retrieve&dopt=full_report&list_uids=101975221) | Ddit4 | -4.59003 | -24.0844 | 0 | 0 |

**Table 2S.** Gene set enrichment for Gene Ontology biological processes, Reactome, KEGG, Hallmark and Biocarta gene sets in muscle of arctic ground squirrels during hibernation (ET- entering torpor, LT-late torpor) as compared to summer active (SA) animals. NES – normalized enrichment score, positive values indicate elevated proportion of overexpressed genes and negative scores indicate excess of under expressed genes. FDR – false discovery rate with cutoff FDR < 0.05. Selected pathways are presented in Table 3.

| Comparison | ET vs. SA | | | LT vs. SA | | |
| --- | --- | --- | --- | --- | --- | --- |
| Gene Set | #genes | NES | FDR | #genes | NES | FDR |
| Hallmark |  |  |  |  |  |  |
| Epithelial Mesenchymal Transition | 158 | -2.81 | <0.001 |  |  |  |
| Hypoxia | 164 | -2.25 | 0.010 |  |  |  |
| UV Response Up | 117 | -2.06 | 0.027 |  |  |  |
| Complement | 131 | -2.04 | 0.027 |  |  |  |
| Coagulation | 81 | -1.93 | 0.040 | 83 | 2.06 | 0.013 |
| Oxidative Phosphorylation | 167 | 2.61 | 0.001 | 167 | 2.94 | <0.001 |
| MYC TargersV1 | 190 | 2.10 | 0.021 | 190 | 2.87 | <0.001 |
| KRAS Signaling DN | 80 | 1.91 | 0.024 | 81 | 1.98 | 0.016 |
| Mitotic Spindle |  |  |  | 175 | -2.17 | 0.021 |
| Xenobiotic Metabolism |  |  |  | 140 | 3.12 | <0.001 |
| Protein Secretion |  |  |  | 90 | 2.95 | <0.001 |
| Interferon Alpha Response |  |  |  | 69 | 2.22 | 0.008 |
| Interferon Gamma Response |  |  |  | 139 | 2.16 | 0.010 |
| Estrogen Response Late |  |  |  | 135 | 2.10 | 0.012 |
| MTORC1 Signaling |  |  |  | 180 | 1.99 | 0.016 |
| Cholesterol Homeostasis |  |  |  | 63 | 1.86 | 0.029 |
| Bile Acid Metabolism |  |  |  | 69 | 1.76 | 0.045 |
| KEGG |  |  |  |  |  |  |
| Complement and Coagulation Cascades | 30 | -2.53 | 0.010 | 32 | 2.67 | 0.001 |
| Peroxisome | 66 | 2.30 | 0.022 | 67 | 2.37 | 0.010 |
| Oxidative Phosphorylation | 75 | 2.28 | 0.017 | 76 | 2.19 | 0.021 |
| Fatty Acid Metabolism |  |  |  | 29 | 2.65 | <0.001 |
| Proteasome |  |  |  | 41 | 2.48 | 0.004 |
| Pyrimidine Metabolism |  |  |  | 81 | 2.37 | 0.008 |
| Steroid Biosynthesis |  |  |  | 10 | 2.28 | 0.014 |
| Reactome |  |  |  |  |  |  |
| Translation | 137 | 4.20 | <0.001 | 137 | 5.20 | <0.001 |
| SRP Dependent Cotranslational Protein Targeting to Membrane | 102 | 4.13 | <0.001 | 102 | 5.10 | <0.001 |
| Peptide Chane Elongation | 79 | 4.06 | <0.001 | 79 | 4.27 | <0.001 |
| Metabolism of Proteins | 351 | 4.01 | <0.001 |  |  |  |
| Influenza Viral RNA Transcription and Replication | 95 | 3.86 | <0.001 | 95 | 4.22 | <0.001 |
| 3-UTR Mediated Translational Regulation | 98 | 3.85 | <0.001 | 98 | 4.38 | <0.001 |
| Influenza Life Cycle | 127 | 3.37 | <0.001 | 127 | 3.51 | <0.001 |
| Nonsense Mediated Decay Enhanced by the Exon Junction Complex | 100 | 3.30 | <0.001 | 100 | 3.90 | <0.001 |
| Formation of the Ternary Complex and Subsequently the 43S Complex | 45 | 3.13 | <0.001 | 45 | 2.84 | <0.001 |
| Metabolism of mRNA | 201 | 3.00 | <0.001 | 201 | 3.44 | <0.001 |
| Metabolism of RNA | 241 | 2.89 | <0.001 | 242 | 3.16 | <0.001 |
| Activation of the mRNA upon Binding of the Cap Binding Complex and Eifs and Subsequent Binding to 43S | 53 | 2.54 | 0.003 | 53 | 2.89 | <0.001 |
| Respiratory Electron Transport ATP Synthesis by Chemiosmotic Coupling and Heat Production by Uncoupling Proteins | 59 | 2.41 | 0.006 |  |  |  |
| Mitotic G2 G2 M Phases | 69 | 2.37 | 0.008 |  |  |  |
| Recruitment of Mitotic Centrosome Proteins and Complexes | 58 | 2.23 | 0.020 |  |  |  |
| Autodegradation of CDH1 by CDH1 APC C | 55 | 2.22 | 0.021 | 55 | 3.05 | <0.001 |
| Synthesis of PA | 15 | 2.21 | 0.020 |  |  |  |
| Mitochondrial Protein Import | 44 | 2.19 | 0.022 |  |  |  |
| APC C CDC20 Mediated Degradation of Mitotic Proteins | 59 | 2.16 | 0.025 | 59 | 2.77 | <0.001 |
| APC C CDH1 Mediated Degradation of CDC20 and Other APC C CDH1 Targeted Proteins in Late Mitosis Early G1 | 59 | 2.11 | 0.034 | 59 | 2.65 | 0.001 |
| Regulation of Apoptosis | 53 | 2.11 | 0.033 | 53 | 2.73 | <0.001 |
| Respiratory Electron Transport | 44 | 2.07 | 0.042 |  |  |  |
| Regulation of Mitotic Cell Cycle | 67 | 2.06 | 0.043 | 67 | 2.21 | 0.008 |
| Loss of NLP from Mitotic Centrosomes | 53 | 2.04 | 0.045 |  |  |  |
| Neuronal System | 136 | 2.03 | 0.046 |  |  |  |
| VIF Mediated Degradation of APOBEC3G | 47 | 2.02 | 0.047 | 47 | 2.88 | <0.001 |
| HS GAG Biosynthesis | 19 | 2.02 | 0.045 |  |  |  |
| Collagen Formation |  |  |  | 37 | -2.55 | 0.016 |
| NCAM1 Interactions |  |  |  | 24 | -2.47 | 0.020 |
| Striated Muscle Contraction |  |  |  | 25 | -2.37 | 0.026 |
| Respiratory Electron Transport ATP Synthesis by Chemiosmotic Coupling and Heat Production by Uncoupling Proteins |  |  |  | 59 | -2.34 | 0.025 |
| TCA Cycle and Respiratory Electron Transport |  |  |  | 94 | -2.34 | 0.022 |
| Generic Transcription Pathway |  |  |  | 180 | -2.20 | 0.048 |
| Metabolism of Proteins |  |  |  | 353 | 5.20 | <0.001 |
| Initial Triggering of Complement |  |  |  | 11 | 3.17 | <0.001 |
| Complement Cascade |  |  |  | 16 | 2.93 | <0.001 |
| Prefoldin Mediated Transfer of Substrate to CCT TRIC |  |  |  | 22 | 2.86 | <0.001 |
| P53 Independent G1 S DNA Damage Checkpoint |  |  |  | 45 | 2.74 | <0.001 |
| ER Phagosome Pathway |  |  |  | 52 | 2.68 | 0.001 |
| CDK Mediated Phosphorylation and Removal of CDC6 |  |  |  | 44 | 2.67 | <0.001 |
| Class I MHC Mediated Antigen Processing Presentation |  |  |  | 212 | 2.63 | 0.001 |
| SCF Beta TRCP Mediated Degradation of EMI1 |  |  |  | 46 | 2.61 | 0.001 |
| CDT1 Association with the CDC6 ORC Origin Complex |  |  |  | 50 | 2.60 | 0.001 |
| Antigen Processing Cross Presentation |  |  |  | 61 | 2.58 | 0.001 |
| Host Interactions of HIV Factors |  |  |  | 109 | 2.57 | 0.001 |
| M G1 Transition |  |  |  | 70 | 2.56 | 0.001 |
| Regulation of Ornithine Decarboxylase ODC |  |  |  | 45 | 2.53 | 0.001 |
| Synthesis of DNA |  |  |  | 80 | 2.49 | 0.002 |
| Post Chaperonin Tubulin Folding Pathway |  |  |  | 12 | 2.48 | 0.002 |
| Cross Presentation of Soluble Exogenous Antigens Endosomes |  |  |  | 43 | 2.45 | 0.002 |
| Autodegradation of the E3 Ubiquitin Ligase COP1 |  |  |  | 46 | 2.44 | 0.002 |
| Formation of Tubulin Folding Intermediates by CCT TRIC |  |  |  | 15 | 2.40 | 0.003 |
| Assembly of the Pre Replicative Complex |  |  |  | 57 | 2.36 | 0.004 |
| Metabolism of Amino Acids and Derivatives |  |  |  | 139 | 2.32 | 0.005 |
| Metabolism of Lipids and Lipoproteins |  |  |  | 345 | 2.31 | 0.005 |
| Antigen Processing Ubiquitination Proteasome Degradation |  |  |  | 185 | 2.31 | 0.005 |
| Adaptive Immune System |  |  |  | 394 | 2.27 | 0.007 |
| Synthesis of Very Long Chain Fatty Acyl Coas |  |  |  | 11 | 2.27 | 0.006 |
| Protein Folding |  |  |  | 44 | 2.25 | 0.007 |
| Destabilization of mRNA by AUF1 HNRNP D0 |  |  |  | 49 | 2.25 | 0.007 |
| ORC1 Removal from Chromatin |  |  |  | 58 | 2.24 | 0.007 |
| SCFSKP2 Mediated Degradation of P27 P21 |  |  |  | 51 | 2.23 | 0.008 |
| S Phase |  |  |  | 95 | 2.21 | 0.009 |
| Peptide Ligand Binding Receptors |  |  |  | 26 | 2.20 | 0.009 |
| Activation of NF KAPPAB in B Cells |  |  |  | 57 | 2.15 | 0.012 |
| Cell Cycle Checkpoints |  |  |  | 97 | 2.11 | 0.015 |
| HIV Infection |  |  |  | 175 | 2.09 | 0.016 |
| Diabetes Pathways |  |  |  | 101 | 2.09 | 0.01 |
| Cyclin E Associated Events During G1 S Transition |  |  |  | 60 | 2.07 | 0.018 |
| Fatty Acyl COA Biosynthesis |  |  |  | 15 | 2.05 | 0.021 |
| Signaling by WNT |  |  |  | 61 | 2.02 | 0.024 |
| Biological Oxidations |  |  |  | 36 | 1.99 | 0.029 |
| Antigen Presentation Folding Assembly and Peptide Loading of Class I MHC |  |  |  | 15 | 1.92 | 0.041 |
| The Role of NEF in HIV1 Replication and Disease Pathogenesis |  |  |  | 20 | 1.92 | 0.041 |
| Downstream Signaling Events of B Cell Receptor BCR |  |  |  | 86 | 1.91 | 0.044 |
| Triglyceride Biosynthesis |  |  |  | 32 | 1.89 | 0.049 |
| Cytosolic tRNA Aminoacylation |  |  |  | 24 | 1.88 | 0.048 |
| P53 Dependent G1 DNA Damage Response |  |  |  | 52 | 1.88 | 0.049 |
| Gene Ontology |  |  |  |  |  |  |
| Actin Filament Based Process | 361 | -3.04 | 0.003 |  |  |  |
| Organonitrogen Compound Catabolic Process | 224 | -2.84 | 0.006 |  |  |  |
| Muscle System Process | 218 | -2.69 | 0.021 | 325 | -2.90 | 0.008 |
| mRNA Processing | 363 | -2.65 | 0.024 |  |  |  |
| Extracellular Structure Organization | 193 | -2.58 | 0.032 |  |  |  |
| Negative Regulation of Cell Adhesion | 144 | -2.54 | 0.040 |  |  |  |
| Response to Arsenic Containing Substance | 23 | -2.54 | 0.035 |  |  |  |
| Import into Cell | 24 | -2.49 | 0.041 |  |  |  |
| RNA Splicing | 315 | -2.48 | 0.040 | 373 | -2.37 | 0.042 |
| Muscle Contraction | 179 | -2.46 | 0.043 | 249 | -2.55 | 0.024 |
| Nuclear Transport | 295 | -2.43 | 0.047 |  |  |  |
| Protein Localization to Endoplasmic Reticulum | 116 | 4.06 | <0.001 | 126 | 4.03 | <0.001 |
| Establishment of Protein Localization to Endoplasmic Reticulum | 99 | 3.85 | <0.001 | 105 | 4.26 | <0.001 |
| Multi Organism Metabolic Process | 129 | 3.55 | <0.001 |  |  |  |
| Peptide Metabolic Process | 458 | 3.42 | <0.001 |  |  |  |
| Amide Metabolic Process | 424 | 3.37 | <0.001 |  |  |  |
| Nuclear Transcribed mRNA Catabolic Process Nonsense Mediated Decay | 113 | 3.28 | <0.001 | 112 | 3.62 | <0.001 |
| Synaptic Transmission Cholinergic | 10 | 2.86 | 0.002 |  |  |  |
| Protein Targeting to Membrane | 145 | 2.84 | 0.002 | 167 | 3.96 | <0.001 |
| Viral Life Cycle | 241 | 2.75 | 0.004 |  |  |  |
| Mitochondrion Organization | 473 | 2.64 | 0.007 | 390 | 2.39 | 0.017 |
| Anion Transmembrane Transport | 121 | 2.62 | 0.009 |  |  |  |
| Ion Transmembrane Transport | 390 | 2.60 | 0.009 |  |  |  |
| Neuron Projection Guidance | 123 | 2.41 | 0.035 |  |  |  |
| Establishment of Protein Localization to Organelle | 312 | 2.40 | 0.034 | 452 | 3.48 | <0.001 |
| Regulation of Peptide Transport | 141 | 2.37 | 0.042 |  |  |  |
| Muscle Tissue Development |  |  |  | 301 | -2.96 | 0.010 |
| Muscle Structure Development |  |  |  | 475 | -2.78 | 0.014 |
| Skeletal Muscle Cell Differentiation |  |  |  | 53 | -2.74 | 0.015 |
| Response to Muscle Strength |  |  |  | 18 | -2.70 | 0.016 |
| Protein Demethylation |  |  |  | 26 | -2.60 | 0.024 |
| Skeletal Muscle Organ Development |  |  |  | 133 | -2.60 | 0.022 |
| Sarcomere Organization |  |  |  | 40 | -2.58 | 0.022 |
| Muscle Organ Development |  |  |  | 295 | -2.53 | 0.027 |
| Striated Muscle Adaptation |  |  |  | 41 | -2.52 | 0.026 |
| Covalent Chromatin Modification |  |  |  | 366 | -2.51 | 0.025 |
| Myofibril Assembly |  |  |  | 60 | -2.51 | 0.024 |
| Negative Regulation of Muscle Cell Apoptotic Process |  |  |  | 25 | -2.50 | 0.025 |
| Actin Filament Based Movement |  |  |  | 108 | -2.49 | 0.023 |
| Actin Mediated Cell Contraction |  |  |  | 89 | -2.49 | 0.022 |
| Muscle Cell Differentiation |  |  |  | 269 | -2.47 | 0.024 |
| Actomyosin Structure Organization |  |  |  | 158 | -2.46 | 0.026 |
| Peptidyl Lysine Modification |  |  |  | 313 | -2.43 | 0.031 |
| Regulation of Tube Size |  |  |  | 78 | -2.40 | 0.037 |
| Muscle Cell Development |  |  |  | 146 | -2.35 | 0.045 |
| Regulation of Transcription from RNA Polymerase II Promoter in Response to Oxidative Stress |  |  |  | 11 | -2.34 | 0.048 |
| ATP Synthesis Coupled Electron Transport |  |  |  | 53 | -2.32 | 0.048 |
| Protein Targeting |  |  |  | 354 | 4.38 | <0.001 |
| Cotranslational Protein Targeting to Membrane |  |  |  | 92 | 4.11 | <0.001 |
| Viral Gene Expression |  |  |  | 176 | 3.52 | <0.001 |
| Monocarboxylic Acid Catabolic Process |  |  |  | 94 | 3.22 | <0.001 |
| Establishment of Protein Localization to Membrane |  |  |  | 267 | 3.20 | <0.001 |
| Organic Hydroxy Compound Biosynthetic Process |  |  |  | 162 | 3.15 | <0.001 |
| Steroid Metabolic Process |  |  |  | 167 | 3.14 | <0.001 |
| Cytoplasmic Translation |  |  |  | 81 | 3.12 | <0.001 |
| Organic Hydroxy Compound Metabolic Process |  |  |  | 276 | 3.10 | <0.001 |
| Regulation of Small Molecule Metabolic Process |  |  |  | 254 | 3.03 | <0.001 |
| Alcohol Biosynthetic Process |  |  |  | 120 | 2.95 | <0.001 |
| Complement Activation |  |  |  | 28 | 2.94 | <0.001 |
| Small Molecule Biosynthetic Process |  |  |  | 457 | 2.92 | 0.001 |
| Positive Regulation of Immune Response |  |  |  | 450 | 2.83 | 0.001 |
| Small Molecule Catabolic Process |  |  |  | 266 | 2.82 | 0.001 |
| Fatty Acid Catabolic Process |  |  |  | 84 | 2.81 | 0.001 |
| Sterol Biosynthetic Process |  |  |  | 56 | 2.76 | 0.002 |
| Humoral Immune Response |  |  |  | 68 | 2.76 | 0.002 |
| Leukocyte Mediated Immunity |  |  |  | 475 | 2.75 | 0.002 |
| Protein Localization to Membrane |  |  |  | 477 | 2.73 | 0.002 |
| Myeloid Leukocyte Mediated Immunity |  |  |  | 372 | 2.70 | 0.002 |
| Antigen Processing and Presentation of Peptide Antigen via MHC Class I |  |  |  | 79 | 2.70 | 0.002 |
| Endoplasmic Reticulum to Golgi Vesicle Mediated Transport |  |  |  | 168 | 2.69 | 0.002 |
| Myeloid Leukocyte Activation |  |  |  | 420 | 2.69 | 0.002 |
| Nuclear Transcribed mRNA Catabolic Process |  |  |  | 183 | 2.68 | 0.002 |
| Regulation of Steroid Metabolic Process |  |  |  | 81 | 2.67 | 0.003 |
| Mitochondrial Translation |  |  |  | 128 | 2.66 | 0.003 |
| Golgi Vesicle Transport |  |  |  | 294 | 2.65 | 0.003 |
| RNA Catabolic Process |  |  |  | 332 | 2.63 | 0.004 |
| Antigen Processing and Presentation of Exogenous Peptide Antigen via MHC Class I |  |  |  | 64 | 2.63 | 0.004 |
| Mitochondrial Gene Expression |  |  |  | 450 | 2.57 | 0.006 |
| Steroid Biosynthetic Process |  |  |  | 115 | 2.57 | 0.005 |
| Monocarboxylic Acid Metabolic Process |  |  |  | 293 | 2.53 | 0.008 |
| Regulation of Humoral Immune Response |  |  |  | 29 | 2.50 | 0.009 |
| Monocarboxylic Acid Transport |  |  |  | 82 | 2.50 | 0.009 |
| Organic Acid Catabolic Process |  |  |  | 178 | 2.49 | 0.009 |
| Lipid Localization |  |  |  | 224 | 2.46 | 0.011 |
| Regulation of Immune Effector Process |  |  |  | 216 | 2.45 | 0.012 |
| Antigen Processing and Presentation |  |  |  | 154 | 2.45 | 0.012 |
| Innate Immune Response |  |  |  | 483 | 2.42 | 0.014 |
| Neutral Lipid Biosynthetic Process |  |  |  | 31 | 2.39 | 0.017 |
| Humoral Immune Response Mediated by Circulating Immunoglobulin |  |  |  | 24 | 2.38 | 0.018 |
| Viral Translation |  |  |  | 14 | 2.36 | 0.020 |
| Mitochondrial Translation Termination |  |  |  | 86 | 2.36 | 0.019 |
| Positive Regulation of Immune Effector Process |  |  |  | 110 | 2.35 | 0.019 |
| Leukocyte Mediated Cytotoxicity |  |  |  | 43 | 2.35 | 0.020 |
| Ribonucleoprotein Complex Subunit Organization |  |  |  | 213 | 2.33 | 0.023 |
| Interleukin 1 Mediated Signaling Pathway |  |  |  | 87 | 2.33 | 0.022 |
| Cellular Lipid Catabolic Process |  |  |  | 144 | 2.33 | 0.022 |
| Organic Acid Biosynthetic Process |  |  |  | 182 | 2.32 | 0.022 |
| Anaphase Promoting Complex Dependent Catabolic Process |  |  |  | 70 | 2.32 | 0.022 |
| Peroxisome Organization |  |  |  | 64 | 2.32 | 0.022 |
| Antigen Processing and Presentation of Peptide Antigen |  |  |  | 133 | 2.32 | 0.021 |
| Fatty Acid Metabolic Process |  |  |  | 169 | 2.32 | 0.021 |
| Lipid Biosynthetic Process |  |  |  | 458 | 2.32 | 0.022 |
| Positive Regulation of Establishment of Protein Localization |  |  |  | 291 | 2.31 | 0.022 |
| Regulation of Cellular Ketone Metabolic Process |  |  |  | 111 | 2.31 | 0.022 |
| Regulation of Establishment of Protein Localization |  |  |  | 489 | 2.28 | 0.026 |
| Cell Killing |  |  |  | 50 | 2.27 | 0.027 |
| Positive Regulation of Steroid Biosynthetic Process |  |  |  | 11 | 2.27 | 0.027 |
| Regulation of Cellular Amino Acid Metabolic Process |  |  |  | 51 | 2.27 | 0.028 |
| Ribonucleoprotein Complex Biogenesis |  |  |  | 395 | 2.25 | 0.030 |
| Protein Localization to Nucleoplasm |  |  |  | 10 | 2.24 | 0.033 |
| Lipid Catabolic Process |  |  |  | 196 | 2.23 | 0.034 |
| Activation of Immune Response |  |  |  | 370 | 2.22 | 0.035 |
| Protein Folding |  |  |  | 182 | 2.22 | 0.035 |
| Regulation of Interleukin 4 Production |  |  |  | 11 | 2.21 | 0.037 |
| Organic Cyclic Compound Catabolic Process |  |  |  | 462 | 2.21 | 0.037 |
| Neutral Lipid Metabolic Process |  |  |  | 76 | 2.20 | 0.038 |
| Regulation of Establishment of Planar Polarity |  |  |  | 89 | 2.20 | 0.039 |
| Cytoplasmic Translational Initiation |  |  |  | 26 | 2.20 | 0.038 |
| Telomerase RNA Localization |  |  |  | 19 | 2.20 | 0.038 |
| Fructose Metabolic Process |  |  |  | 10 | 2.20 | 0.038 |
| Positive Regulation of Establishment of Protein Localization to Telomere |  |  |  | 10 | 2.20 | 0.038 |
| Sterol Metabolic Process |  |  |  | 51 | 2.19 | 0.040 |
| Positive Chemotaxis |  |  |  | 40 | 2.18 | 0.040 |
| Maternal Process Involved in Female Pregnancy |  |  |  | 39 | 2.18 | 0.040 |
| Non Canonical WNT Signaling Pathway |  |  |  | 122 | 2.17 | 0.041 |
| Amine Metabolic Process |  |  |  | 88 | 2.17 | 0.041 |
| Cellular Protein Complex Disassembly |  |  |  | 182 | 2.17 | 0.041 |
| Positive Regulation of Defense Response |  |  |  | 284 | 2.17 | 0.041 |
| Vesicle Organization |  |  |  | 255 | 2.17 | 0.041 |
| Integrin Activation |  |  |  | 15 | 2.17 | 0.041 |
| Peptide Secretion |  |  |  | 329 | 2.16 | 0.042 |
| Mitochondrial Transport |  |  |  | 199 | 2.16 | 0.041 |
| Serine Family Amino Acid Biosynthetic Process |  |  |  | 14 | 2.16 | 0.042 |
| Regulation of Insulin Secretion |  |  |  | 104 | 2.16 | 0.042 |
| Hematopoietic Stem Cell Differentiation |  |  |  | 68 | 2.16 | 0.042 |
| Positive Regulation of Steroid Metabolic Process |  |  |  | 15 | 2.15 | 0.042 |
| Adaptive Immune Response Based on Somatic Recombination of Immune Receptors Built from Immunoglobulin Superfamily Domains |  |  |  | 137 | 2.15 | 0.044 |
| Lymphocyte Mediated Immunity |  |  |  | 122 | 2.15 | 0.043 |
| Biocarta |  |  |  |  |  |  |
| Complement Pathway |  |  |  | 13 | 3.06 | 0.000 |
| Proteasome Pathway |  |  |  | 28 | 2.29 | 0.042 |
